# Supplementary material for: Scenario-based forecasting of the global energy demand and carbon footprint of artificial intelligence
Source: PLoS One. 2026 Mar 11;21(3):e0343056. doi: 10.1371/journal.pone.0343056 (PMC12978478; doi:10.1371/journal.pone.0343056)
Supplement: S1 File — (PDF) [file pone.0343056.s001.pdf]

Supplementary information: *"Scenario-based forecasting of the global energy demand and carbon footprint of artificial intelligence"*

Contents

1 Overview: Energy Demand of AI **2**

2 Energy Efficiency **3**
- 2.1 Hardware Efficiency . . . 4
- 2.2 Algorithmic Efficiency . . . 5
- 2.3 Power Usage Effectiveness . . . 8

3 Computational Demand **10**
- 3.1 Overview . . . 10
- 3.2 Training Demand . . . 11
  - 3.2.1 Model Complexity . . . 11
  - 3.2.2 Model Demand . . . 14
- 3.3 Usage Demand . . . 16
  - 3.3.1 Country Groupings by Development Level . . . 16
  - 3.3.2 Population Growth . . . 18
  - 3.3.3 Adoption Rate . . . 20
- 3.4 Query Complexity . . . 22

4 Total Power **25**
- 4.1 Historical Trends in Global Power Consumption . . . 25
  - 4.1.1 Comprehensive Formula used in this Study . . . 26
  - 4.1.2 Breakdown of AI Energy Usage . . . 27
- 4.2 Scenarios . . . 27
  - 4.2.1 Baseline . . . 27
  - 4.2.2 Fewer, Larger Models . . . 28
  - 4.2.3 More, Smaller Models . . . 29
- 4.3 AI Energy Share of Total Global Energy . . . 29

5 Overview: Carbon Footprint of AI **30**

6 Supply Chain Carbon Footprint **30**
- 6.1 Manufacturing . . . 30
- 6.2 Transportation . . . 32

7 Electricity Carbon Footprint **34**
- 7.1 Cost of Electricity Generation . . . 35
  - 7.1.1 Step 1: Energy Pricing . . . 35
  - 7.1.2 Multi-Regional Input-Output Model . . . 35

7.1.3 Step 2: Emission Factor 40
7.2 Energy Demand 43
7.2.1 Step 3: Energy Demand Allocation 43
7.2.2 Step 4: Renewable and Non-Renewable Energy Distribution 45
7.2.3 Step 4.1A: Energy Demand Distribution by Scenario for 2030 46
7.2.4 Step 4.1B: Energy Demand Distribution by Scenario for 2050 49
7.2.5 Step 4.2: Allocation of Renewable and Non-Renewable Energy by Fuel Type 51
7.3 Total Carbon Footprint 52
7.3.1 Step 4.3A: Energy Distribution by Fuel Type for 2030 Projections 52
7.3.2 Step 4.3b: Allocation of Renewable and Non-Renewable Energy and Energy Distribution by Fuel Type for 2050 54
7.3.3 Step 4.4A: Calculation of Total Carbon Emissions for 2030 56
7.3.4 Step 4.4B: Calculation of Total Carbon Emissions for 2050 61

8 Tables and Figures **64**
8.1 Energy Pricing 64
8.2 Emission Factor 64
8.3 Energy Demand 65
8.4 Renewable and Non-Renewable Energy Split 65
8.5 Energy Demand by Fuel Type 65
8.6 Total Carbon Footrpint 66
8.7 Flowchart Representation of the Methodology 66

9 Overall Combined CO_2 Emission Results **70**
9.1 Scenario 1: Baseline 70
9.2 Scenario 2: Fewer Larger Models 70
9.3 Scenario 3: More Smaller Models 71

10 Conclusion **72**
10.1 Scenario 1: Baseline 72
10.2 Scenario 2: Fewer Larger Models 72
10.3 Scenario 3: More Smaller Models 73
10.4 Final Considerations 74

11 Business As Usual Results and Discussion **75**

A Appendix **82**

1. Overview: Energy Demand of AI

The energy demand of AI is proportional to the ratio of computational demand to energy efficiency and which, which is the total energy demand of AI per year. Energy efficiency encompasses three key factors: hardware efficiency, which reflects improvements in GPU energy consumption; algorithmic efficiency, which captures advances in optimizing computational workloads; and power usage effectiveness (PUE), which measures improvements in the operational efficiency of data

centers. These factors collectively reduce the power required per floating point operation (FLOP).

On the other hand, computational demand defines the total FLOPs required annually and consists of two main components: training demand and usage demand. Training demand depends on model complexity, representing the computational cost per AI model, and model demand, which measures the number of new AI models developed each year. Meanwhile, usage demand scales with AI users, which is projected based on population growth and adoption rates, and query complexity, representing the computational intensity of AI inference workloads.

Together, these factors determine the long-term trajectory of AI's energy demand, where improvements in efficiency may offset rising computational demand, or conversely, exponential growth in model usage and complexity could drive energy consumption beyond sustainable levels.

2. Energy Efficiency

Energy efficiency in the context of AI refers to the amount of computational work that can be performed per unit of energy consumed. This is a critical factor in assessing the long-term sustainability of AI systems, as both training and inference workloads demand substantial energy resources. In this study, we define energy efficiency using watts per floating-point operation (W/FLOP) as the fundamental unit. This metric was chosen because it provides an architecture-agnostic measure of computational efficiency, allowing direct comparisons across different AI models, hardware platforms, and data center infrastructures. Unlike task-specific energy metrics such as energy per model or energy per query, W/FLOP captures the underlying efficiency improvements driven by advances in hardware, software, and cooling technologies, making it a robust metric for long-term projections.

Previous studies have evaluated AI energy efficiency using a variety of approaches. Strubell et al. [1] quantified energy consumption per training cycle, assessing the carbon cost of model development based on energy usage recorded at specific training intervals. Patterson et al. [2] extended this approach by incorporating energy efficiency trends in hardware, analyzing the transition from general-purpose GPUs to AI-optimized accelerators such as tensor processing units (TPUs). While these studies provided valuable insights into energy consumption at discrete points in time, they did not develop a continuous model to project long-term efficiency trends under different technological and policy scenarios.

Our study improves upon these methodologies by incorporating a dynamic efficiency model that accounts for changes in hardware, algorithmic optimization, and infrastructure improvements over time. Instead of treating energy efficiency as a fixed value, we model it as a function of hardware efficiency, algorithmic efficiency, and power usage effectiveness (PUE), each of which evolves based on historical trends and projected technological advancements. This approach enables a comprehensive, scenario-based analysis of how AI's energy demand will evolve, addressing the limitations of static efficiency estimates used in previous research. By integrating efficiency improvements into a life-cycle assessment framework, our model provides a more accurate projection of AI's long-term energy demand, offering critical insights for policymakers and industry

stakeholders.

2.1 Hardware Efficiency

Hardware efficiency refers to the energy required to perform a given amount of computation, specifically in the context of AI workloads. As AI models become more complex, their computational demands increase, making hardware efficiency a crucial factor in managing AI's energy demand. Improvements in hardware efficiency directly influence AI's sustainability by reducing the power required per FLOP, thereby mitigating the environmental impact of large-scale AI training and inference.

The fundamental unit used to measure hardware efficiency in this study is teraflops per watt (TFLOPs/W), representing the number of trillion floating-point operations that can be executed per watt of power. This unit was chosen because it provides a universal and architecture-agnostic measure of computational efficiency. Unlike task-specific energy metrics that depend on particular workloads or models, TFLOPs/W enables direct comparisons across different AI processors, including GPUs, TPUs, and other accelerators. Additionally, it aligns with hardware performance benchmarks commonly used in the computing industry, making it suitable for both historical analysis and future projections.

To model hardware efficiency trends over time, we utilized historical data from NVIDIA's GPU architectures. Specifically, we focused on the following data points:

- **2012**: The GeForce GTX 680, based on the Kepler architecture, achieved approximately 0.42 TFLOPs/W. This figure is derived from its single-precision performance of 3.09 TFLOPs and a typical board power of 195W. [3]
- **2017**: The GeForce GTX 1080, utilizing the Pascal architecture, reached around 0.78 TFLOPs/W, with a performance of 8.87 TFLOPs and a power consumption of 180W. [4]
- **2020**: The GeForce RTX 3080, built on the Ampere architecture, delivered approximately 1.4 TFLOPs/W, boasting a performance of 29.77 TFLOPs and a power draw of 320W. [5]

Our study builds upon prior research by modeling hardware efficiency dynamically using a logistic function. Unlike static efficiency estimates, this approach captures both the rapid early-stage improvements in AI hardware and the diminishing returns observed as technology matures. The logistic function used to model efficiency over time is given by:

$$E_h(t) = \frac{E_{h0}}{1 + \exp(-\alpha_h(t - t_0))} + \beta_h \quad (1)$$

where $E_h(t)$ represents hardware efficiency at time t, E_{h0} is the initial hardware efficiency, α_h is the growth rate of efficiency improvements, t_0 is the inflection point where growth slows, and β_h represents the efficiency saturation level. This function was selected because it effectively models the historical trend of hardware efficiency gains, which initially accelerate due to architectural innovations but eventually plateau due to physical and economic constraints.

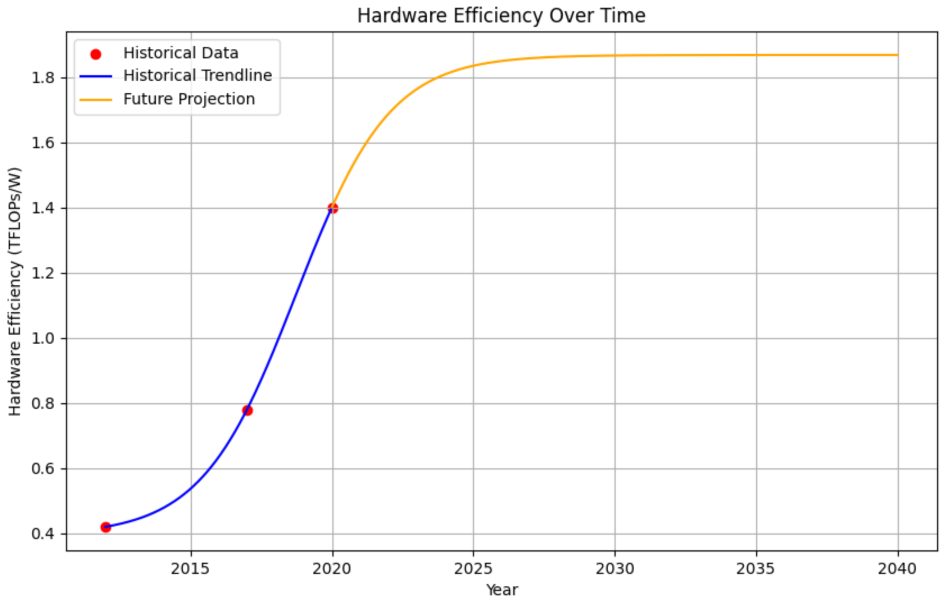

Figure 1: Projected hardware efficiency trends based on logistic modeling.

To estimate these parameters, we performed a logistic regression using historical efficiency data from 2012 to 2020. The curve fitting process involved optimizing the parameters E_{h0}, α_h, β_h, and t_0 to minimize the error between the model and observed values. The regression was implemented in Python using the SciPy optimization library.

The results of the regression, visualized in Figure 1, reveal key trends in hardware efficiency growth. Between 2012 and 2020, efficiency increased significantly, consistent with improvements in GPU and TPU architectures. The logistic model projects that efficiency gains will continue into the mid-2020s but will begin to saturate around 1.85 TFLOPs/W by 2040. This is consistent with empirical observations in semiconductor scaling, where factors such as power density and diminishing transistor improvements impose physical limits on efficiency growth.

These findings have important implications for AI sustainability. While past efficiency improvements have helped mitigate energy demands, our model suggests that gains will slow in the coming decades. This means that without complementary interventions, such as algorithmic efficiency improvements and the adoption of renewable energy sources, as hardware efficiency alone may be insufficient to curb AI's long-term energy demand. Future research should explore alternative computing paradigms, such as neuromorphic computing and quantum AI accelerators, to address the impending limitations of classical hardware efficiency scaling.

2.2 Algorithmic Efficiency

Algorithmic efficiency refers to the ability of optimization solvers to find feasible or optimal solutions with reduced computational effort over time. As AI-driven optimization problems grow

in complexity, improvements in algorithmic efficiency are crucial for maintaining computational feasibility while minimizing energy consumption. Unlike hardware efficiency, which measures power per floating-point operation, algorithmic efficiency captures how well optimization solvers reduce computational burden through improvements in heuristics, search strategies, and mathematical formulations.

The fundamental unit used in this study to measure algorithmic efficiency is the fraction of solved problems out of a standardized test set of 10,000 optimization problems. Specifically, we define efficiency as:

$$E_a(t) = \frac{10000 - U(t)}{10000} \tag{2}$$

where $U(t)$ represents the number of unsolved problems at time t. This unit was chosen because it provides a direct, interpretable measure of solver progress, independent of hardware improvements. By normalizing results within the same computational environment, we ensure that observed efficiency trends reflect genuine algorithmic advancements rather than hardware-driven speedups.

To quantify algorithmic efficiency trends, we utilized historical data from Gurobi's benchmark tests [7], where the number of unsolved problems out of 10,000 was recorded over time. The dataset includes:

Table 1: Number of unsolved Gurobi benchmark problems out of 10,000 over time.

Year	Unsolved Problems
2009	1655
2010	1511
2011	1343
2012	1265
2013	1134
2015	939
2017	575
2018	385
2020	319
2022	202
2023	173

These data points illustrate the steady improvement in solver effectiveness, reflecting enhancements in optimization algorithms, cutting-plane methods, and parallelization techniques. Research by [6] analyzed trends in mixed-integer programming (MIP) solver efficiency, showing that algorithmic advancements such as presolve techniques, branch-and-bound strategies, and heuristic improvements have contributed significantly to solver performance gains, often independent of hardware improvements.

To model the evolution of algorithmic efficiency, we use a logistic growth function:

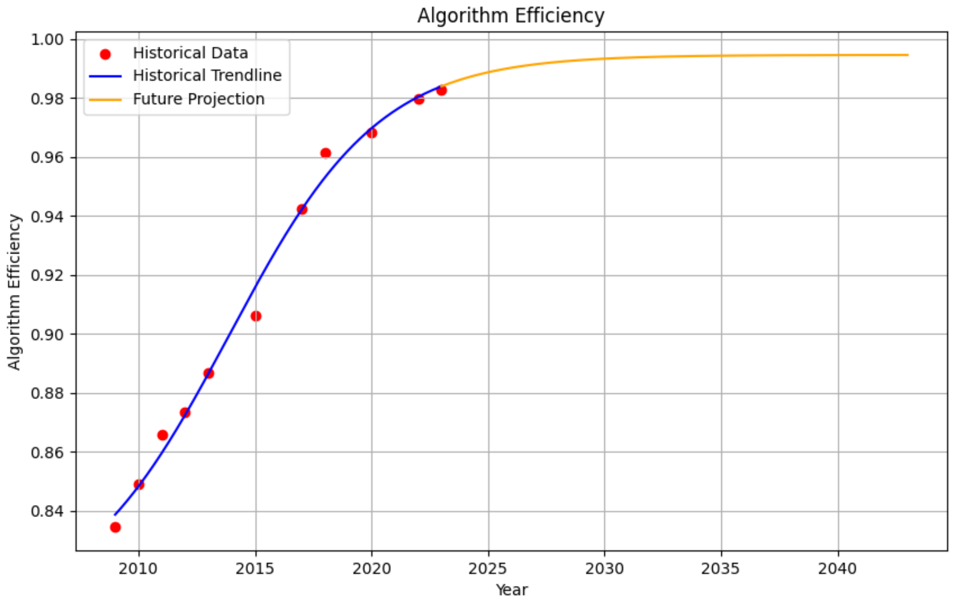

Figure 2: Projected algorithmic efficiency trends based on logistic modeling.

$$E_a(t) = E_{a0} + \frac{E_{a1}}{1 + \exp(-\alpha_a(t - \beta_a))} \tag{3}$$

where:

- E_{a0} represents the baseline algorithmic efficiency,
- E_{a1} is the asymptotic efficiency gain,
- α_a determines the rate of efficiency improvement,
- β_a is the inflection point where efficiency gains slow.

This function was chosen because algorithmic improvements follow an S-curve pattern, with rapid early advancements that eventually plateau as theoretical limits are approached.

The parameters were estimated using nonlinear curve fitting in Python, based on historical data. The results, visualized in Figure 2, confirm that algorithmic efficiency has improved significantly over time. Between 2009 and 2023, the fraction of solved problems increased from 83.45% to 98.27%. The model predicts that by 2043, solver efficiency will exceed 99.5%, suggesting that most benchmark problems will be solvable within reasonable computational limits. However, as the logistic model indicates, further improvements will slow due to fundamental algorithmic constraints.

These findings highlight the importance of continuous algorithmic innovation in maintaining computational feasibility. While hardware improvements contribute to performance gains, solver

efficiency has played a crucial role in making large-scale optimization problems solvable. Future research should explore alternative solution methods, such as reinforcement learning-based solvers, quantum optimization techniques, and hybrid AI-driven mathematical programming, to address the plateauing efficiency gains predicted in the coming decades.

2.3 Power Usage Effectiveness

PUE is a key metric used to evaluate the energy efficiency of data centers. It quantifies how efficiently a facility utilizes energy by comparing total energy consumption to the energy used solely for computational workloads. PUE is defined as:

$$PUE = \frac{\text{Total Facility Energy}}{\text{IT Equipment Energy}} \tag{4}$$

A lower PUE value indicates greater energy efficiency, as a larger proportion of total energy is directed towards computing rather than cooling, lighting, or other overhead operations. The ideal PUE is 1.0, which signifies a perfectly efficient data center where all energy is used exclusively for computational tasks.

The fundamental unit used to express PUE is a dimensionless ratio, as it represents the relative efficiency of energy allocation rather than an absolute energy measurement. We transform PUE into an efficiency metric for modeling purposes by defining:

$$E_p(t) = \frac{1}{PUE(t)} \tag{5}$$

This formulation enables direct comparisons with other efficiency metrics, allowing for a unified modeling approach across hardware, algorithmic, and infrastructure improvements.

To quantify trends in power usage effectiveness, we utilized historical PUE data from data centers over time. The dataset includes:

Table 2: Power Usage Effectiveness (PUE) of Data Centers Over Time

Year	PUE
2007	2.5
2008	2.4
2009	2.2
2010	2.0
2011	1.8
2013	1.6
2016	1.5
2018	1.6
2020	1.6
2022	1.5

These figures illustrate the steady decline in PUE over time, reflecting improvements in data center design, cooling technologies, and power management strategies. NVIDIA's research on

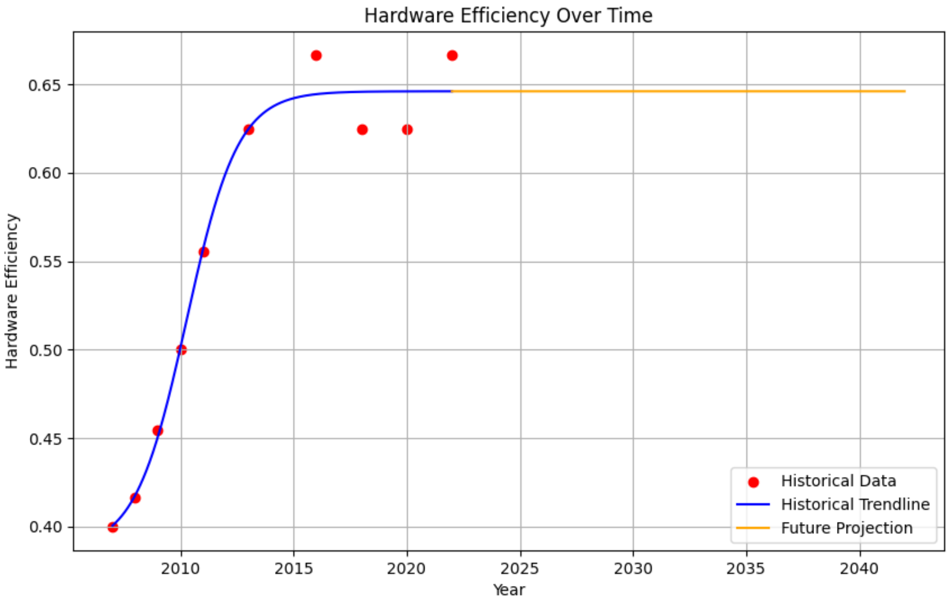

Figure 3: Projected power usage effectiveness (PUE) trends based on logistic modeling.

sustainable computing highlights innovations such as liquid cooling, dynamic power optimization, and AI-driven workload scheduling as key contributors to these gains [8].

To model the evolution of power usage effectiveness, we apply a logistic function:

$$E_p(t) = E_{p0} + \frac{E_{p1}}{1 + \exp(-\alpha_p(t - \beta_p))} \tag{6}$$

where:

- E_{p0} represents the baseline power efficiency,
- E_{p1} is the asymptotic efficiency gain,
- α_p determines the rate of efficiency improvement,
- β_p is the inflection point where gains slow.

This function was chosen because PUE trends exhibit diminishing returns: rapid early improvements due to architectural optimizations and cooling advancements, followed by a gradual plateau as theoretical limits approach.

The parameters were estimated using nonlinear curve fitting in Python, based on historical data. The implementation follows the standard methodology of fitting a logistic regression to capture the observed efficiency trend.

The results, visualized in Figure 3, confirm that power usage effectiveness has steadily improved, with PUE decreasing from 2.5 in 2007 to 1.5 in 2022. The logistic model predicts further reductions, though improvements will slow as data centers approach the theoretical minimum PUE. By 2040, projected efficiency gains indicate that most large-scale facilities will operate near 1.3, suggesting that further energy reductions will require alternative infrastructure advancements, such as direct renewable energy integration and novel cooling methodologies.

These findings highlight the critical role of infrastructure optimization in AI's long-term sustainability. While hardware and algorithmic improvements contribute significantly to efficiency gains, advancements in power usage effectiveness ensure that supporting infrastructure does not become a bottleneck for energy efficiency. Future research should explore the integration of advanced cooling methods, AI-driven data center management, and direct energy recovery systems to maintain efficiency gains beyond the expected plateau.

3. Computational Demand

3.1 Overview

Computational demand in the context of AI refers to the total amount of computing power required to train and operate AI models. As AI systems become increasingly complex and widely adopted, their computational burden grows, leading to higher energy consumption and environmental impact. This demand is driven by two main factors: (1) training, which involves the development of new models requiring extensive optimization cycles, and (2) inference, the real-time deployment of trained models for user applications. While training costs dominate initial computational requirements, inference demand scales exponentially as AI systems are deployed globally [1, 2].

To quantify computational demand, we use floating-point operations (FLOPs) as the fundamental unit, measuring both training and inference workloads. FLOPs provide a hardware-agnostic metric for computational intensity, allowing comparisons across architectures and AI paradigms. For large-scale assessment, we aggregate demand in FLOPs per year (FLOP/s-year) to capture the sustained growth of AI's energy demand over time. Unlike alternative measures such as GPU-hours or electricity consumption, FLOPs offer a direct measure of computational effort independent of energy efficiency variations.

Existing studies have approached computational demand from different perspectives. Strubell et al. [1] analyzed the energy cost of training deep learning models, estimating the carbon footprint based on GPU-hours. Patterson et al. [2] extended this approach by comparing energy usage across various hardware accelerators, showing that AI-optimized chips like TPUs significantly reduce power consumption. OpenAI [9] documented the exponential growth of AI compute demand, noting that computational requirements have doubled approximately every 3.4 months since 2012. More recently, studies have highlighted that inference is overtaking training as the dominant driver of AI energy consumption, particularly in commercial applications where millions of real-time queries are processed daily [10].

We improve upon these methodologies by developing a dynamic computational demand model

that separately accounts for both training and inference, recognizing their distinct contributions to AI's overall energy demand. Rather than treating computational demand as a single, uniform trend, our approach distinguishes between the increasing complexity of AI models requiring extensive training and the rapidly growing inference workloads driven by widespread AI adoption. By modeling these factors independently, we can better assess how improvements in hardware, algorithms, and data center efficiency influence long-term AI sustainability. This approach provides a more nuanced understanding of how computational demand evolves and offers insights into strategies for mitigating its environmental impact.

3.2 Training Demand

Training demand refers to the computational resources required to develop AI models, encompassing the full lifecycle of model training, hyperparameter tuning, and retraining. Unlike inference demand, which scales with user adoption, training demand is concentrated in discrete phases where models undergo optimization using vast datasets and iterative gradient updates. The unique characteristic of training is its high computational intensity, often requiring large-scale parallel processing on specialized hardware such as GPUs and TPUs. Training efficiency is influenced by several key factors, including model complexity, dataset size, training frequency, and optimization techniques. In the following sections, we break down these components to analyze their contributions to overall AI compute growth and identify trends that could mitigate training's energy impact.

3.2.1 Model Complexity

Model complexity refers to the computational burden associated with training deep learning models, primarily determined by the number of parameters and the total floating-point operations (FLOPs) required for training. As AI models grow in scale, their increasing complexity directly impacts computational demand, energy consumption, and hardware requirements. Larger models require extensive optimization cycles and high-memory architectures, making model complexity a fundamental driver of AI's long-term sustainability.

The fundamental unit used to quantify model complexity in this study is floating-point operations (FLOPs) per training run. This unit provides a direct and hardware-agnostic measure of computational intensity, allowing comparisons across different architectures and scaling trends. Complexity is expressed on a per-year basis, aggregating trends over time to estimate how model growth influences AI's overall energy demand.

To analyze historical trends in model complexity, we aggregated data from multiple large-scale AI models, using LifeArchitect.ai's Models Table, curated by Dr. Alan D. Thompson [11]. The dataset includes the following key milestones:

These figures illustrate the rapid growth in model complexity over time, highlighting the transition from early deep learning architectures to billion-parameter-scale transformer models. As AI research progresses, model complexity has followed a power-law scaling trend, with FLOP requirements increasing exponentially.

To model the evolution of complexity, we apply a modified power-law function:

Table 3: Estimated Model Complexity Over Time (FLOPs per Training Run)

Year	Model Complexity (FLOPs)
2018	0.000702
2019	14,890
2020	153,000
2022	424,000
2023	138,000,000
2024	386,000,000

$$C_c(t) = C_{c0}(t + \beta)^\alpha \tag{7}$$

where:

- $C_c(t)$ represents the estimated model complexity at time t,
- C_{c0} is the initial complexity baseline,
- α represents the growth exponent, capturing the rate of complexity increase,
- β shifts the timeline to better fit observed trends.

This model was chosen because empirical evidence suggests AI model complexity has scaled super-linearly, with larger models requiring disproportionately more computational resources. By incorporating a power-law function, we align with prior research on scaling laws, such as Kaplan et al. (2020) and Sevilla et al. (2022), which demonstrate that AI performance improves predictably with increased computational investment [9].

The parameters were estimated using nonlinear curve fitting in Python, based on the dataset from LifeArchitect.ai. The power-law regression successfully captured both historical trends and future projections, aligning closely with observed scaling patterns.

The results confirm that model complexity has increased dramatically over the past five years, with computational requirements growing by multiple orders of magnitude. If current trends continue, the model predicts that by 2045, state-of-the-art AI models will require FLOPs in the range of 10^{11} to 10^{12} per training run. However, this scaling is not sustainable indefinitely, as it is constrained by hardware limitations, energy costs, and diminishing returns in model performance.

These findings highlight the importance of efficiency improvements in both hardware and algorithms. While larger models tend to perform better, alternative strategies such as sparse architectures, retrieval-augmented generation, and model compression could mitigate the unsustainable growth of FLOPs while maintaining performance. Understanding the trajectory of model complexity is crucial for developing energy-efficient AI systems that balance computational demand with practical sustainability constraints.

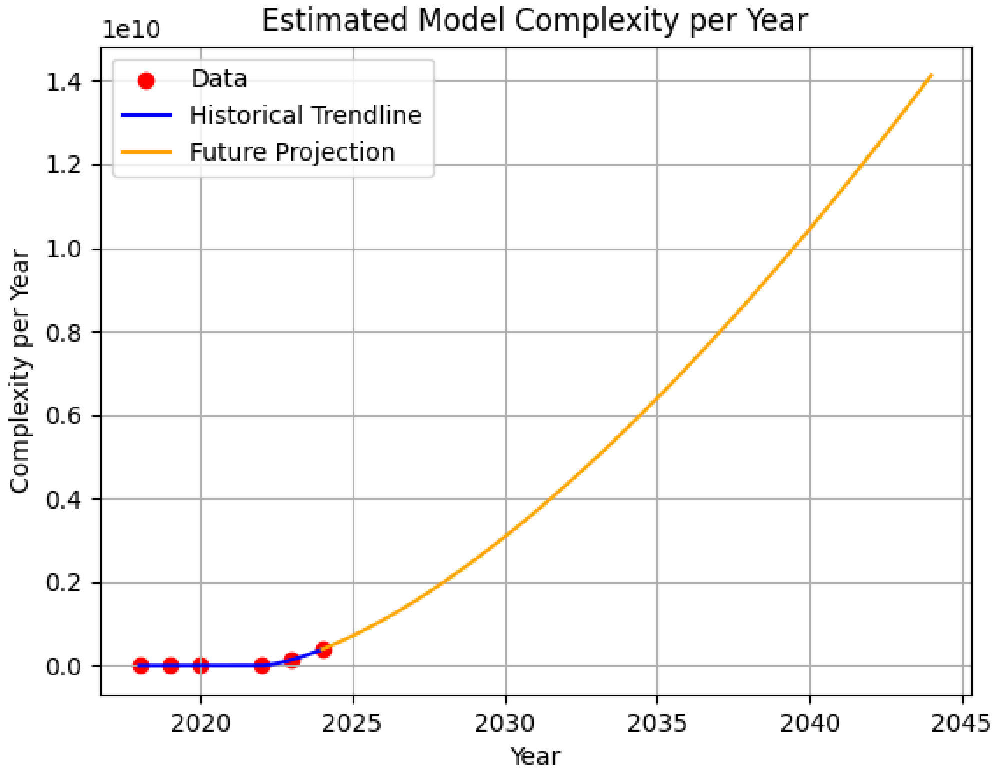

Figure 4: Projected Model Complexity Trends Based on Power-Law Growth.

3.2.2 Model Demand

Models per year refers to the number of large-scale AI models trained annually, reflecting the increasing frequency at which new models are developed and deployed. Unlike model complexity, which captures the computational intensity of individual models, this metric tracks the aggregate expansion of AI research and production models. As AI adoption grows, the number of models being trained each year is expected to rise, influencing overall computational demand and energy consumption. Investigating models per year in conjunction with model complexity provides a more complete picture of AI's energy demand, as both factors contribute to the growing strain on computational infrastructure. While increasing complexity drives up per-model training costs, the rising number of models compounds the overall computational burden, making it essential to examine both trends together.

The fundamental unit used in this study is the number of models trained per year. This unit was chosen because it provides a direct measure of AI research and deployment trends. Unlike FLOPs, which quantify per-model training costs, tracking the number of models per year allows us to estimate the cumulative computational load on AI infrastructure. This distinction is crucial for understanding how AI's energy demand scales over time.

To analyze historical trends, we aggregated data from LifeArchitect.ai's Models Table, compiled by Dr. Alan D. Thompson [11]. The dataset provides model release information from 2018 onward, covering a range of architectures and scaling behaviors. The following table presents key data points:

Table 4: Estimated Number of AI Models Trained Per Year

Year	Models Trained
2018	2.25
2019	4.00
2020	3.43
2021	1.99
2022	15.70
2023	88.95
2024	183.49

These figures illustrate a rapid increase in the number of large-scale AI models trained each year. Early AI development focused on a small number of high-profile models, but recent years have seen an explosion in AI model production. This trend is expected to continue as computational resources become more accessible and organizations train multiple versions of models for different applications. Examining this trend alongside model complexity allows for a more comprehensive understanding of AI's future energy requirements. Even if individual models become more efficient, a rising number of models could still lead to increased overall demand.

To model the evolution of models per year, we apply a modified power-law function:

$$C_m(t) = C_{m0}(t + \lambda_m)^{\eta_m} \tag{8}$$

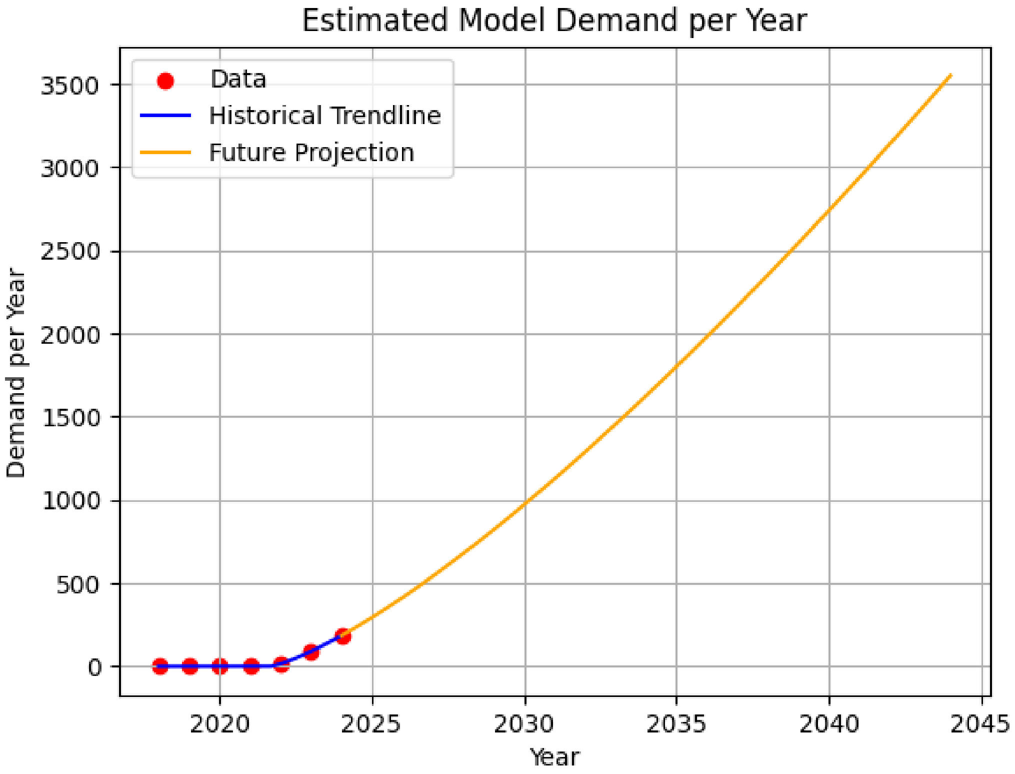

Figure 5: Projected Number of AI Models Trained Per Year Based on Power-Law Growth.

where:

- $C_m(t)$ represents the number of models trained at time t,
- C_{m0} is the initial baseline count of models per year,
- η_m represents the growth exponent, capturing the rate of increase,
- λ_m shifts the timeline to better fit observed trends.

This model was chosen because empirical evidence suggests that AI research has followed an accelerating trajectory, with model production rates increasing non-linearly. Unlike fixed-interval model release cycles seen in earlier AI research, contemporary trends show that multiple models are now trained and fine-tuned continuously. The power-law function captures this behavior while allowing for future projections based on observed data.

The parameters were estimated using nonlinear curve fitting in Python, based on historical trends from LifeArchitect.ai. The fitted function successfully replicates the observed growth trend, capturing both past fluctuations and expected future trajectories.

The results confirm that the number of AI models trained per year has grown significantly, with a sharp increase observed in 2022 and beyond. If current trends continue, the model predicts that

by 2045, thousands of large-scale AI models will be trained annually. However, this expansion raises concerns about energy consumption, sustainability, and computational feasibility. Without improvements in efficiency, the growing number of AI models could place unprecedented demand on global computing infrastructure. Investigating the growth of model production alongside model complexity highlights the need for systemic improvements in AI efficiency, ranging from hardware acceleration to algorithmic optimizations.

These findings highlight the importance of optimizing training efficiency and model reusability. Strategies such as transfer learning, fine-tuning, and modular AI architectures could mitigate the exponential growth in training costs while maintaining innovation. Understanding the trajectory of models per year is essential for anticipating the future impact of AI on energy consumption and computational demand.

3.3 Usage Demand

Usage demand refers to the computational burden of deploying trained AI models for real-world applications, handling continuous user queries across various domains such as search engines, recommendation systems, autonomous vehicles, and language models. Unlike training, which is a one-time cost per model, inference demand grows proportionally to user adoption and usage frequency, often becoming the dominant driver of AI's long-term energy consumption. Key factors shaping inference demand include model architecture, batch processing efficiency, query volume, and hardware deployment strategies. In the subsequent sections, we analyze how these elements influence AI's computational footprint and explore potential optimizations to improve inference efficiency while sustaining performance.

3.3.1 Country Groupings by Development Level

To analyze the disparity in AI adoption and its environmental impact, countries were categorized into five distinct groups based on their level of development. This classification is essential for understanding the differential adoption of AI technologies, variations in energy infrastructure, and regional disparities in carbon emissions. The grouping is based on each country's quintile rank on the Inequality-Adjusted Human Development Index (IHDI), which accounts for disparities in health, education, and income [12].

Group A (Most Developed): Includes the United States, Canada, Western European nations, Japan, Australia, and South Korea. These countries have the highest levels of AI research, widespread access to high-performance computing, and well-established green energy policies. The energy efficiency of AI in these nations is significantly higher due to advanced hardware and optimized data center management [14]. Their AI adoption is driven by large-scale corporate investments and government-backed initiatives in AI safety and regulation.

Group B: Consists of emerging economies with strong technological bases, such as China, Eastern European nations, and select Latin American countries like Brazil. These countries have rapidly expanding AI sectors but often rely on more carbon-intensive energy grids. While their

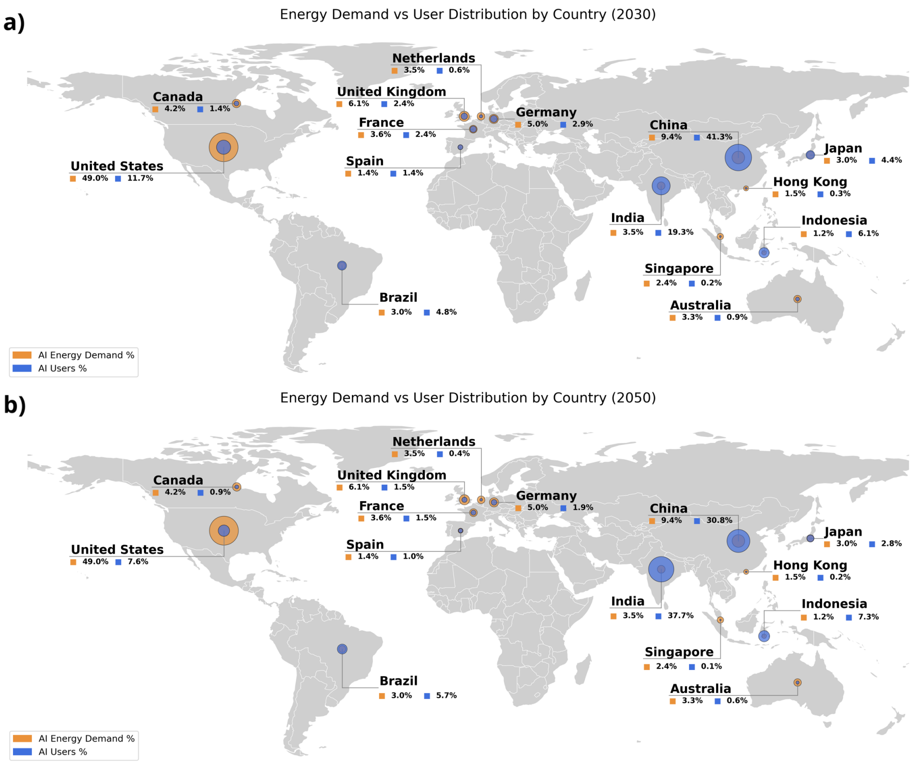

Figure 6: **Global distribution of AI user shares and corresponding energy burdens across selected countries for 2030 and 2050.** Panels (a) and (b) depict each country's share of global energy demand of AI in yellow among the countries analyzed for 2030 and 2050 respectively. In all panels, each country's share of global AI users is shown in blue, with the size of each bubble corresponding to a larger share. Made with Natural Earth.

AI adoption is increasing, the efficiency of AI computation in these nations is lower due to older infrastructure and a reliance on coal and gas power [15].

Group C: Includes middle-income countries with moderate AI adoption, such as Mexico, Egypt, Philippines, and Indonesia. These countries have growing AI sectors but lack widespread computational resources. They often depend on foreign investments and imported semiconductor technologies, which introduce additional energy and transportation costs. Their AI adoption follows a slower trajectory, with usage being more application-specific rather than research-driven [16].

Group D: Consists of lower-middle-income nations in Southern Africa and South Asia. These countries have limited AI infrastructure, minimal domestic research, and often rely on AI services provided by multinational firms. Energy inefficiencies are prevalent due to reliance on outdated power grids, leading to higher per-operation energy costs in AI computations [17].

Group E (Least Developed): Comprises countries with minimal AI presence, including regions in Sub-Saharan Africa, war-affected nations, and those with severe energy access issues. AI development is virtually nonexistent, and these nations are likely to remain dependent on foreign

AI services if infrastructural challenges are not addressed. These countries contribute negligibly to AI-driven carbon emissions but are disproportionately affected by the consequences of climate change, making sustainable AI solutions particularly relevant for them [18].

The grouping structure aligns with existing economic and developmental classifications, which categorize countries based on their economic output, technological readiness, and overall human development. Previous studies have established strong correlations between a country's IHDI ranking and its level of digital infrastructure, energy efficiency, and AI adoption potential [12]. Countries in the highest development tier (Group A) tend to have well-established AI research ecosystems, robust semiconductor industries, and highly efficient energy grids, while those in the lowest tiers (Group D and E) often struggle with infrastructure gaps and limited computational capacity [13].

3.3.2 Population Growth

Since AI energy consumption is directly proportional to the number of user queries, population growth must be incorporated into the broader energy model. Population growth is modeled as an exponential function, capturing the demographic trends in each country group. This formulation is consistent with empirical population data and is widely used in demographic modeling due to its ability to represent both stable and rapidly growing populations. The population at any given time t is expressed as:

$$P_i(t) = P_{i0} \cdot e^{r_i t} \tag{9}$$

where:

- $P_i(t)$ is the population of user group i at time t,
- P_{i0} is the initial population of user group i,
- r_i is the population growth rate in group i.

This exponential function aligns well with historical population trends across country groups, as shown in Figure 7. The estimated parameters P_{i0} and r_i were derived through curve fitting applied to World Bank population data [19]. The exponential model enables accurate projections for each region's population, ensuring that variations in demographic expansion are correctly incorporated into our broader AI demand and energy consumption framework.

The necessity of measuring population separately for each country group arises from distinct demographic trends and AI infrastructure disparities. In highly developed nations (Group A), population growth is slow or even declining, meaning AI demand will primarily increase due to higher per capita usage rather than demographic expansion. In contrast, rapidly growing regions (Groups D and E) will see a rise in both absolute AI users and infrastructure demand, leading to a dual effect on total AI-driven energy consumption.

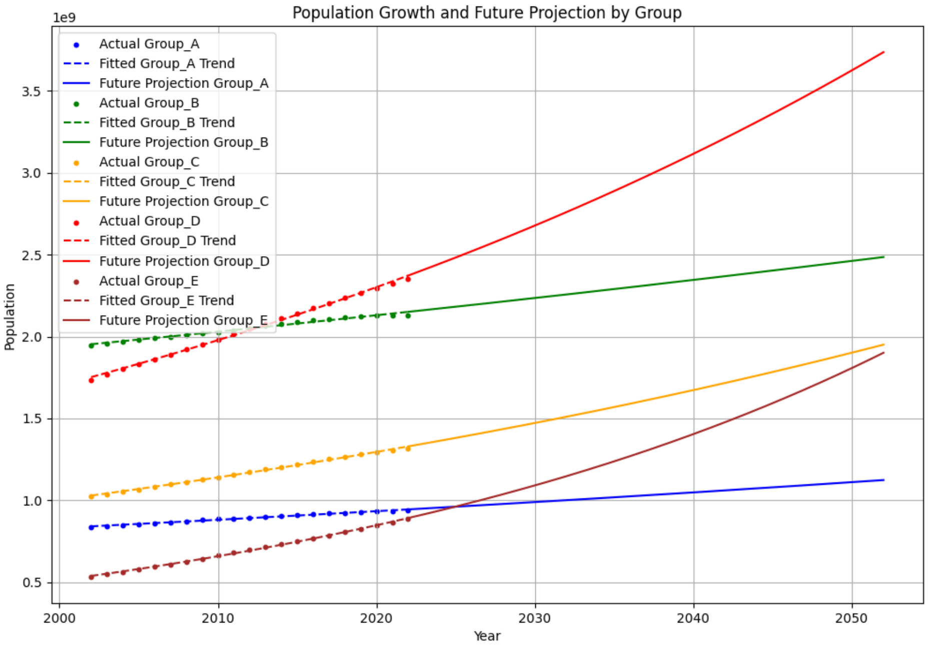

Figure 7: Projected Population Growth by Country Group Based on World Bank Data. The trends indicate that population expansion is concentrated in Groups C, D, and E, which could significantly influence future AI demand and energy consumption.

By separating population growth per group, we ensure that our model accurately reflects AI expansion in different regions. If population were modeled as a single global entity, we would risk overestimating demand in regions with declining or stable populations and underestimating the impact of rapidly growing user bases in developing countries.

3.3.3 Adoption Rate

The adoption rate of AI, denoted as $A_i(t)$, follows a logistic function, which is widely used to describe the diffusion of new technologies. The logistic function captures the characteristic S-shaped curve, where adoption begins slowly, accelerates during widespread acceptance, and finally plateaus as the technology reaches saturation. We define the adoption function for each country group as:

$$A_i(t) = \frac{1}{1 + \exp\left(-\gamma_i (t - t_{0i})\right)} \tag{10}$$

where:

- $A_i(t)$ is the adoption rate in region i at time t,
- γ_i is the growth rate parameter for region i, determining how quickly adoption occurs,
- t_{0i} is the midpoint of the adoption curve, representing the time at which the adoption rate reaches 50%.

The parameters γ_i and t_{0i} are calibrated based on historical adoption data for the internet, sourced from World Bank records [13]. This approach is justified as AI services rely on digital connectivity, making internet adoption the most relevant precedent for estimating AI adoption timelines. We used the time it took for internet adoption to reach 20% and 80% for Group A as a reference and adjusted the approach for other groups based on observed disparities in technological diffusion.

To reflect real-world disparities in technology adoption, different time intervals were used for estimating the logistic curve parameters:

Group A: Developed Economies

For Group A, which consists of the most developed nations, historical internet adoption data shows a relatively fast transition. The time to move from 20% adoption to 80% adoption was set at 10 years ($At20, At80 = 5, 15$). This informed the logistic growth rate:

$$\gamma_A = \frac{2\ln(4)}{At20 - At80} \tag{11}$$

The midpoint t_{0A} was chosen as:

$$t_{0A} = \frac{At20 + At80}{2} \tag{12}$$

This setting captures the rapid technological diffusion in developed economies, where high infrastructure availability and digital literacy enable quick adoption.

Group B: Emerging Economies

For Group B, representing high-middle-income countries, the adoption lag is slightly longer due to slower infrastructure expansion. The transition time was set at 13 years ($Bt20, Bt80 = 8, 21$), leading to:

$$\gamma_B = \frac{2\ln(4)}{Bt20 - Bt80}, \quad t_{0B} = \frac{Bt20 + Bt80}{2} \tag{13}$$

This adjustment ensures a more gradual adoption curve, reflecting the time required to develop necessary AI-supporting infrastructure.

Group C: Lower Middle-Income Countries

Group C exhibits more fragmented technology adoption, often constrained by inconsistent internet penetration rates. Instead of the 20%-80% range, we calibrated adoption based on the 10%-40% range ($Ct10, Ct40 = 6, 19$):

$$\gamma_C = \frac{-\ln(6)}{Ct40 - Ct10} \tag{14}$$

$$t_{0C} = Ct10 + (Ct40 - Ct10)\frac{\ln(9)}{\ln(6)} \tag{15}$$

This formulation accounts for slower diffusion, especially in rural areas where infrastructure remains underdeveloped.

Group D: Developing Nations

For Group D, we adopted a similar approach but with even longer adoption delays. The range was set at 10%-40% ($Dt10, Dt40 = 9, 20$), given historical trends:

$$\gamma_D = \frac{-\ln(6)}{Dt40 - Dt10}, \quad t_{0D} = Dt10 + (Dt40 - Dt10)\frac{\ln(9)}{\ln(6)} \tag{16}$$

This adjustment reflects the structural barriers to AI adoption, such as limited access to computing resources and delayed policy implementation.

Group E: Least Developed Nations

For Group E, AI adoption is expected to be particularly slow, mirroring internet diffusion in these regions. Since many Group E nations have yet to reach 80% internet adoption, we used a 5%-20% range ($Et5, Et20 = 13, 36$):

$$\gamma_E = \frac{-\ln(6)}{Et20 - Et5} \tag{17}$$

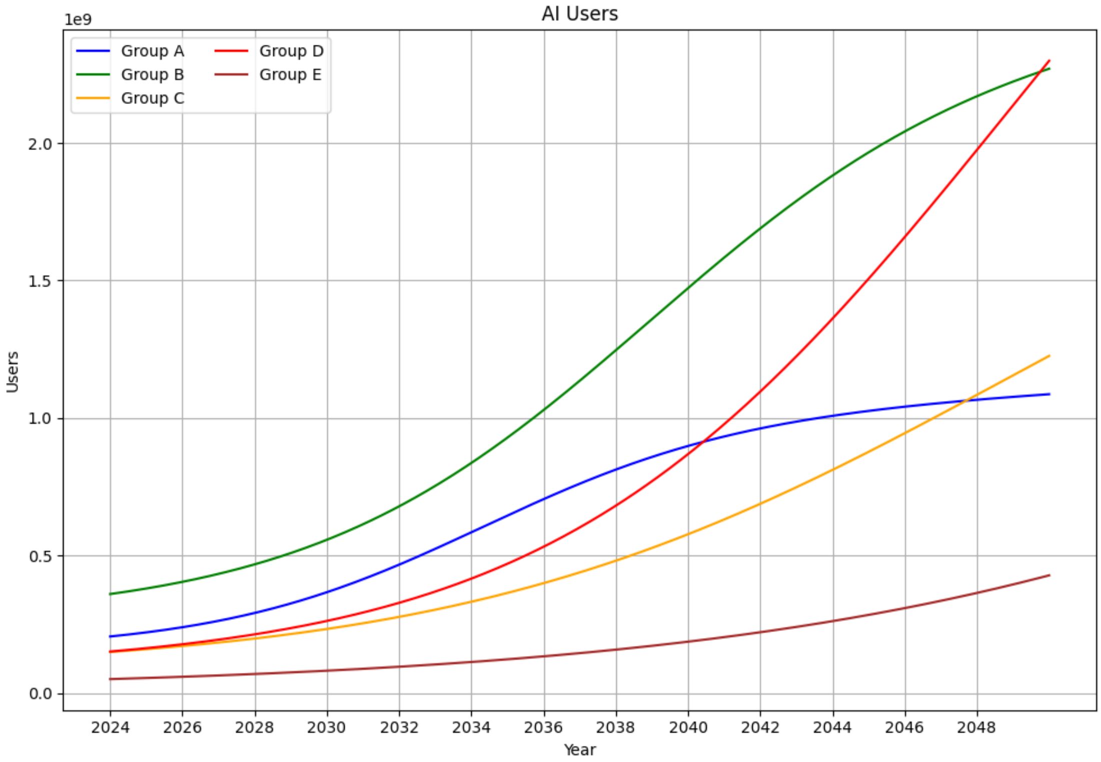

Figure 8: Projected AI User Growth by Country Group. The trends reflect differences in both population size and adoption speed, leading to stark disparities in AI user expansion.

$$t_{0E} = Et5 + (Et20 - Et5)\frac{\ln(9)}{\ln(6)} \tag{18}$$

This captures the structural challenges that slow AI adoption in Group E, including inadequate digital infrastructure, low literacy rates, and economic constraints.

Figure 8 and Figure 9 illustrate AI adoption trends by country group. The first figure shows the projected number of AI users, derived as:

$$U_i(t) = P_i(t) \cdot A_i(t) \tag{19}$$

where $P_i(t)$ represents the population projections derived earlier. The second figure presents the corresponding adoption rates, showing the expected trajectory of AI diffusion.

3.4 Query Complexity

Query complexity quantifies the computational burden of executing AI inference tasks, measuring the expected number of operations required to process a given query. Unlike model complexity, which reflects the size and depth of neural networks during training, query complexity determines

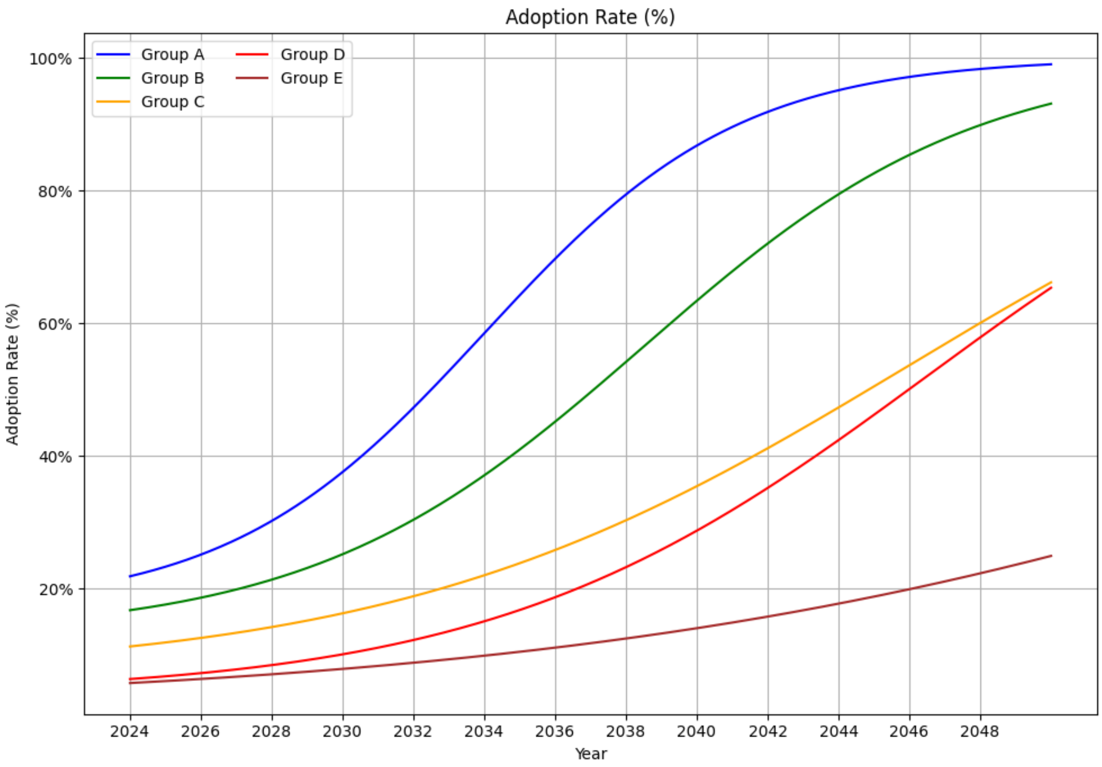

Figure 9: Projected AI Adoption Rates Across Country Groups. The logistic adoption model captures the expected diffusion trajectory based on historical internet adoption patterns.

the real-time computational cost of AI usage. This distinction is critical because inference workloads are projected to dominate AI-related energy consumption in the long term.

Query complexity is expressed in terms of floating-point operations (FLOPs) per query. This unit aligns with hardware efficiency metrics, enabling a direct comparison between computational demand and available processing power. Using FLOPs allows for a consistent framework to estimate energy consumption, as FLOPs-to-Watt ratios are commonly reported for AI accelerators.

Rather than using raw token counts, we derive query complexity from model parameters. This choice is motivated by empirical observations that larger models exhibit superlinear increases in per-query computational cost. Specifically, transformer-based architectures scale with at least $O(n^2)$ complexity in attention operations, making model size a robust proxy for query cost. Using token-based scaling would introduce significant variance across architectures, whereas parameter scaling provides a more generalizable approach.

Table 5 presents historical query complexity estimates for AI models, derived from the number of model parameters rather than processed tokens. This aggregation is sourced from [11].

**Year**	**Query Complexity (FLOPs)**
2020	5.71
2021	7.36
2022	86.98
2023	525.21
2024	1238.19

Table 5: Estimated Query Complexity Over Time

We model query complexity growth using a power-law function:

$$C_q(t) = C_{q0}(t - t_0 + \beta)^{\alpha} \tag{20}$$

where:

- $C_q(t)$ represents query complexity at time t,
- C_{q0} is the initial complexity coefficient,
- α determines the growth exponent, capturing superlinear scaling,
- β adjusts for shifts in the historical trend.

A power-law model is chosen because AI models have exhibited exponential parameter growth, leading to polynomial increases in per-query costs. The parameters are estimated via curve fitting using historical complexity data.

Figure 10 illustrates the historical trend and projected future complexity. The power-law model successfully captures the rapid escalation in query complexity, projecting an order-of-magnitude

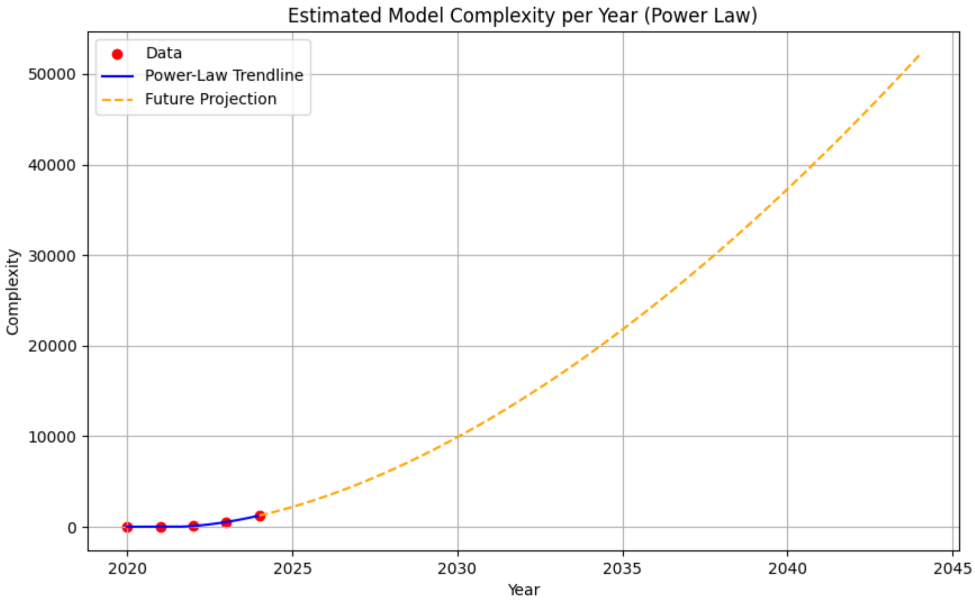

Figure 10: Projected Query Complexity Growth Based on Power-Law Model

increase in the next decade.

The implications of this trend are significant. If computational efficiency improvements do not outpace query complexity growth, AI inference workloads will become increasingly energy-intensive. This highlights the need for advances in hardware acceleration and algorithmic optimizations to mitigate energy demand.

4. Total Power

4.1 Historical Trends in Global Power Consumption

To understand the energy demand of AI, we first establish the historical trajectory of total global electricity consumption. This dataset spans from 1990 to 2023, with power consumption values measured in terawatt-hours (TWh) per year. The data is fitted using an exponential growth model:

$$P_{\text{total}}(t) = P_0 e^{\epsilon t} \tag{21}$$

where:

- $P_{\text{total}}(t)$ is the total electricity consumption at year t,
- P_0 is the base power consumption in 1990,
- ϵ is the estimated growth rate of global energy consumption.

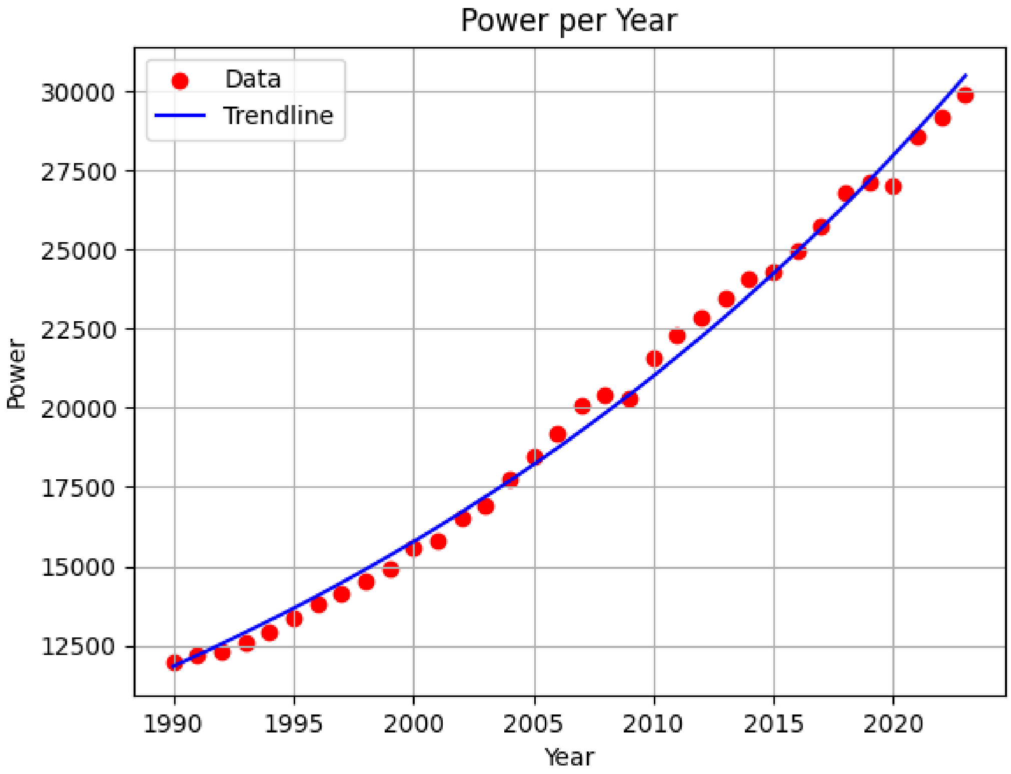

Figure 11: Global Power Consumption Over Time with Fitted Exponential Trend

The fitted model in Figure 11 demonstrates a long-term upward trend in global electricity demand, primarily driven by industrialization, the expansion of digital infrastructure, and population growth.

4.1.1 Comprehensive Formula used in this Study

Total AI power demand is computed as:

$$P(t) = \frac{C_t(t)}{E_p(t)E_h(t)/E(t)} \tag{22}$$

where:

- $C_t(t) = C_c(t)C_m(t) + C_q(t)C_u(t)$ is the total computational demand,
- $E_p(t)$ is power usage effectiveness (PUE), accounting for infrastructure overheads,
- $E_h(t)$ represents hardware efficiency improvements,
- $E_a(t)$ represents algorithmic efficiency improvements.

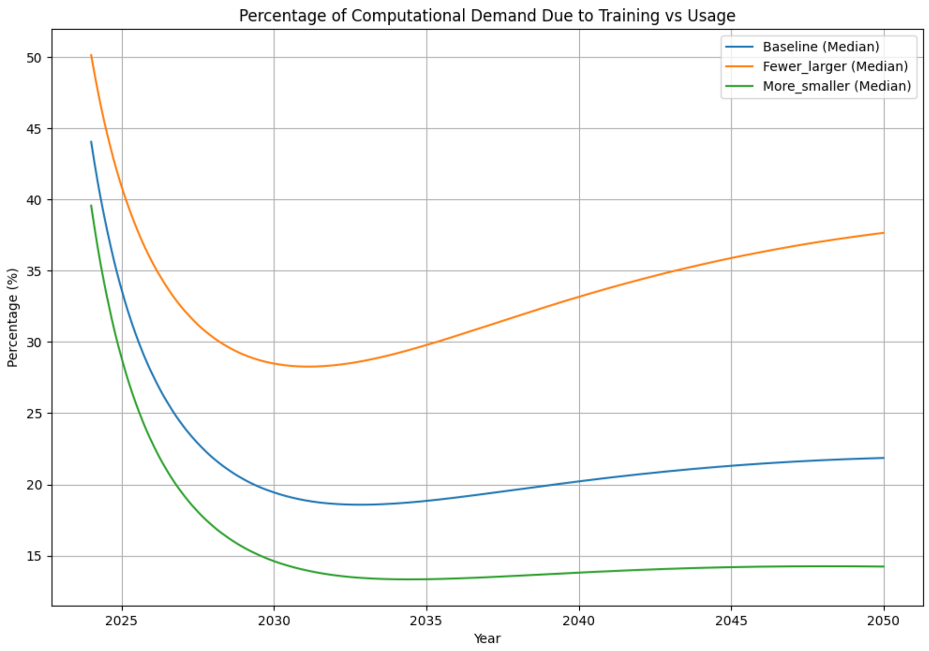

Figure 12: Projected AI Power Consumption, Decomposed into Training and Inference

4.1.2 Breakdown of AI Energy Usage

To distinguish between training and inference, we decompose total AI power consumption:

$$P_t(t) = \frac{C_c(t)C_m(t)}{E_p(t)E_h(t)/E_a(t)} \tag{23}$$

The results in Figure 12 indicate that while training is currently the dominant energy driver, inference power demand is expected to surpass it as AI becomes more widely adopted. This shift is driven by increasing user adoption, higher query complexity, and improvements in computational efficiency that favor inference workloads over large-scale model training.

4.2 Scenarios

4.2.1 Baseline

This scenario represents a continuation of current AI development trends, with steady growth in model complexity and deployment.

Implementation in Code:

- The scenario multipliers are set to $(1, 1)$, meaning computational complexity (C_c) and model demand (C_m) follow empirical trends without modification.

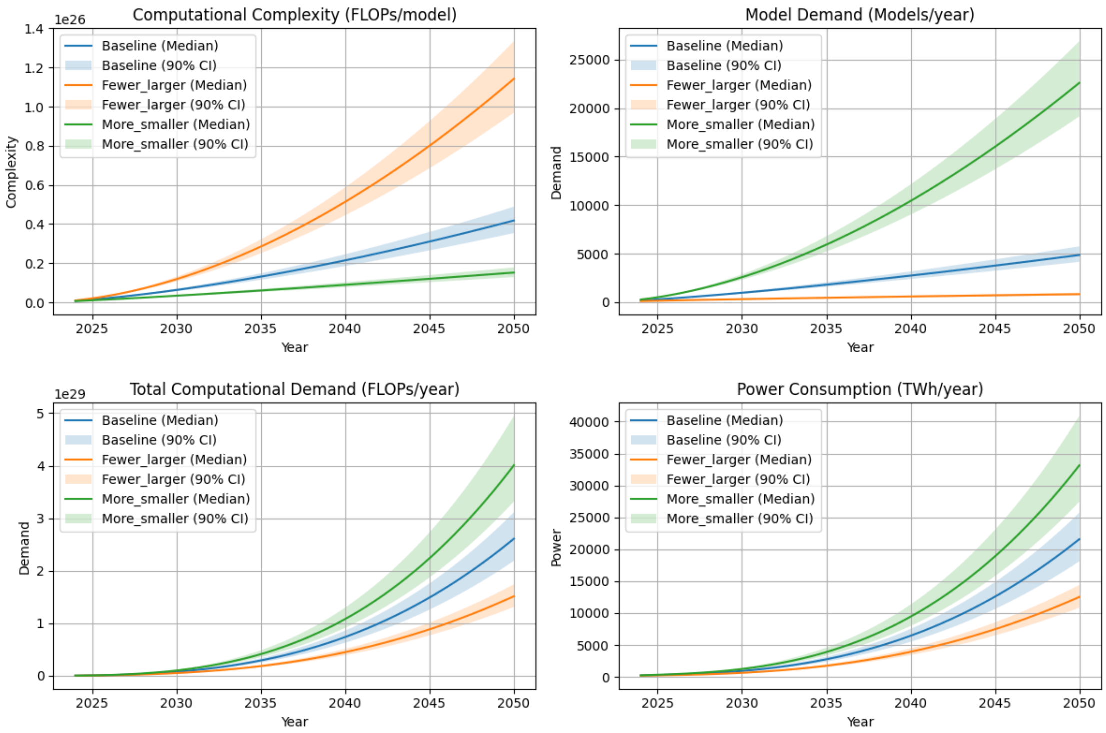

Figure 13: Energy Demand of AI by Scenario

- AI's computational demand (C_t) grows based on established extrapolations.
- Efficiency gains in hardware (E_h), algorithm optimization (E_a), and infrastructure (E_p) reduce per-FLOP energy consumption.
- AI adoption follows a logistic function across different population groups.

Implications:

- AI's share of global energy consumption **doubles by the early 2030s** and reaches **approximately 30% by 2050**.
- Computational demand escalates due to model complexity and user growth, **offsetting efficiency gains**.
- CO_2 emissions **increase 16.6x** relative to 2030 levels.

4.2.2 Fewer, Larger Models

AI models consolidate into fewer but more computationally intensive systems, requiring significantly more FLOPs per model but being trained less frequently.

Implementation in Code:

- **Complexity Multiplier** $= 1.2$ (each model requires more FLOPs for training).

- **Model Demand Multiplier** $= 0.6$ (fewer models are trained per year).
- This increases **training energy demand** but reduces **usage energy demand** ($C_q C_u$).

Implications:

- Reduces inference-related energy demand, leading to a lower total AI energy demand compared to baseline.
- **Peak power consumption during training increases**, straining localized energy grids.
- AI energy demand remains substantial, but it is concentrated in large-scale data centers.
- Lower CO_2 emissions than the baseline due to reduced AI adoption rates among consumers.

4.2.3 More, Smaller Models

AI becomes more decentralized, with a larger number of smaller models deployed, increasing inference-related energy demand.

Implementation in Code:

- **Complexity Multiplier** $= 0.8$ (models require fewer FLOPs per training cycle).
- **Model Demand Multiplier** $= 1.35$ (higher number of models are deployed per year).
- Computational demand is **driven by user adoption** ($C_u C_q$).

Implications:

- This scenario leads to the **highest total energy demand**, as AI adoption becomes widespread across industries and regions.
- **Training energy demand per model is lower**, but the **sheer number of models trained** leads to an exponential increase in energy consumption.
- **Greater strain on global energy infrastructure**, particularly in emerging markets.
- **CO_2 emissions surpass the baseline**, peaking at **22 GT by 2050** (compared to 13.25 GT in the baseline).

4.3 AI Energy Share of Total Global Energy

To assess AI's footprint within global energy demand, we compute:

$$\text{AI Energy Share}(t) = \frac{P(t)}{P_{\text{total}}(t)} \tag{24}$$

Under baseline assumptions, AI energy demand is projected to grow from a negligible share today to a significant fraction of global power consumption by mid-century.

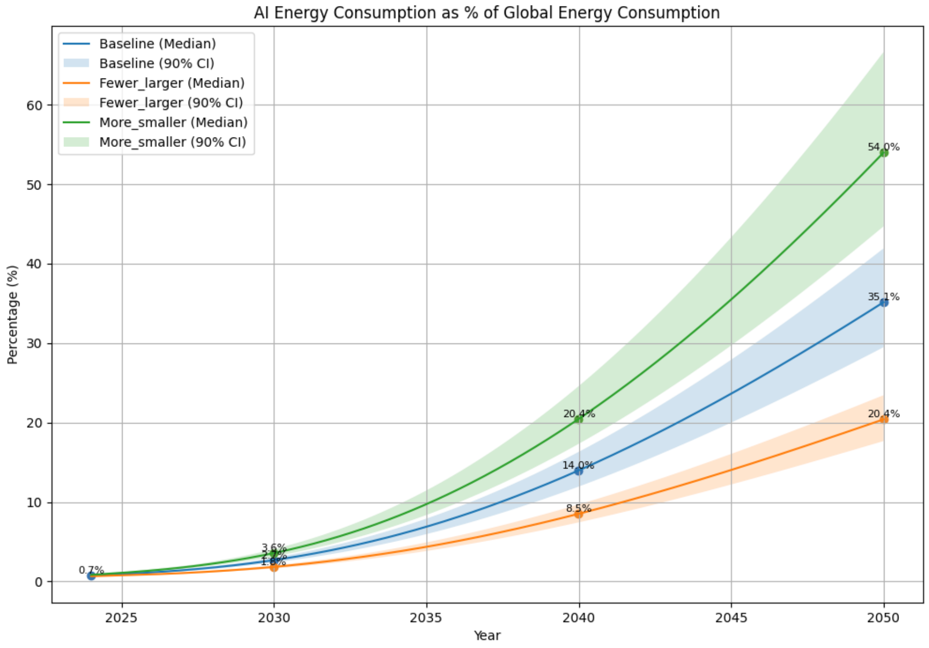

Figure 14: Projected AI Energy Consumption as a Percentage of Global Energy Use

5. Overview: Carbon Footprint of AI

6. Supply Chain Carbon Footprint

6.1 Manufacturing

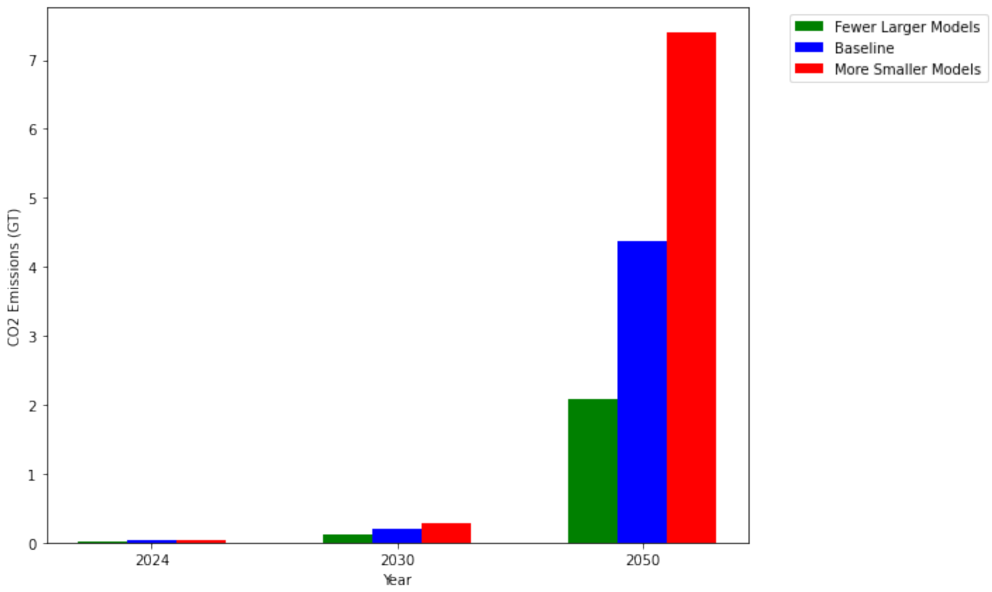

Figure 15: CO_2 Emission from Manufacturing

Year	Baseline (GT)	Fewer Larger Models (GT)	More Smaller Models (GT)
2024	0.0327	0.0258	0.0393
2030	0.1960	0.1200	0.2760
2050	4.3700	2.0800	7.3900

Table 6: CO_2 Emissions (GT) from Manufacturing by Year and Scenario

The results presented in Table 6 illustrate the projected CO_2 emissions from manufacturing in Taiwan under three distinct scenarios: Baseline, Fewer Larger Models, and More Smaller Models. In the Baseline scenario, CO_2 emissions are expected to rise from 0.0327 gigatons (GT) in 2024 to 4.37 GT by 2050, reflecting a significant increase in manufacturing activity and associated emissions over the next three decades. The Fewer Larger Models scenario, which assumes a shift toward larger, more efficient manufacturing units, projects a more moderate growth in emissions, reaching 2.08 GT by 2050. This represents a 52% reduction compared to the Baseline scenario, highlighting the potential benefits of economies of scale and improved efficiency in larger manufacturing operations. In contrast, the More Smaller Models scenario, which envisions a proliferation of smaller, less efficient manufacturing units, predicts a dramatic escalation in emissions, reaching 7.39 GT by 2050, 69% increase over the Baseline. These findings underscore the critical importance of strategic planning in manufacturing scale and efficiency to mitigate CO_2 emissions. The divergence in outcomes between the scenarios emphasizes the need for policies that incentivize the adoption of larger, more efficient models to achieve substantial emission reductions and align with global climate goals. The cost per GPU was obtained from *HPCwire* [29] and is subsequently used in conjunction with GPU demand figures derived from the environmentally extended multi-regional input-output (EE-MRIO) model (Section 7.2) .

6.2 Transportation

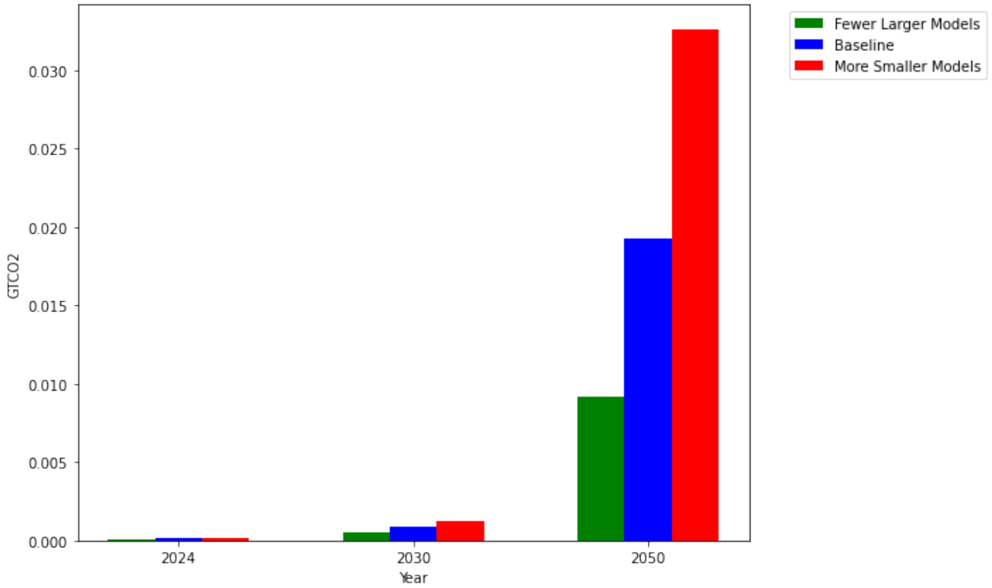

Figure 16: CO_2 Emission from Transportation

Year	Baseline (GT)	Fewer Larger Models (GT)	More Smaller Models (GT)
2024	0.000144	0.000114	0.000173
2030	0.000862	0.000528	0.001217
2050	0.019271	0.009154	0.032567

Table 7: Transportation CO_2 Emissions (GT) by Year and Scenario

The projected CO_2 emissions from transportation under three scenarios—Baseline, Fewer Larger Models, and More Smaller Models—are presented in Table 7 and visualized in Figure 16. In the Baseline scenario, emissions are estimated to increase from 0.000144 gigatons (GT) in 2024 to 0.019271 GT by 2050, reflecting a steady rise in transportation activity. The Fewer Larger Models scenario, which assumes a transition to larger, more efficient transportation systems, projects a 52.5% reduction in emissions by 2050 (0.009154 GT) compared to the Baseline. Conversely, the More Smaller Models scenario, characterized by a proliferation of smaller, less efficient systems, predicts a 69% increase in emissions by 2050 (0.032567 GT) relative to the Baseline. These results highlight the significant impact of system scale and efficiency on transportation-related emissions. Furthermore, Figure 17 illustrates the growing demand for GPUs, which is closely tied to advancements in transportation technologies and computational needs. Collectively, these findings underscore the importance of strategic investments in larger, more efficient transportation systems to mitigate CO_2 emissions and align with global sustainability targets. Transportation-related emissions were estimated using the Freightos Carbon Calculator [30]. The calculation is based on the assumption that the packages are manufactured in Taiwan and shipped directly to

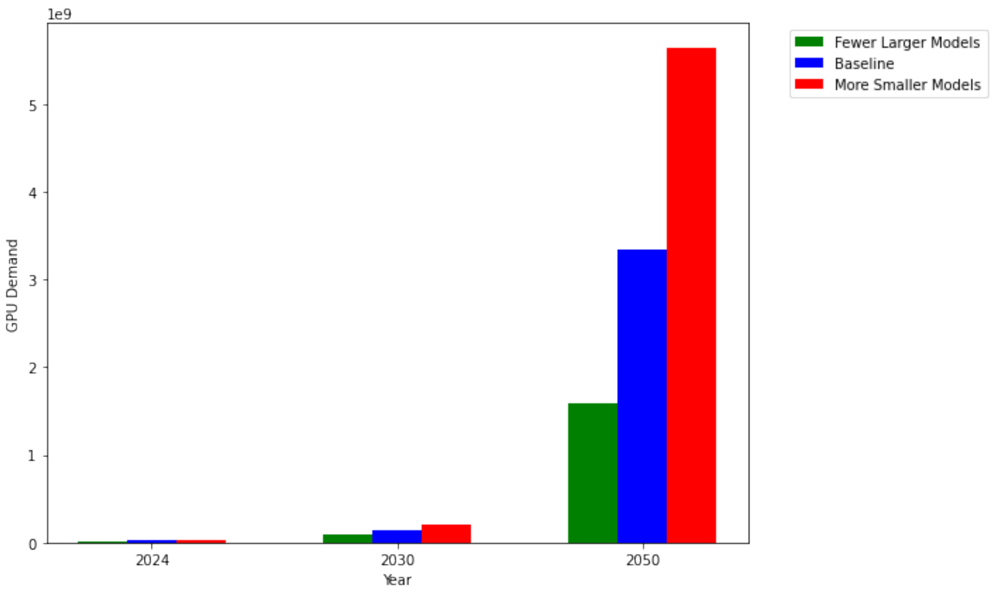

Figure 17: GPU Demand in 2024, 2030 and 2050

a U.S. port by air freight, with emissions determined according to the package size and distance traveled.

7. Electricity Carbon Footprint

In this study, we forecasted the energy demand for upcoming years based on prior calculations and estimations. The energy demand is divided into two main components: **usage** and **training**. The training energy demand represents the power consumption required for training artificial intelligence models, while the usage energy demand accounts for the energy consumption during model inference and operational phases.

The forecasting is based on three different scenarios:

- **Baseline**: A standard growth model for energy consumption.
- **Fewer, Larger Models**: Assumes a scenario where fewer but larger models are trained, affecting energy consumption patterns.
- **More, Smaller Models**: Represents a scenario with a higher number of smaller models being trained, leading to different energy distribution.

The breakdown of energy demand by year is presented in Tables 8 and 9.

Year	Training (Baseline)	Training (Fewer, Larger)	Training (More, Smaller)	Usage (Baseline)	Usage (Fewer, Larger)	Usage (More, Smaller)
2024	122.22	95.91	146.65	96.27	96.27	96.27
2025	185.19	136.32	231.09	94.43	94.43	94.43
2026	253.88	177.70	327.40	98.29	98.29	98.29
2027	336.98	226.41	446.43	108.25	108.25	108.25
2028	441.22	286.18	598.10	124.52	124.52	124.52
2029	572.27	359.82	791.40	147.71	147.71	147.71
2030	734.87	449.72	1034.13	178.86	178.86	178.86
2040	4996.69	2576.22	7872.94	1269.12	1269.12	1269.12
2050	11500.00	6000.00	17000.00	3100.00	3100.00	3100.00

Table 8: Forecasted Energy Demand in Absolute Terms (in TwH) by Year

Year	Usage % (Baseline)	Usage % (Fewer, Larger)	Usage % (More, Smaller)
2024	0.4406	0.5009	0.3963
2025	0.3377	0.4092	0.2901
2026	0.2791	0.3561	0.2309
2027	0.2431	0.3235	0.1952
2028	0.2201	0.3032	0.1723
2029	0.2052	0.2910	0.1573
2030	0.1958	0.2846	0.1475
2040	0.2025	0.3300	0.1388
2050	0.1800	0.3000	0.1200

Table 9: Forecasted Energy Demand in Percentage Terms by Year

The results indicate that energy consumption varies significantly based on different training scenarios. Further insights into CO_2 emissions resulting from these energy demands will be discussed in the next section.

7.1 Cost of Electricity Generation

7.1.1 Step 1: Energy Pricing

To determine the energy price per unit of electricity produced, we employed a systematic three-step methodology:

1. **Collection of Production Cost Data:** We first gathered the **Production Euros Output** (in euros) for each country and energy source. These values were derived using a **Multi-Regional Input-Output (MRIO)-based approach**, ensuring a comprehensive and standardized economic assessment of energy production costs.

7.1.2 Multi-Regional Input-Output Model

All the life cycle impacts related to electricity production and GPU manufacturing have been evaluated using a global MRIO model. In this model, the inputs and outputs of an economy are represented using Leontief's inverse formula, as shown in Equation (1):

$$x = (IA)^{-1}y \tag{25}$$

where x is the output vector, I is the identity matrix, A is an input-output coefficient matrix (expressed in M€/M€), and y represents the column vector of total usage (expressed in M€).

The term $(IA)^{-1}$ is commonly called the Leontief inverse, denoted as L, which expresses the total requirements matrix. The total inputs required to create one unit of sector output are shown for each element of A's direct requirement matrix. A global MRIO model uses this relationship, along with sector-specific environmental satellite accounts, to assess the effects associated with a unit of output from a particular sector as well as any indirect effects resulting from the industry's global supply chains [28]. The matrix of intensities, denoted as B, is presented in Equation (2). It represents the direct and indirect requirements of each industry for the outputs of other industries:

$$B = E \times (\text{diag}(x))^{-1} \tag{26}$$

where B is the matrix of intensities (expressed in per M€), E is the satellite account matrix which represents the total direct carbon footprints per unit of output of an industry, and x is the output vector.

Finally, we use Equation (2) to calculate the total environmental impacts of each industry. These impacts are encompassed in vector r, allowing us to track GHG emissions across regional and global supply networks:

$$r = BLy \tag{27}$$

where r represents the impact matrix, B is the matrix of intensities, L is the total requirements matrix, and y is the column vector of total usage.

In this paper, we utilize the EXIOBASE 3.8 database as our global MRIO dataset. Electricity production-related emissions are estimated for each country's electricity consumption in data centers located within that country. GPU manufacturing emissions are specifically attributed to GPUs produced in Taiwan.

To calculate the total impacts of these processes, we express the economic output in M€and denote it as EO. We then multiply it by r to determine the total direct and indirect carbon footprint impacts of each process, as shown in Equation (4):

$$TI = EO \times r \tag{28}$$

where TI represents the total impact, EO is the economic output (M€), and r is the impact sectors matrix (Impact/M€). Table 10 in the Supplementary Information (SI) details the sectoral classifications used for each process in the life cycle carbon footprints of electricity production and GPU manufacturing.

2. **Electricity Generation Data Acquisition:** Next, we compiled data on **Electricity Output** (in terawatt-hours, TWh) for the same countries and energy sources. This dataset provides insights into the actual energy generation capacity associated with different production expenditures.

3. **Calculation of Energy Price per Unit:** Finally, we computed the **Price Table** (in euros per TWh) by dividing the total **Production Euros Output** by the corresponding **Electricity Output**. This step enables an estimation of the cost per unit of electricity generated across various energy sources and countries.

This initial calculation serves as the foundation for subsequent assessments of **carbon emissions and environmental impact** by establishing the economic parameters of electricity production.

Table 10: Production Euros Output (in euros)by country and energy type

Entity	Other ren	Bioenergy	Solar	Wind	Hydro	Nuclear	Oil	Gas	Coal
Australia	11817539.69	194048880.8	42296317.54	304616074.6	1753304790	0	352136080.8	3762267057	21055015234
Brazil	8722609.151	328894547.9	0	25500562.45	6219311140	178488682.5	288152202	793614970.4	43492839.17
Canada	911374.788	172633219.5	575110.1079	128328437.7	8266895951	2016567560	254182108.6	1373632111	2449745316
China	607659227.8	710678310.1	28744171.77	1573800032	64903584787	8348738471	2096916648	6774709436	322019642104
France	125234470.5	1188528426	2782000.286	388430327.1	4134541253	36248924473	1222220175	6638759574	2640008554
Germany	242324743.5	2803285741	385166007.1	3534777148	1512971406	11429298153	597669125.5	9183316016	26563417845
India	17572847.04	383079515.4	1943854.701	1086144619	8818373666	1522618575	1523861062	7068386676	46562285615
Indonesia	53537736.51	3416890.801	0	0	488388956.3	0	2124917270	909546447.1	3121977438
Japan	305197909.4	3784069576	55542575.29	168661746.2	2088122732	39832912104	22758051366	37658675133	39497006989
Netherlands	48230478.98	100017344.1	597684.5321	166674479.8	6071123.793	211939342	112934457.9	1379862085	1263410721
Spain	56571459.72	575746347	172405106.1	4324310335	4016232762	8383264347	2973781275	15238809681	9522994804
United Kingdom	36695005.01	751199210.7	21402981.53	486898467.4	346943224.6	4710629793	628171776.1	25750131568	8865383239
United States	270541148.8	3471134109	92139678.26	2627366455	13333508287	41485226920	2698639863	52822335828	102841483951

Table 11: Electricity Output (TwH) by country and energy type in 2021. Data Source [25]

Country	Other ren	Bioenergy	Solar	Wind	Hydro	Nuclear	Oil	Gas	Coal
Australia	0.0	3.34	31.19	26.8	15.94	0.0	5.15	47.63	137.4
Brazil	0.0	55.72	16.75	72.29	362.82	14.7	22.63	86.96	24.24
Canada	0.0	9.86	5.17	34.76	382.79	91.98	3.01	83.37	35.83
China	0.0	165.93	327.0	655.8	1300.0	407.5	62.1	287.1	5328.83
France	0.58	9.57	15.37	36.83	59.62	379.36	10.06	33.29	5.44
Germany	0.24	49.1	49.34	114.65	19.66	69.13	22.24	90.3	164.65
India	0.0	36.76	68.31	68.09	160.33	43.92	3.38	59.84	1274.13
Indonesia	15.91	14.96	0.19	0.44	24.7	0.0	6.67	56.53	189.96
Japan	0.0	35.76	92.4	8.25	79.58	61.22	38.14	365.76	338.6
Netherlands	0.0	10.92	11.3	18.0	0.09	3.83	5.33	56.69	14.6
Spain	0.02	6.94	27.1	62.06	29.63	56.56	12.08	71.5	4.86
United Kingdom	0.0	39.88	12.14	64.66	5.5	45.9	10.89	123.17	6.51
United States	18.24	54.25	164.42	378.2	246.47	779.65	35.2	1579.19	898.0

Table 12: Price Table(in euro/TWh) by country and energy type

Entity	Other ren	Bioenergy	Solar	Wind	Hydro	Nuclear	Oil	Gas	Coal
Australia	-	58098467.31	1356085.85	11366271.44	109994026.98	-	68375938.02	78989440.63	153238829.94
Brazil	-	5902630.08	0	352753.67	17141588.50	12142087.24	12733194.96	9126207.11	1794259.04
Canada	-	17508440.11	111239.87	3691842.28	21596426.11	21923978.69	84445883.26	16476335.74	68371345.69
China	-	4283000.72	87902.67	2399817.07	49925834.45	20487701.77	33766773.72	23597037.39	60429708.23
France	215921500.86	124193147.96	181001.97	10546574.18	69348226.32	95552837.60	121493059.14	199422035.87	485295690.07
Germany	1009686431.25	57093395.95	7806364.15	30831026.15	76956836.52	165330509.95	26873611.76	101697851.78	161332631.92
India	-	10421096.72	28456.37	15951602.57	55001395.04	34668000.34	450846468.05	118121435.09	36544375.86
Indonesia	3365036.86	228401.79	0	0	19772832.24	-	318578301.35	16089624.04	16434920.18
Japan	-	105818500.45	601110.12	20443848.02	26239290.42	650651945.51	596697728.53	102960069.81	116647982.84
Netherlands	-	9159097.45	52892.44	9259693.32	67456931.03	55336642.82	21188453.64	24340484.83	86534980.89
Spain	2828572986	82960568.73	6361812.03	69679509.10	135546161.39	148218959.46	246173946.61	213130205.33	1959463951.44
United Kingdom	-	18836489.74	1763013.31	7530134.05	63080586.29	102628100.07	57683358.69	209061716.07	1361810021.35
United States	14832299.82	63984038.88	560392.16	6947029.23	54097895.43	53210064.67	76665905.20	33449006.03	114522810.64

7.1.3 Step 2: Emission Factor

The emission factor calculation follows a fundamental methodology based on economic and environmental impact analysis. First, the Global Warming Potential (GWP) values, which represent the kilograms of CO_2 emitted per million euros of energy production, are sourced for each energy type and country. These values provide a measure of the carbon intensity associated with monetary expenditures on electricity generation. Next, the electricity price per unit output, obtained from the first step of the study (measured in euro/TWh), is utilized as a scaling factor to translate monetary-based emissions into an energy-based emission metric. By multiplying the GWP values by the corresponding electricity price factors, we derive the Emission Factor Table **kgCO_2/TWh**. This final dataset offers a standardized representation of CO_2 emissions per terawatt-hour of electricity production, allowing for direct comparisons across different energy sources and geographic regions.

The resulting emission factor table, Table 13 provides a fundamental basis for further carbon footprint assessments and policy implications.

Table 13: GWP: $kgCO_2$/million euro by country and energy type

Energy Source	Australia	Brazil	Canada	China	France	Germany	India	Japan	Netherlands	United Kingdom	United States	Spain	Indonesia
Coal	10418093.33	226562892.5	22445454.23	12321465.12	4313297.34	6884719.24	24805307.16	6869427.28	11372860.68	7442315.58	13408867.75	6163107.24	6163107.24
Gas	10510284.59	67961742.89	14206084.06	13628122.56	1693638.94	763460.15	4185417.03	6111591.46	1803539.44	1445666.13	13262039.13	1431722.77	1431722.77
Nuclear	-	218781.39	50272.14	265775.14	196630.55	103967.85	161499.37	430690.8	270158.03	32294.6	23868.63	213430.13	213430.13
Hydro	164089.09	48699.74	80813.74	309421.43	166663.93	102433.69	169206.02	73822.38	157217.89	179405.42	85652.68	232166.1	232166.1
Wind	259163.87	817513.1	4405842.52	315724.53	573640.03	99890.23	596840.24	99563.43	1498485.51	3326124.6	45414.66	491708.11	491708.11
Oil	5447571.78	74796493.14	22316955.89	1694318.78	1312469.9	2129095.76	3386486.47	2389980.78	521142.59	1293179.4	5228591.05	3332140.11	3332140.11
Bioenergy	6637447.77	144367.19	832275.82	1547418.19	421634.31	2040459.5	421139.71	3497479.03	229830.05	1686212.3	4336423.45	706799.99	706799.99
Solar	70245.89	-	1286828225	4567513	1460374.21	4644501.83	119096718	118589.98	7918986.31	15905491.27	4537594.44	86040.1	86040.1
Other ren	263232.62		2593159913	208367680.6	176995.62		34191945.85	194230.65	8.37524E+13		45431.705	30120977.88	30120977.88
Other ren	263232.62	-	-	32.12	-	-	-	-	4.56215E+13	-	56888.62	30120977.88	30120977.88
Other ren	-	-	2593159913	3159932.61	176995.62	-	-	7467.41	2.05626E+14	-	-	-	-
Other ren	-	-	-	621943077.2	-	-	34191945.85	380993.89	9710228519	-	33974.79	-	-

Table 14: Emission Factor GWP*Price: kgCO_2/TwH by country and energy type

Country	Other ren	Bioenergy	Solar	Wind	Hydro	Nuclear	Oil	Gas	Coal
Australia	0	385625542	95259.4572	2945726.89	18048819.8	0	372482830	830201501	1596456432
Brazil	0	852146.118	0	288380.742	834790.903	2656462.72	952398330	620232941	406512518
Canada	0	14571851.4	143146600	16265675.7	1745287.96	1102165.33	1884575052	234064211	1534625910
China	0	6627593.23	401496.57	757681.115	15448123.1	5445121.81	57211678.9	321583318	744582542
France	38217159.9	52364092.2	264330.61	6049937.13	11557847.9	18788607	159455983	337748925	2093224609
Germany	0	116496762	36256672.6	3079718.29	7882972.74	17189057.7	57216492.9	77642257.2	1110729875
India	0	4388737.65	3389060.39	9520558.31	9306567.15	5598860.21	1526785464	494387466	906494468
Indonesia	101358201	161434.384	0	0	4590581.35	0	1061547536	23035881.1	101290176
Japan	0	370097986	71285.6374	2035459.63	1937046.87	280229807	1426096103	629249883	801304835
Netherlands	0	2105035.82	418854.48	13875516.3	10605436.4	14949638.4	11042205.6	43899024.4	984150282
Spain	85199000000	58636529.2	547370.944	34261979.7	31469223.7	31634391.8	820286082	305143368	12076000000
United Kingdom	0	31762320.7	28041592.7	25046164.1	11316999.1	3314333.44	74594931.2	302233442	10135000000
United States	673856.67	277461887	2542832.33	315496.971	4633629.73	1270051.35	400854666	443602027	1535621222

As a result of these calculations, the emission factor was determined in units of **$kgCO_2$/TWh**.

Emission Factors for Hong Kong and Singapore

In this section, we provide specific emission factors ($kgCO_2$/kWh) for Hong Kong and Singapore, which were previously excluded from the main GWP table.

Table 15: Emission Factors for Hong Kong and Singapore ($kgCO_2$/kWh)

Entity	Code	Year	Coal	Gas	Oil	Nuclear	Hydro	Wind	Solar	Bioenergy	Other Renewables
Hong Kong	HKG	2021	0.9158[22]	0.4201[22]	0.7556[22]	0.0051[24]	0.0169	0.0173	0.05[23]	0.24[21]	0.0
Singapore	SGP	2021	0.9158[22]	0.4201[22]	0.7556[22]	0.0051[24]	0.0169	0.0173	0.05[23]	0.24[21]	0.0

7.2 Energy Demand

7.2.1 Step 3: Energy Demand Allocation

To estimate the energy demand associated with data centers, we assumed that the global distribution of data centers remains constant over time. Based on this assumption, we selected the top 15 countries that collectively host 82.67% of the world's data centers. These countries serve as a representative subset for global energy demand projections.

The energy demand model incorporates total energy consumption derived from both training and operational phases of data center activities. We distributed this total energy demand among the selected 15 countries based on their respective shares of global data centers. This distribution was performed for both 2030 and 2050, allowing for further analysis in subsequent steps based on each country's renewable energy targets.

The total energy demand (TWh) is represented in three different scenarios: **Baseline**, **Fewer Larger** (assuming fewer but larger data centers), and **More Smaller** (assuming a greater number of smaller data centers).

Table 16: Total Energy Demand Projections (TWh).

Year	Baseline	Fewer Larger	More Smaller
2030	914	629	1213
2050	20914	12236	32049

The allocation of energy demand by country for 2030 and 2050 is detailed in Tables 17 and 18, respectively.

This structured allocation ensures that energy demand projections align with current global data center distributions while enabling forward-looking analyses for 2030 and 2050. Future calculations will incorporate renewable energy targets for these countries to assess potential sustainability scenarios.

Table 17: Energy demand allocation by country based on global data center distribution for 2030 (TWh).

Country Name	Data Centers	% Share	Baseline	Fewer Larger	More Smaller
United States	1958	49.0%	448	308	594
China	375	9.4%	86	59	114
United Kingdom	243	6.1%	56	38	74
Germany	198	5.0%	45	31	60
Canada	168	4.2%	38	26	51
France	143	3.6%	33	22	43
India	141	3.5%	32	22	43
Netherlands	138	3.5%	32	22	42
Australia	132	3.3%	30	21	40
Japan	121	3.0%	28	19	37
Brazil	118	3.0%	27	19	36
Singapore	96	2.4%	22	15	29
Hong Kong	59	1.5%	13	9	18
Spain	57	1.4%	13	9	17
Indonesia	49	1.2%	11	8	15

Table 18: Energy demand allocation by country based on global data center distribution for 2050 (TWh).

Country Name	Data Centers	% Share	Baseline	Fewer Larger	More Smaller
United States	1958	49.0%	10,248	5,996	15,704
China	375	9.4%	1,963	1,148	3,008
United Kingdom	243	6.1%	1,272	744	1,949
Germany	198	5.0%	1,036	606	1,588
Canada	168	4.2%	879	514	1,347
France	143	3.6%	748	438	1,147
India	141	3.5%	738	432	1,131
Netherlands	138	3.5%	722	423	1,107
Australia	132	3.3%	691	404	1,059
Japan	121	3.0%	633	371	970
Brazil	118	3.0%	618	361	946
Singapore	96	2.4%	502	294	770
Hong Kong	59	1.5%	309	181	473
Spain	57	1.4%	298	175	457
Indonesia	49	1.2%	256	150	393

7.2.2 Step 4: Renewable and Non-Renewable Energy Distribution

To evaluate how the allocated total energy demand will be met by renewable and non-renewable sources, we examined the energy production targets of the selected countries for 2030 and 2050. These targets define the percentage of electricity expected to be generated from renewable and non-renewable sources in each year. The data sources for these targets are detailed in Tables 19 and 20.

Table 19: Energy Production Targets by Country for 2030.

Country	Renewable %	Non-Renewable %	Source
Australia	82%	18%	Ember Energy
Brazil	84%	16%	New Climate
Canada	72%	28%	Ember Energy
China	60%	40%	Energy Transitions
France	41%	59%	Enerdata
Germany	80%	20%	IEA
Hong Kong	27%	73%	Civic Exchange
India	50%	50%	Power Ministry India
Indonesia	44%	56%	Ember Energy
Japan	38%	62%	Reuters
Singapore	30%	70%	Green Plan Singapore
Netherlands	32%	68%	EU Energy Report
Spain	78%	22%	Ember Energy
United Kingdom	70%	30%	RUSI
United States	33%	67%	ASES

These structured tables ensure that the energy production targets for all selected countries are accurately represented.

Table 20: Energy Production Targets by Country for 2050.

Country	Renewable %	Non-Renewable %	Source
Australia	100%	0%	Industry Australia
Brazil	100%	0%	EKOS Brazil
Canada	100%	0%	CER Canada
China	100%	0%	Energy Transitions
France	100%	0%	RTE France
Germany	100%	0%	SDG Partnership
Hong Kong	36%	64%	EMSD Hong Kong
India	100%	0%	WWF
Indonesia	66%	34%	IRENA
Japan	55%	45%	European Parliament
Singapore	100%	0%	EMA Singapore
Netherlands	100%	0%	Government Netherlands
Spain	100%	0%	The Guardian
United Kingdom	100%	0%	National Grid
United States	44%	56%	EIA

7.2.3 Step 4.1A: Energy Demand Distribution by Scenario for 2030

To further analyze the energy demand distribution in 2030, we calculated the share of total energy demand met by renewable and non-renewable sources under different scenarios: **Baseline**, **Fewer Larger**, and **More Smaller**. These values were determined based on each country's energy mix targets and weighted global warming potential (GWP). The results are summarized in Tables 21, 22, and 23.

Table 21: Allocation of Renewable and Non-Renewable Energy in 2030 - Baseline Scenario (TWh)

Country	Code	Renewable Energy (TWh)	Non-Renewable Energy (TWh)
Australia	AU	25	5
Brazil	BR	23	4
Canada	CA	28	11
China	CN	51	34
France	FR	13	19
Germany	DE	36	9
Hong Kong	HK	4	10
Singapore	SG	7	15
India	IN	16	16
Indonesia	ID	5	6
Japan	JP	11	17
Netherlands	NL	10	21
Spain	ES	10	3
United Kingdom	GB	39	17
United States	US	148	300

Table 22: Allocation of Renewable and Non-Renewable Energy in 2030 - Fewer Larger Scenario (TWh)

Country	Code	Renewable Energy (TWh)	Non-Renewable Energy (TWh)
Australia	AU	17	4
Brazil	BR	16	3
Canada	CA	19	7
China	CN	35	24
France	FR	9	13
Germany	DE	25	6
Hong Kong	HK	3	7
Singapore	SG	5	11
India	IN	11	11
Indonesia	ID	3	4
Japan	JP	7	12
Netherlands	NL	7	15
Spain	ES	7	2
United Kingdom	GB	27	11
United States	US	102	206

Table 23: Allocation of Renewable and Non-Renewable Energy in 2030 - More Smaller Scenario (TWh)

Country	Code	Renewable Energy (TWh)	Non-Renewable Energy (TWh)
Australia	AU	33	7
Brazil	BR	30	6
Canada	CA	37	14
China	CN	68	46
France	FR	18	26
Germany	DE	48	12
Hong Kong	HK	5	13
Singapore	SG	9	20
India	IN	21	21
Indonesia	ID	7	8
Japan	JP	14	23
Netherlands	NL	13	28
Spain	ES	13	4
United Kingdom	GB	52	22
United States	US	196	398

7.2.4 Step 4.1B: Energy Demand Distribution by Scenario for 2050

To further analyze the energy demand distribution in 2050, we calculated the share of total energy demand met by renewable and non-renewable sources under different scenarios: **Baseline**, **Fewer Larger**, and **More Smaller**. These values were determined based on each country's energy mix targets and weighted global warming potential (GWP). The energy mix targets used for these calculations were sourced from Table 20. The results are summarized in Tables 24, 25, and 26.

Table 24: Energy Demand Distribution by Fuel Type in 2050 - Baseline Scenario (TWh)

**Country**	**Code**	**Renewable Energy (TWh)**	**Non-Renewable Energy (TWh)**
Australia	AU	691	0
Brazil	BR	618	0
Canada	CA	879	0
China	CN	1963	0
France	FR	748	0
Germany	DE	1036	0
Hong Kong	HK	112	197
Singapore	SG	502	0
India	IN	487	251
Indonesia	ID	141	115
Japan	JP	633	0
Netherlands	NL	722	0
Spain	ES	298	0
United Kingdom	GB	1272	0
United States	US	4509	5739

These tables summarize the energy distribution by fuel type for 2050 across all scenarios, ensuring a detailed comparison of renewable and non-renewable energy contributions.

Table 25: Energy Demand Distribution by Fuel Type in 2050 - Fewer Larger Scenario (TWh)

Country	Code	Renewable Energy (TWh)	Non-Renewable Energy (TWh)
Australia	AU	404	0
Brazil	BR	361	0
Canada	CA	514	0
China	CN	1148	0
France	FR	438	0
Germany	DE	606	0
Hong Kong	HK	65	115
Singapore	SG	294	0
India	IN	285	147
Indonesia	ID	83	68
Japan	JP	371	0
Netherlands	NL	423	0
Spain	ES	175	0
United Kingdom	GB	744	0
United States	US	2638	3358

Table 26: Energy Demand Distribution by Fuel Type in 2050 - More Smaller Scenario (TWh)

Country	Code	Renewable Energy (TWh)	Non-Renewable Energy (TWh)
Australia	AU	1059	0
Brazil	BR	946	0
Canada	CA	1347	0
China	CN	3008	0
France	FR	1147	0
Germany	DE	1588	0
Hong Kong	HK	172	302
Singapore	SG	770	0
India	IN	746	384
Indonesia	ID	216	177
Japan	JP	970	0
Netherlands	NL	1107	0
Spain	ES	457	0
United Kingdom	GB	1949	0
United States	US	6910	8794

7.2.5 Step 4.2: Allocation of Renewable and Non-Renewable Energy by Fuel Type

To ensure consistency in our emissions calculations, we further decomposed the allocated renewable and non-renewable energy into specific fuel types. This step was necessary to facilitate a structured matrix multiplication using the emission factor table computed earlier.

We examined the current electricity production mix of each country and assumed that, while the total amounts of non-renewable energy (coal, gas, and oil) and renewable energy (bioenergy, solar, wind, hydro, and nuclear) may change over time, their respective shares within their category would remain constant. This means that if, for a given country, coal currently accounts for $x\%$ of its total non-renewable energy production, we assume that in 2030, coal will still contribute $x\%$ of the country's total non-renewable generation. Similarly, for renewables, the relative share of bioenergy, solar, wind, hydro, and nuclear will remain unchanged over time, even as their total contributions evolve.

This approach ensures that even though the absolute quantities of renewable and non-renewable energy shift due to changing energy demands and targets, the internal distribution within each category remains stable. By maintaining these proportional relationships, our analysis remains consistent and allows for accurate emissions projections.

The current electricity production mix by fuel type for each country in 2021 is summarized in Table 27. This mix is used to distribute the projected renewable and non-renewable energy in 2030 across fuel types for each scenario. The data is sourced from [25].

Table 27: Electricity Production Mix by Fuel Type for 2021 (% of Total Electricity Production). Data Source: [25].

Entity	Code	Other Ren.	Bioenergy	Solar	Wind	Hydro	Nuclear	Oil	Gas	Coal
Australia	AUS	0.0%	4.3%	40.4%	34.7%	20.6%	0.0%	2.7%	25.0%	72.2%
Brazil	BRA	0.0%	10.7%	3.2%	13.8%	69.5%	2.8%	16.9%	65.0%	18.1%
Canada	CAN	0.0%	1.9%	1.0%	6.6%	73.0%	17.5%	2.5%	68.2%	29.3%
China	CHN	0.0%	5.8%	11.4%	23.0%	45.5%	14.3%	1.1%	5.1%	93.8%
France	FRA	0.1%	1.9%	3.1%	7.3%	11.9%	75.7%	20.6%	68.2%	11.1%
Germany	DEU	0.1%	16.3%	16.3%	37.9%	6.5%	22.9%	8.0%	32.6%	59.4%
Hong Kong	HKG	0.0%	5.0%	3.2%	0.0%	0.0%	91.7%	0.5%	60.2%	39.3%
Singapore	SNG	0.0%	75.3%	24.7%	0.0%	0.0%	0.0%	1.0%	97.7%	1.2%
India	IND	0.0%	9.7%	18.1%	18.0%	42.5%	11.6%	0.3%	4.5%	95.3%
Indonesia	IDN	28.3%	26.6%	0.3%	0.8%	44.0%	0.0%	2.6%	22.3%	75.0%
Japan	JPN	0.0%	12.9%	33.3%	3.0%	28.7%	22.1%	5.1%	49.3%	45.6%
Netherlands	NLD	0.0%	24.7%	25.6%	40.8%	0.2%	8.7%	7.0%	74.0%	19.1%
Spain	ESP	0.0%	3.8%	14.9%	34.0%	16.3%	31.0%	13.7%	80.8%	5.5%
United Kingdom	GBR	0.0%	23.7%	7.2%	38.5%	3.3%	27.3%	7.7%	87.6%	4.6%
United States	USA	1.1%	3.3%	10.0%	23.0%	15.0%	47.5%	1.4%	62.9%	35.7%

The energy production mix from this table is used to allocate the 2030 projected energy across different fuel types for each scenario.

7.3 Total Carbon Footprint

7.3.1 Step 4.3A: Energy Distribution by Fuel Type for 2030 Projections

Following the allocation of renewable and non-renewable energy for each country, we further distributed these energy sources based on their fuel type proportions. This step ensures that the emission factor matrix multiplication remains consistent with each country's current energy mix structure while adapting to the projected total energy values in 2030.

We assumed that while the absolute amount of non-renewable energy (coal, gas, and oil) and renewable energy (bioenergy, solar, wind, hydro, and nuclear) may change over time, the internal distribution of these sources remains proportionally consistent with their 2021 levels. This approach allows for a more accurate and structured analysis of emissions trends.

The following tables present the fuel-type distribution of energy demand in 2030 across three different scenarios: **Baseline**, **Fewer Larger**, and **More Smaller**. All countries are included in each scenario to ensure a comprehensive analysis.

Table 28: Energy Distribution by Fuel Type in 2030 - Baseline Scenario (TWh).

Entity	Other Ren.	Bioenergy	Solar	Wind	Hydro	Nuclear	Oil	Gas	Coal
Australia	0.0	1.1	10.0	8.6	5.1	0.0	0.1	1.4	3.9
Brazil	0.0	2.4	0.7	3.1	15.7	0.6	0.7	2.8	0.8
Canada	0.0	0.5	0.3	1.8	20.2	4.8	0.3	7.3	3.2
China	0.0	3.0	5.9	11.8	23.4	7.3	0.4	1.7	32.2
France	0.0	0.3	0.4	1.0	1.6	10.1	4.0	13.2	2.2
Germany	0.0	5.9	5.9	13.7	2.4	8.3	0.7	2.9	5.4
Hong Kong	0.0	0.2	0.1	0.0	0.0	3.4	0.1	5.9	3.9
Singapore	0.0	5.0	1.6	0.0	0.0	0.0	0.2	15.0	0.2
India	0.0	1.6	2.9	2.9	6.8	1.9	0.0	0.7	15.4
Indonesia	1.4	1.3	0.0	0.0	2.2	0.0	0.2	1.4	4.7
Japan	0.0	1.4	3.5	0.3	3.0	2.3	0.9	8.5	7.8
Netherlands	0.0	2.5	2.6	4.1	0.0	0.9	1.5	15.9	4.1
Spain	0.0	0.4	1.5	3.5	1.7	3.2	0.4	2.3	0.2
United Kingdom	0.0	9.2	2.8	15.0	1.3	10.6	1.3	14.6	0.8
United States	1.6	4.9	14.8	34.0	22.2	70.2	4.2	188.6	107.2

Table 29: Energy Distribution by Fuel Type in 2030 - Fewer Larger Scenario (TWh).

Entity	Other Ren.	Bioenergy	Solar	Wind	Hydro	Nuclear	Oil	Gas	Coal
Australia	0.0	0.7	6.9	5.9	3.5	0.0	0.1	0.9	2.7
Brazil	0.0	1.7	0.5	2.2	10.8	0.4	0.5	1.9	0.5
Canada	0.0	0.4	0.2	1.3	13.9	3.3	0.2	5.0	2.2
China	0.0	2.1	4.1	8.1	16.1	5.0	0.3	1.2	22.1
France	0.0	0.2	0.3	0.7	1.1	7.0	2.7	9.1	1.5
Germany	0.0	4.0	4.1	9.5	1.6	5.7	0.5	2.0	3.7
Hong Kong	0.0	0.1	0.1	0.0	0.0	2.3	0.0	4.1	2.7
Singapore	0.0	3.4	1.1	0.0	0.0	0.0	0.1	10.3	0.1
India	0.0	1.1	2.0	2.0	4.7	1.3	0.0	0.5	10.6
Indonesia	1.0	0.9	0.0	0.0	1.5	0.0	0.1	1.0	3.2
Japan	0.0	0.9	2.4	0.2	2.1	1.6	0.6	5.8	5.4
Netherlands	0.0	1.7	1.8	2.8	0.0	0.6	1.0	10.9	2.8
Spain	0.0	0.3	1.0	2.4	1.1	2.2	0.3	1.6	0.1
United Kingdom	0.0	6.3	1.9	10.3	0.9	7.3	0.9	10.0	0.5
United States	1.1	3.4	10.2	23.4	15.3	48.3	2.9	129.7	73.8

Table 30: Energy Distribution by Fuel Type in 2030 - More Smaller Scenario (TWh).

Entity	Other Ren.	Bioenergy	Solar	Wind	Hydro	Nuclear	Oil	Gas	Coal
Australia	0.0	1.4	13.3	11.4	6.8	0.0	0.2	1.8	5.2
Brazil	0.0	3.2	1.0	4.2	20.9	0.8	1.0	3.7	1.0
Canada	0.0	0.7	0.4	2.4	26.8	6.4	0.4	9.7	4.2
China	0.0	4.0	7.8	15.7	31.1	9.7	0.5	2.3	42.7
France	0.0	0.3	0.5	1.3	2.1	13.5	5.3	17.5	2.9
Germany	0.0	7.8	7.9	18.2	3.1	11.0	1.0	3.9	7.1
Hong Kong	0.0	0.2	0.2	0.0	0.0	4.5	0.1	7.8	5.1
Singapore	0.0	6.6	2.2	0.0	0.0	0.0	0.2	19.9	0.3
India	0.0	2.1	3.9	3.9	9.1	2.5	0.1	1.0	20.4
Indonesia	1.9	1.7	0.0	0.1	2.9	0.0	0.2	1.9	6.3
Japan	0.0	1.8	4.7	0.4	4.0	3.1	1.2	11.2	10.4
Netherlands	0.0	3.3	3.4	5.5	0.0	1.2	2.0	21.1	5.4
Spain	0.0	0.5	2.0	4.6	2.2	4.2	0.5	3.1	0.2
United Kingdom	0.0	12.3	3.7	19.9	1.7	14.1	1.7	19.4	1.0
United States	2.2	6.5	19.6	45.2	29.5	93.2	5.6	250.3	142.3

7.3.2 Step 4.3b: Allocation of Renewable and Non-Renewable Energy and Energy Distribution by Fuel Type for 2050

To ensure consistency in our emissions calculations, we further decomposed the allocated renewable and non-renewable energy into specific fuel types. This step was necessary to facilitate a structured matrix multiplication using the emission factor table computed earlier.

We examined the current electricity production mix of each country and assumed that, while the total amounts of non-renewable energy (coal, gas, and oil) and renewable energy (bioenergy, solar, wind, hydro, and nuclear) may change over time, their respective shares within their category would remain constant. This means that if, for a given country, coal currently accounts for $x\%$ of its total non-renewable energy production, we assume that in 2050, coal will still contribute $x\%$ of the country's total non-renewable generation. Similarly, for renewables, the relative share of bioenergy, solar, wind, hydro, and nuclear will remain unchanged over time, even as their total contributions evolve.

This approach ensures that even though the absolute quantities of renewable and non-renewable energy shift due to changing energy demands and targets, the internal distribution within each category remains stable. By maintaining these proportional relationships, our analysis remains consistent and allows for accurate emissions projections.

The current electricity production mix by fuel type for each country in 2021 is summarized in Table 27. This mix is used to distribute the projected renewable and non-renewable energy in 2050 across fuel types for each scenario. The data is sourced from **Our World in Data**.

The energy distributions for different fuel types across the **Baseline**, **Fewer Larger**, and **More Smaller** scenarios are presented in Tables 31, 32, and 33, respectively. These tables provide a comprehensive breakdown of how energy demand is allocated across different sources in 2050.

Table 31: Energy Distribution by Fuel Type in 2050 - Baseline Scenario (TWh)

**Country**	**Other Ren.**	**Bioenergy**	**Solar**	**Wind**	**Hydro**	**Nuclear**	**Oil**	**Gas**	**Coal**
Australia	0.0	29.9	278.9	239.6	142.5	0.0	0.0	0.0	0.0
Brazil	0.0	65.9	19.8	85.5	429.0	17.4	0.0	0.0	0.0
Canada	0.0	16.5	8.7	58.3	641.6	154.2	0.0	0.0	0.0
China	0.0	114.0	224.7	450.6	893.3	280.0	0.0	0.0	0.0
France	0.9	14.3	22.9	55.0	89.0	566.3	0.0	0.0	0.0
Germany	0.8	168.4	169.2	393.3	67.4	237.1	0.0	0.0	0.0
Hong Kong	0.0	5.6	3.6	0.0	0.0	102.7	1.1	118.5	77.3
Singapore	0.0	378.4	124.1	0.0	0.0	0.0	0.0	0.0	0.0
India	0.0	47.4	88.2	87.9	206.9	56.7	0.6	11.2	239.0
Indonesia	39.9	37.5	0.5	1.1	62.0	0.0	3.0	25.8	86.6
Japan	0.0	81.7	211.1	18.8	181.8	139.9	0.0	0.0	0.0
Netherlands	0.0	178.7	184.9	294.5	1.5	62.7	0.0	0.0	0.0
Spain	0.0	11.4	44.3	101.6	48.5	92.6	0.0	0.0	0.0
United Kingdom	0.0	301.8	91.9	489.3	41.6	347.3	0.0	0.0	0.0
United States	50.1	149.0	451.7	1039.0	677.1	2142.0	80.4	3607.1	2051.2

These tables summarize the energy distribution by fuel type for 2050 across the Baseline,Fewer Larger and More Smaller scenarios.

Table 32: Energy Distribution by Fuel Type in 2050 - Fewer Larger Scenario (TWh)

Country	Other Ren.	Bioenergy	Solar	Wind	Hydro	Nuclear	Oil	Gas	Coal
Australia	0.0	17.5	163.2	140.2	83.4	0.0	0.0	0.0	0.0
Brazil	0.0	38.5	11.6	50.0	251.0	10.2	0.0	0.0	0.0
Canada	0.0	9.7	5.1	34.1	375.4	90.2	0.0	0.0	0.0
China	0.0	66.7	131.5	263.6	522.6	163.8	0.0	0.0	0.0
France	0.5	8.4	13.4	32.2	52.1	331.3	0.0	0.0	0.0
Germany	0.5	98.5	99.0	230.1	39.5	138.7	0.0	0.0	0.0
Hong Kong	0.0	3.3	2.1	0.0	0.0	60.1	0.6	69.3	45.2
Singapore	0.0	221.4	72.6	0.0	0.0	0.0	0.0	0.0	0.0
India	0.0	27.8	51.6	51.4	121.1	33.2	0.4	6.6	139.9
Indonesia	23.4	22.0	0.3	0.6	36.3	0.0	1.8	15.1	50.7
Japan	0.0	47.8	123.5	11.0	106.4	81.8	0.0	0.0	0.0
Netherlands	0.0	104.5	108.2	172.3	0.9	36.7	0.0	0.0	0.0
Spain	0.0	6.6	25.9	59.4	28.4	54.1	0.0	0.0	0.0
United Kingdom	0.0	176.5	53.7	286.2	24.3	203.2	0.0	0.0	0.0
United States	29.3	87.2	264.3	607.9	396.2	1253.2	47.0	2110.4	1200.1

Table 33: Energy Distribution by Fuel Type in 2050 - More Smaller Scenario (TWh)

Country	Other Ren.	Bioenergy	Solar	Wind	Hydro	Nuclear	Oil	Gas	Coal
Australia	0.0	45.8	427.3	367.2	218.4	0.0	0.0	0.0	0.0
Brazil	0.0	101.0	30.4	131.0	657.5	26.6	0.0	0.0	0.0
Canada	0.0	25.3	13.3	89.3	983.3	236.3	0.0	0.0	0.0
China	0.0	174.7	344.3	690.6	1368.9	429.1	0.0	0.0	0.0
France	1.3	21.9	35.2	84.3	136.4	867.9	0.0	0.0	0.0
Germany	1.3	258.1	259.3	602.6	103.3	363.4	0.0	0.0	0.0
Hong Kong	0.0	8.6	5.5	0.0	0.0	157.4	1.6	181.5	118.5
Singapore	0.0	579.8	190.1	0.0	0.0	0.0	0.0	0.0	0.0
India	0.0	72.7	135.1	134.7	317.1	86.9	1.0	17.2	366.3
Indonesia	61.2	57.5	0.7	1.7	95.0	0.0	4.7	39.5	132.7
Japan	0.0	125.2	323.5	28.9	278.6	214.3	0.0	0.0	0.0
Netherlands	0.0	273.8	283.3	451.3	2.3	96.0	0.0	0.0	0.0
Spain	0.1	17.4	68.0	155.6	74.3	141.8	0.0	0.0	0.0
United Kingdom	0.0	462.4	140.8	749.8	63.8	532.2	0.0	0.0	0.0
United States	76.8	228.4	692.2	1592.2	1037.7	3282.4	123.2	5527.7	3143.3

7.3.3 Step 4.4A: Calculation of Total Carbon Emissions for 2030

In this step, we calculate the total carbon emissions for each country in 2030 across all scenarios by leveraging the emission factors computed in **Step 2**. These emission factors, specific to each fuel type, are applied to the energy distribution obtained in **Step 4.3A** for each scenario.

**Overview of Methodology**
**Step 1: Emission Calculation by Fuel Type** The fuel-type-specific energy demands obtained in **Step 4.3A** are multiplied by the respective emission factors derived in **Step 2**, resulting in total CO_2 emissions per fuel type for each country.
**Step 2: Summation by Country** The emissions from all fuel types are aggregated for each country, providing total CO_2 emissions for 2030 under each scenario.
**Step 3: Segmentation into Training and Usage Emissions** The total CO_2 emissions are split into emissions from training and usage (operation) based on predefined scenario-specific percentages, as given in Table 9.
**Step 4: Final Calculation of Emissions by Usage and Training** Using the values in Table 8, we compute: $$E_{\text{usage}} = E_{\text{total}} \times P_{\text{usage}} \quad (29)$$ $$E_{\text{training}} = E_{\text{total}} \times (1 - P_{\text{usage}}) \quad (30)$$

Table 34: Structured overview of the methodology used for emission calculations across fuel types, countries, and scenarios. The methodology accounts for training and usage-based CO_2 emissions by integrating scenario-specific factors.

The total emission calculations are conducted through a structured approach, considering the energy demands and corresponding emission factors for different fuel types across multiple scenarios. The methodology consists of the following key steps.

First, the emissions for each fuel type in each country under different scenarios are determined by multiplying the fuel-type-specific energy demand with the respective emission factor:

$$E_{f,c,s} = D_{f,c,s} \times EF_f, \tag{31}$$

where $E_{f,c,s}$ represents the CO_2 emissions from fuel type f in country c under scenario s, $D_{f,c,s}$ is the energy demand for the given fuel type, and EF_f denotes the emission factor specific to the fuel type. The scenarios considered include Baseline, Fewer-Larger, and More-Smaller, which are

indexed by s.

Next, the total emissions for each country under each scenario are obtained by summing the contributions from all fuel types:

$$E_{c,s} = \sum_{f} E_{f,c,s} = \sum_{f} (D_{f,c,s} \times EF_f). \tag{32}$$

Following this, the total emissions are disaggregated into training emissions and usage (operation) emissions based on predefined scenario-specific percentages. The emissions from usage are computed as:

$$E_{c,s}^{\text{usage}} = E_{c,s} \times P_{\text{usage},s}, \tag{33}$$

where $P_{\text{usage},s}$ denotes the proportion of total emissions attributed to operational energy consumption under scenario s. Conversely, the emissions attributed to model training are determined as:

$$E_{c,s}^{\text{training}} = E_{c,s} \times (1 - P_{\text{usage},s}). \tag{34}$$

Finally, the total emissions from usage and training across all countries are obtained by summing over all individual country-level emissions:

$$E_{s}^{\text{usage}} = \sum_{c} E_{c,s}^{\text{usage}} = \sum_{c} (E_{c,s} \times P_{\text{usage},s}), \tag{35}$$

$$E_{s}^{\text{training}} = \sum_{c} E_{c,s}^{\text{training}} = \sum_{c} (E_{c,s} \times (1 - P_{\text{usage},s})). \tag{36}$$

Here, E_s^{usage} represents the total CO_2 emissions from operational energy consumption across all countries for scenario s, whereas E_s^{training} corresponds to the total emissions resulting from model training under the same scenario.

The total CO_2 emissions resulting from training and usage for each country in the Baseline, Fewer Larger, and More Smaller scenarios are presented in Tables 35, 36, and 37, respectively.

Table 35: Total CO_2 Emissions by Country for 2030 - Baseline Scenario ($kgCO_2$)

**Country**	**Usage Emissions**	**Training Emissions**
Australia	1.56E+09	6.42E+09
Brazil	5.42E+08	2.23E+09
Canada	1.40E+09	5.77E+09
China	4.89E+09	2.01E+10
France	1.92E+09	7.89E+09
Germany	1.44E+09	5.91E+09
Hong Kong	1.23E+09	5.06E+09
Singapore	1.54E+09	6.33E+09
India	2.83E+09	1.16E+10
Indonesia	1.64E+08	6.73E+08
Japan	2.74E+09	1.13E+10
Netherlands	9.42E+08	3.87E+09
Spain	6.50E+08	2.67E+09
United Kingdom	2.57E+09	1.06E+10
United States	4.92E+10	2.02E+11

Table 36: Total CO_2 Emissions by Country for 2030 - Fewer Larger Scenario ($kgCO_2$)

**Country**	**Usage Emissions**	**Training Emissions**
Australia	1.56E+09	3.93E+09
Brazil	5.42E+08	1.36E+09
Canada	1.40E+09	3.53E+09
China	4.89E+09	1.23E+10
France	1.92E+09	4.83E+09
Germany	1.44E+09	3.62E+09
Hong Kong	1.23E+09	3.09E+09
Singapore	1.54E+09	3.88E+09
India	2.83E+09	7.12E+09
Indonesia	1.64E+08	4.12E+08
Japan	2.74E+09	6.89E+09
Netherlands	9.42E+08	2.37E+09
Spain	6.50E+08	1.63E+09
United Kingdom	2.57E+09	6.46E+09
United States	4.92E+10	1.24E+11

Table 37: Total CO_2 Emissions by Country for 2030 - More Smaller Scenario ($kgCO_2$)

Country	Usage Emissions	Training Emissions
Australia	1.56E+09	9.03E+09
Brazil	5.42E+08	3.14E+09
Canada	1.40E+09	8.12E+09
China	4.89E+09	2.83E+10
France	1.92E+09	1.11E+10
Germany	1.44E+09	8.32E+09
Hong Kong	1.23E+09	7.11E+09
Singapore	1.54E+09	8.91E+09
India	2.83E+09	1.64E+10
Indonesia	1.64E+08	9.46E+08
Japan	2.74E+09	1.58E+10
Netherlands	9.42E+08	5.45E+09
Spain	6.50E+08	3.76E+09
United Kingdom	2.57E+09	1.49E+10
United States	4.92E+10	2.85E+11

Hong Kong-Specific Emission Calculation

Overview of Hong Kong Calculation Methodology

Hong Kong sources approximately **35%** of its electricity from imported nuclear energy from Mainland China. To accurately account for its emissions, we adopt the following approach.

1. Nuclear Import Adjustment: Since **25%** of Hong Kong's energy demand is met by imported nuclear power, this portion of the total energy demand is multiplied by China's nuclear emission factor to estimate the CO_2 emissions associated with this import.

2. Local Generation Calculation: The remaining **75%** of Hong Kong's energy demand follows the standard methodology outlined in **Step 4.4A**. Each fuel type is multiplied by its respective emission factor, ensuring an accurate emissions assessment.

3. Final Total Emissions: The emissions from China-imported nuclear energy and locally generated energy are summed to determine Hong Kong's total CO_2 emissions for each scenario.

Table 38: Structured overview of the methodology used for Hong Kong's emission calculations, incorporating both imported nuclear energy and local electricity generation.

Hong Kong sources approximately **35%** of its electricity from imported nuclear energy from Mainland China [26]. To accurately account for its emissions, the following methodology is applied.

First, an adjustment is made for **nuclear energy imports**. Since **25%** of Hong Kong's total energy demand is met by imported nuclear power [27], this portion of the demand is multiplied by China's nuclear emission factor to estimate the associated CO_2 emissions:

$$E_{\text{import,nuclear}} = D_{\text{import,nuclear}} \times EF_{\text{nuclear,China}} \tag{37}$$

where $E_{\text{import,nuclear}}$ represents the CO_2 emissions from imported nuclear energy, $D_{\text{import,nuclear}}$ is the imported nuclear energy demand, and $EF_{\text{nuclear,China}}$ is the nuclear emission factor for China.

The remaining **75%** of Hong Kong's energy demand is derived from local electricity generation. This follows the standard emissions calculation methodology outlined in **Step 4.4A**, where each fuel type's energy demand is multiplied by its respective emission factor:

$$E_{\text{local}} = \sum_{f} D_{\text{local},f} \times EF_f \tag{38}$$

where E_{local} represents the total CO_2 emissions from locally generated electricity, $D_{\text{local},f}$ is the local energy demand for fuel type f, and EF_f is the corresponding emission factor.

Finally, the **total CO_2 emissions for Hong Kong** are determined by summing emissions from imported nuclear energy and local generation:

$$E_{\text{Hong Kong}} = E_{\text{import,nuclear}} + E_{\text{local}} \tag{39}$$

This methodology ensures that the emissions contribution from Hong Kong's imported nuclear energy is appropriately accounted for while maintaining consistency with the calculations used for other regions.

These tables provides a breakdown of CO_2 emissions for each country in 2030 under the Baseline, Fewer Larger and More Smaller scenario, distinguishing between emissions from training and operation (usage).

7.3.4 Step 4.4B: Calculation of Total Carbon Emissions for 2050

In this step, we calculate the total carbon emissions for each country in 2050 across all scenarios by leveraging the emission factors computed in Step 2. These emission factors, specific to each fuel type, are applied to the energy distribution obtained in Step 4.3B for each scenario.

The methodology involves the following steps:

1. Emission Calculation by Fuel Type: We multiply the fuel-type-specific energy demands obtained in Step 4.2-3B by the respective emission factors derived in Step 2. This results in the total CO_2 emissions per fuel type for each country.

2. Summation by Country: The emissions from all fuel types are aggregated for each country, providing the total CO_2 emissions for 2050 under each scenario.

3. Segmentation into Training and Usage Emissions: The total CO_2 emissions are then split into emissions from training and usage (operation) based on predefined percentages.

4. Final Calculation of Emissions by Usage and Training: Using these percentages, we calculate:

- Emissions from usage (operation) as: **Total CO_2 × Usage Percentage**
- Emissions from training as: **Total CO_2 × (1 Usage Percentage)**

The total CO_2 emissions resulting from training and usage for each country in the Baseline, Fewer Larger, and More Smaller scenarios are presented in Tables 39, 40, and 41, respectively.

Table 39: Total CO_2 Emissions for 2050 - Baseline Scenario (kgCO_2)

Country	Usage Emissions (kgCO_2)	Training Emissions (kgCO_2)
Australia	3.25E+09	1.16E+10
Brazil	1.07E+08	3.79E+08
Canada	8.17E+08	2.90E+09
China	3.63E+09	1.29E+10
France	2.81E+09	9.98E+09
Germany	6.93E+09	2.46E+10
Hong Kong	2.82E+10	1.00E+11
Singapore	2.13E+10	7.57E+10
India	4.98E+10	1.77E+11
Indonesia	3.72E+09	1.32E+10
Japan	1.53E+10	5.45E+10
Netherlands	1.21E+09	4.29E+09
Spain	2.51E+09	8.91E+09
United Kingdom	5.72E+09	2.03E+10
United States	1.06E+12	3.77E+12

Table 40: Total CO_2 Emissions for 2050 - Fewer Larger Scenario (kgCO_2)

Country	Usage Emissions (kgCO_2)	Training Emissions (kgCO_2)
Australia	3.25E+09	5.42E+09
Brazil	1.07E+08	1.77E+08
Canada	8.17E+08	1.36E+09
China	3.63E+09	6.03E+09
France	2.81E+09	4.67E+09
Germany	6.93E+09	1.15E+10
Hong Kong	2.82E+10	4.70E+10
Singapore	2.13E+10	3.55E+10
India	4.98E+10	8.29E+10
Indonesia	3.72E+09	6.19E+09
Japan	1.53E+10	2.55E+10
Netherlands	1.21E+09	2.01E+09
Spain	2.51E+09	4.17E+09
United Kingdom	5.72E+09	9.52E+09
United States	1.06E+12	1.77E+12

Table 41: Total CO_2 Emissions for 2050 - More Smaller Scenario (kgCO_2)

**Country**	**Usage Emissions (kgCO_2)**	**Training Emissions (kgCO_2)**
Australia	3.25E+09	1.95E+10
Brazil	1.07E+08	6.37E+08
Canada	8.17E+08	4.88E+09
China	3.63E+09	2.17E+10
France	2.81E+09	1.68E+10
Germany	6.93E+09	4.15E+10
Hong Kong	2.82E+10	1.69E+11
Singapore	2.13E+10	1.27E+11
India	4.98E+10	2.98E+11
Indonesia	3.72E+09	2.22E+10
Japan	1.53E+10	9.17E+10
Netherlands	1.21E+09	7.21E+09
Spain	2.51E+09	1.50E+10
United Kingdom	5.72E+09	3.42E+10
United States	1.06E+12	6.34E+12

Hong Kong-Specific Emission Calculation

The methodology applied to estimate Hong Kong's CO_2 emissions in 2050 follows the same procedure as outlined in Table 38 for 2030. The emission assessment for 2050 maintains the same approach, incorporating both **imported nuclear energy** from Mainland China and **local electricity generation**, ensuring consistency with prior calculations.

The calculation steps remain unchanged:

1. The portion of energy demand met by imported nuclear power is multiplied by China's nuclear emission factor to determine the associated CO_2 emissions.
2. The remaining share of Hong Kong's energy demand is allocated across fuel types and multiplied by the corresponding emission factors, following the standard methodology.
3. The total CO_2 emissions are obtained by summing contributions from imported nuclear energy and local power generation.

This ensures a coherent and standardized methodology across different target years, allowing for comparative emissions analysis between 2030 and 2050 while maintaining consistency in energy allocation and carbon accounting frameworks.

8. Tables and Figures

This section outlines the step-by-step methodology used to estimate the carbon emissions resulting from AI-related energy demand. The framework consists of six key steps, each contributing to the systematic assessment of energy consumption and emissions at a global scale.

8.1 Energy Pricing

The first step involves determining the cost of energy per unit of electricity produced. This is achieved through a Multi-Regional Input-Output (MRIO) approach that accounts for the economic impact of energy generation across different countries. The process includes:

- Collecting energy price data by obtaining the total energy production cost per country and per energy source.
- Calculating the electricity cost per unit (TWh) by dividing the total production cost by the total electricity output.

This forms the foundation for subsequent emission calculations.

8.2 Emission Factor

Once the energy price per unit is established, emission factors are computed. This step quantifies the carbon footprint per TWh based on the Global Warming Potential (GWP) of different energy sources. The methodology includes:

- Identifying CO_2 emissions per energy source per country.

- Applying economic-based scaling factors to transition from cost-based emissions to energy-based emissions.
- Incorporating specific emission factors for Hong Kong and Singapore, given their distinct energy mix and import dependencies.

This step results in a country- and fuel-specific emission factor table used in later calculations.

8.3 Energy Demand

Energy demand is allocated based on the projected AI computational requirements. Given that data centers are concentrated in a limited number of countries, this step distributes the total energy demand according to the following principles:

- Selection of 15 countries, which together host 82.67% of global data centers, to represent worldwide energy demand.
- Assigning energy demand proportionally based on each country's data center capacity and expected AI workload growth.
- Differentiating between energy required for training AI models and operational (usage) energy.

8.4 Renewable and Non-Renewable Energy Split

To maintain consistency in emissions calculations, energy demand is further divided into renewable and non-renewable sources based on country-specific energy targets for 2030 and 2050. The key steps include:

- Distributing energy demand into specific fuel types, including coal, gas, oil (non-renewable), and solar, wind, hydro, nuclear (renewable).
- Maintaining each country's current energy mix ratios within the renewable and non-renewable categories.
- Ensuring that future energy projections align with national sustainability commitments.

This enables a structured approach to estimating the environmental impact of different AI deployment scenarios.

8.5 Energy Demand by Fuel Type

Using the renewable and non-renewable allocation, energy demand is assigned to individual fuel types within each category. This ensures consistency with observed energy consumption patterns while allowing for scenario-based adjustments. The methodology involves:

- Allocating non-renewable demand proportionally across coal, oil, and gas based on historical consumption shares.

- Allocating renewable demand across solar, wind, hydro, bioenergy, and nuclear according to existing national distributions.
- Applying this method to different AI deployment scenarios to observe variations in energy consumption profiles.

8.6 Total Carbon Footrpint

The final step involves computing total carbon emissions by multiplying fuel-specific energy demand with the corresponding emission factors. This yields country-level CO_2 emissions across all scenarios. The key steps include:

- Calculating CO_2 emissions per fuel type using previously derived emission factors.
- Aggregating emissions across fuel types for each country.
- Differentiating between emissions from training AI models and emissions from operational AI usage.

This approach ensures a rigorous and comprehensive assessment of the long-term carbon footprint of AI.

8.7 Flowchart Representation of the Methodology

To visually summarize the emission calculation framework, the following flowchart illustrates the sequential methodology:

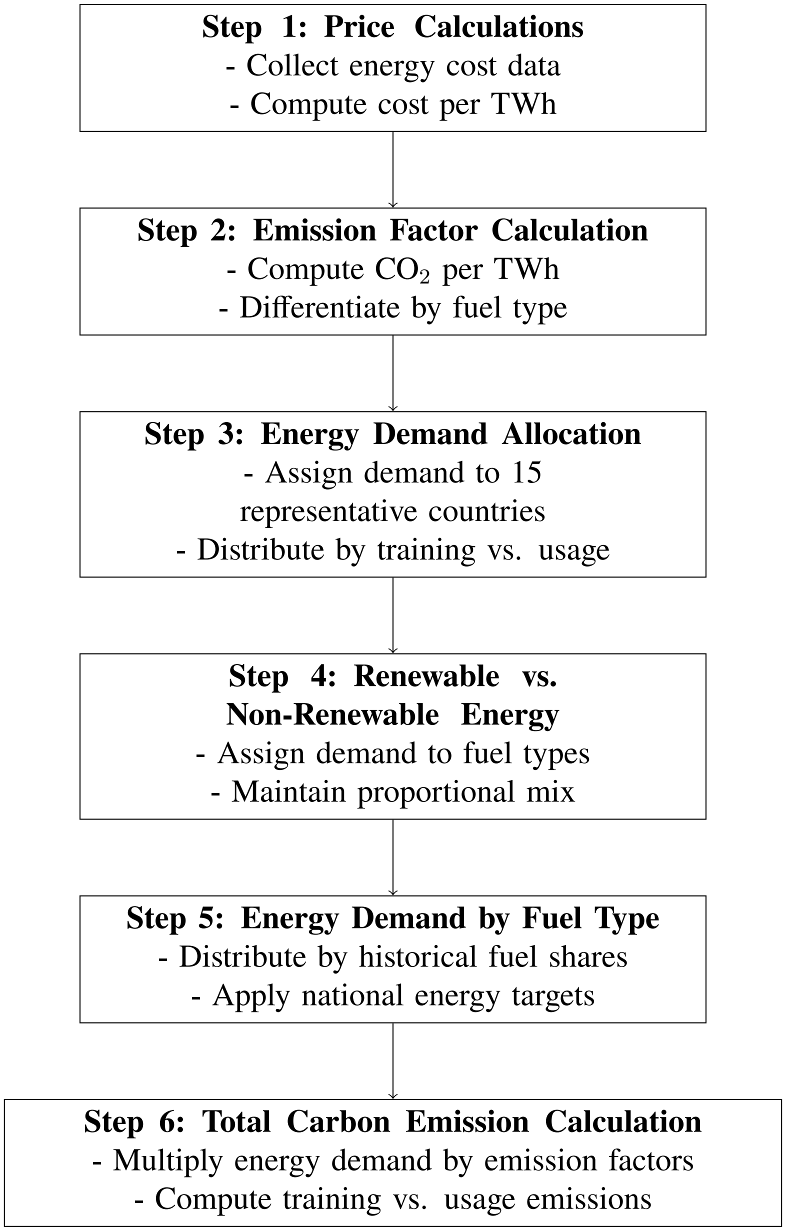

Figure 18: Flowchart representation of the emission calculation methodology.

This structured methodology provides a comprehensive framework for estimating AI-driven energy consumption and its associated carbon footprint, ensuring alignment with national energy targets and global sustainability goals.

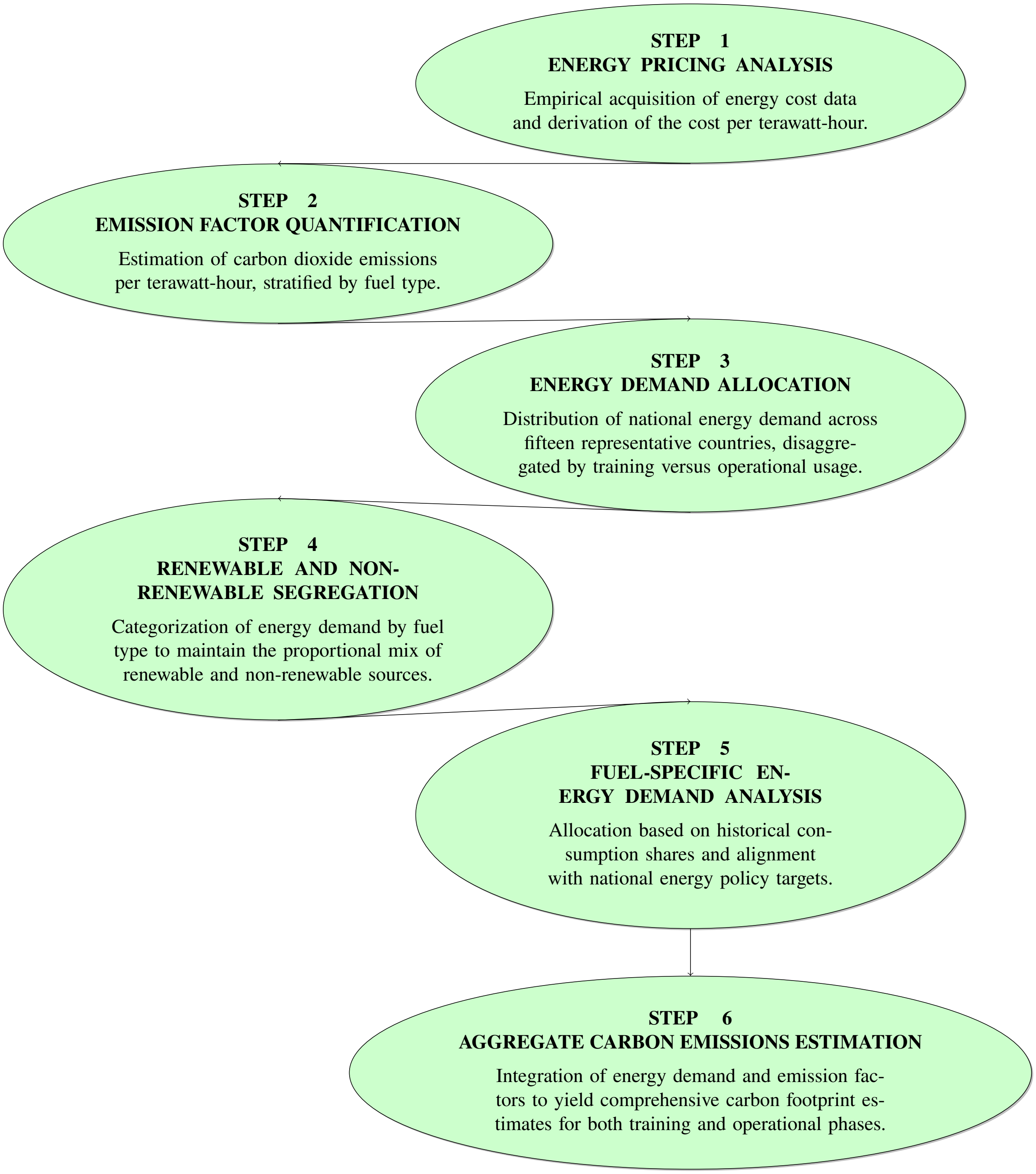

Figure 19: Flowchart representation of the emission calculation methodology.

9. Overall Combined CO_2 Emission Results

This section presents the total carbon emissions associated with different scenarios, providing a comparative assessment of the environmental impact across the Baseline, Fewer Larger, and More Smaller models. The analysis quantifies the carbon footprint resulting from electricity usage, model training, and supply chain processes, offering insights into the trade-offs between energy efficiency and computational demand.

The assessment consists of: 1. Training and Usage Emissions: Representing emissions generated from electricity consumption in data center operations and AI model training. 2. Manufacturing and Transportation Emissions: Accounting for the carbon footprint associated with hardware production, logistics, and supply chain activities. 3. Total Carbon Footprint: The summation of emissions from all sources, providing a holistic measure of the environmental implications of each scenario.

9.1 Scenario 1: Baseline

The emissions breakdown for the Baseline Scenario (Scenario 1) is presented in Table 42, illustrating the contributions of each source to the total carbon footprint in 2030 and 2050.

Table 42: Carbon Footprint (GT CO_2) by Source - Baseline Scenario

Year	Training	Usage	Manufacturing	Transportation	Total
2030	0.30	0.07	0.12	0.00	0.50
2050	4.29	1.21	2.79	0.03	8.31

9.2 Scenario 2: Fewer Larger Models

This scenario explores the impact of consolidating computational resources into fewer, more extensive models, thereby reducing overall training energy demand. The findings suggest that while training-related emissions are significantly lower than in the Baseline Scenario, operational energy use remains consistent. The total carbon footprint is notably reduced due to the efficiency gains associated with fewer computational processes. The emissions breakdown for this scenario is detailed in Table 43.

Table 43: Carbon Footprint (GT CO_2) by Source - Fewer Larger Models Scenario

Year	Training	Usage	Manufacturing	Transportation	Total
2030	0.19	0.07	0.08	0.00	0.34
2050	2.01	1.21	1.63	0.01	4.86

9.3 Scenario 3: More Smaller Models

In contrast, this scenario investigates the consequences of distributing computational workloads across a greater number of smaller models. While this approach enhances flexibility and scalability, it substantially increases the cumulative training energy requirements, leading to a higher total carbon footprint. The emissions assessment, as presented in Table 44, reveals that despite the distribution of workloads, the overall energy consumption and emissions surpass those observed in the Baseline Scenario.

Table 44: Carbon Footprint (GT CO_2) by Source - More Smaller Models Scenario

Year	Training	Usage	Manufacturing	Transportation	Total
2030	0.43	0.07	0.16	0.00	0.66
2050	7.21	1.21	4.28	0.13	12.83

Category Definitions: - Electricity (Training and Usage): Emissions derived from energy consumption related to AI model training and inference operations. - Supply Chain (Manufacturing and Transportation): Emissions resulting from hardware production, infrastructure maintenance, and distribution logistics.

These findings underscore the importance of optimizing computational strategies to balance efficiency and environmental sustainability in AI development.

10. Conclusion

This study systematically evaluates the carbon emissions associated with different AI model deployment scenarios, considering the energy consumption and environmental impact of data centers worldwide. The findings highlight the trade-offs between model size, operational efficiency, and sustainability, providing a comprehensive assessment of how different computational strategies influence global carbon footprints.

10.1 Scenario 1: Baseline

The Baseline Scenario represents a status quo model where energy consumption follows existing trends without significant optimizations. As illustrated in previous sections, this scenario results in moderate CO_2 emissions, with the highest contributions stemming from model training. While operational energy use remains stable, the total emissions escalate significantly by 2050 due to growing computational demands.

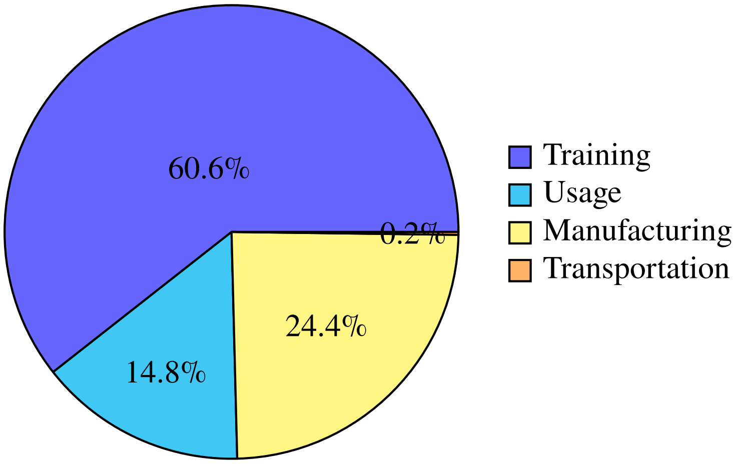

Figure 20: Carbon Emission Distribution - Baseline Scenario (2030)

10.2 Scenario 2: Fewer Larger Models

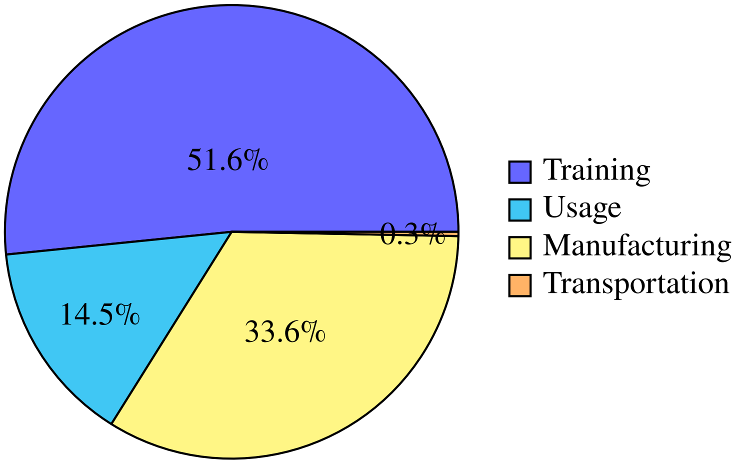

Figure 21: Carbon Emission Distribution - Baseline Scenario (2050)

The Fewer Larger Models Scenario investigates the impact of consolidating computational workloads into fewer but more extensive AI models.

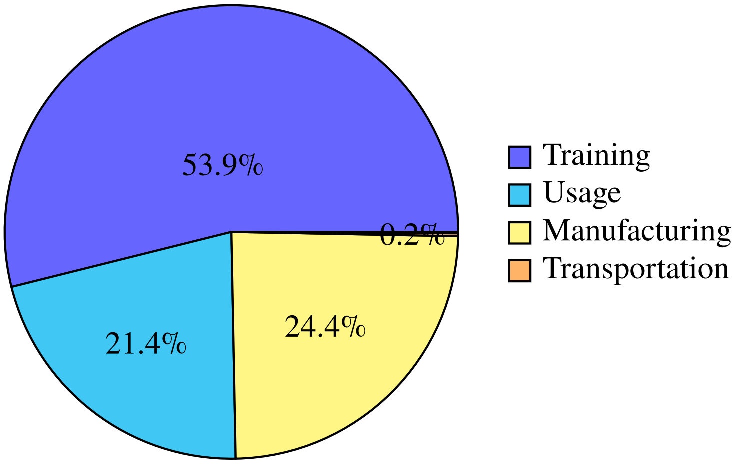

Figure 22: Carbon Emission Distribution - Fewer Larger Models (2030)

10.3 Scenario 3: More Smaller Models

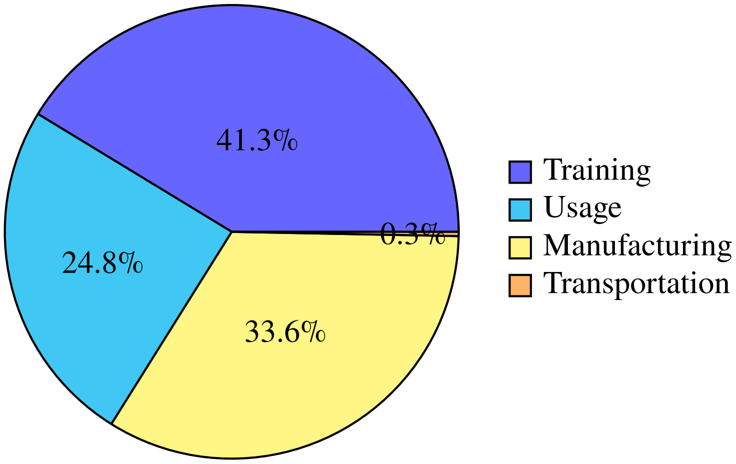

Figure 23: Carbon Emission Distribution - Fewer Larger Models (2050)

The More Smaller Models Scenario examines the implications of distributing computational processes across a greater number of smaller AI models.

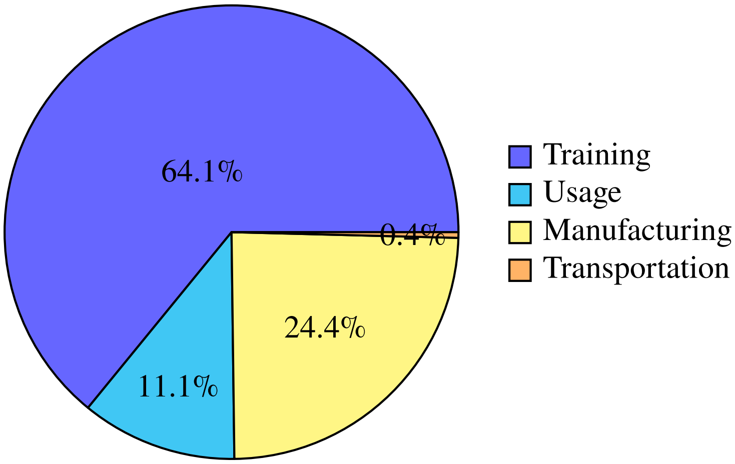

Figure 24: Carbon Emission Distribution - More Smaller Models (2030)

10.4 Final Considerations

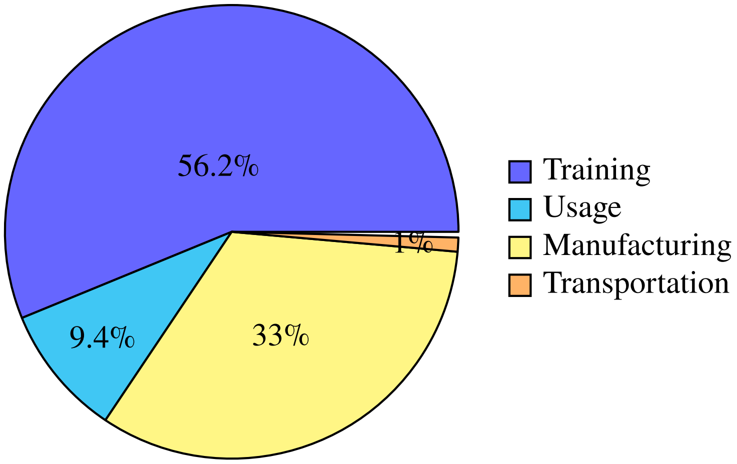

Figure 25: Carbon Emission Distribution - More Smaller Models (2050)

The comparative analysis of these three scenarios underscores the importance of energy-efficient AI development strategies.

11. Business As Usual Results and Discussion

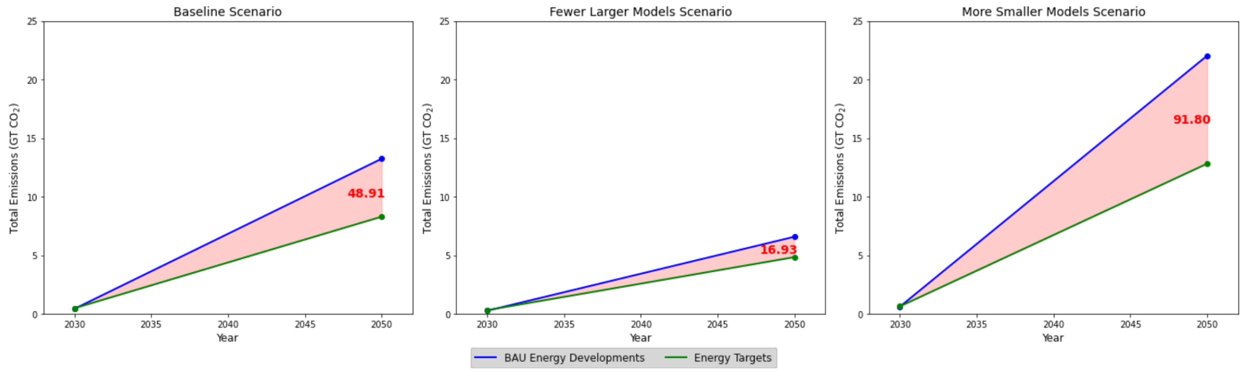

Figure 26: **Line Graph Comparison of Scenarios (2030–2050)** This figure illustrates total emissions under Business as Usual (blue line) and Energy Targets (green line) scenarios for the years 2030 and 2050. Red dashed lines highlight the difference gap between the two trajectories at evenly spaced intervals. The slope of each line reflects the projected growth in carbon emissions, emphasizing the divergence in cumulative impact over time.

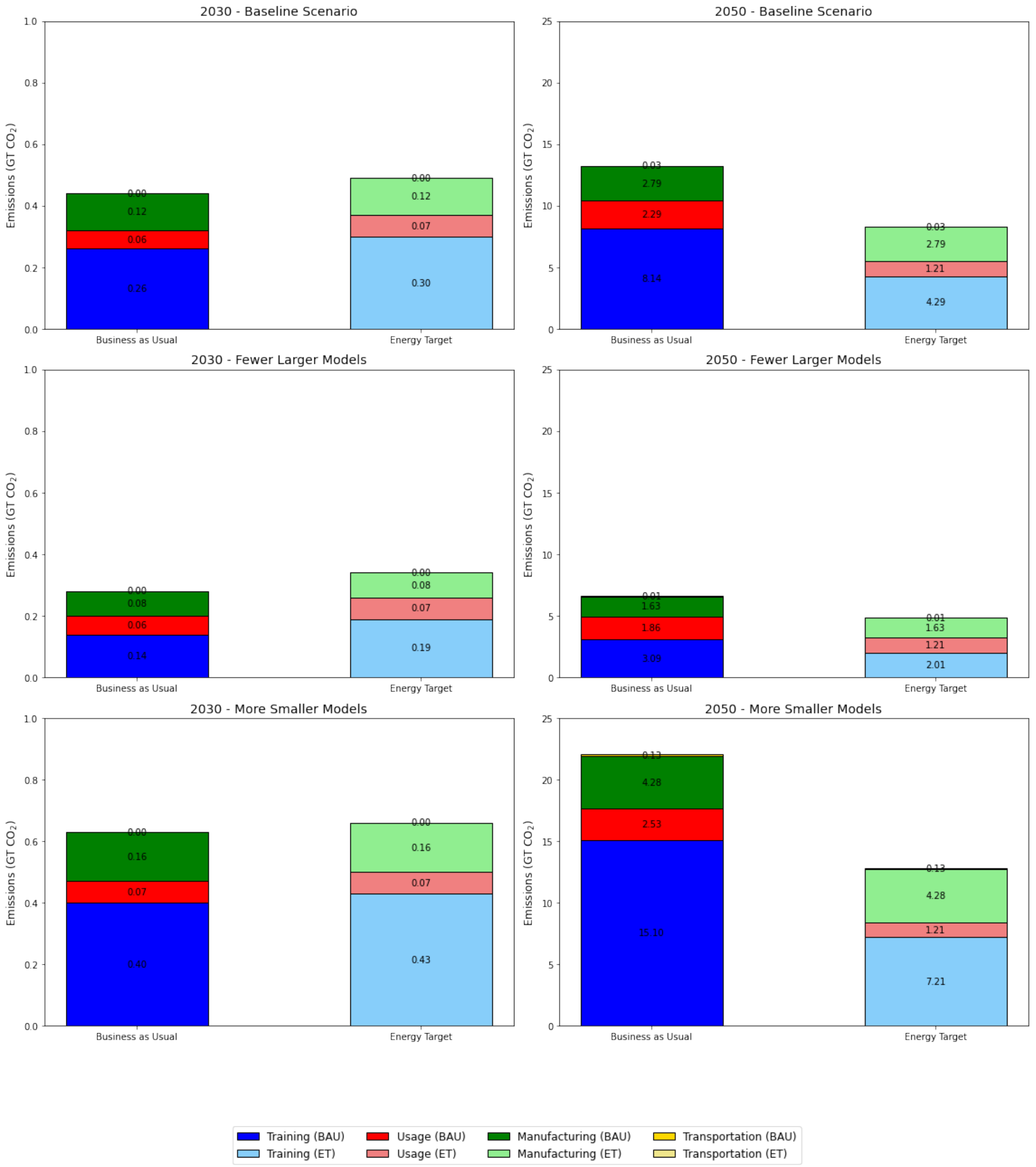

Figure 27: **Stacked Bar Charts for 2030 and 2050 Across Three Models** The six-panel layout contrasts three modeling approaches (Baseline, Fewer Larger Models, and More Smaller Models) over two time frames (2030 on the left, 2050 on the right). Each bar is divided into Training, Usage, Manufacturing, and Transportation components, allowing a granular view of the sources of emissions within each scenario. This side-by-side comparison highlights how shifts in model configurations can alter the distribution and magnitude of emissions over the projected period.

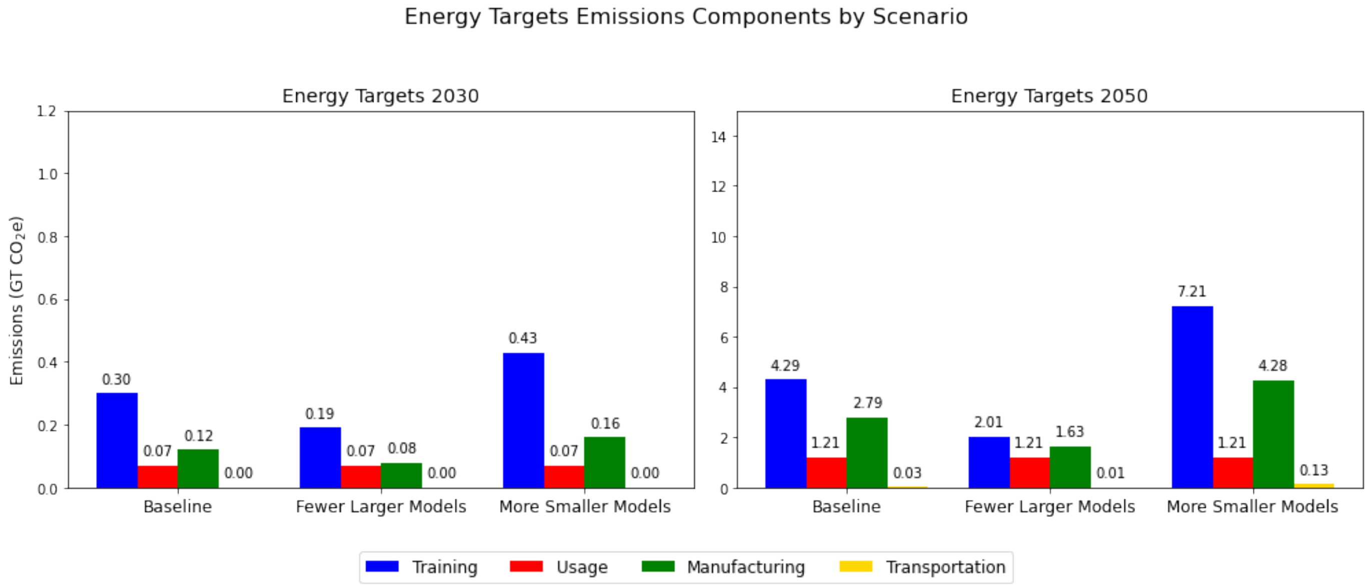

Figure 28: **Energy Targets Emissions Components by Scenario (2030 and 2050)** These dual panels focus on Energy Targets exclusively, providing a clearer view of how carbon emissions break down across the Training, Usage, Manufacturing, and Transportation components under different model configurations. The left panel represents 2030, while the right panel depicts 2050. Each bar cluster corresponds to a specific scenario (Baseline, Fewer Larger Models, More Smaller Models), revealing variations in the proportional contribution of each component.

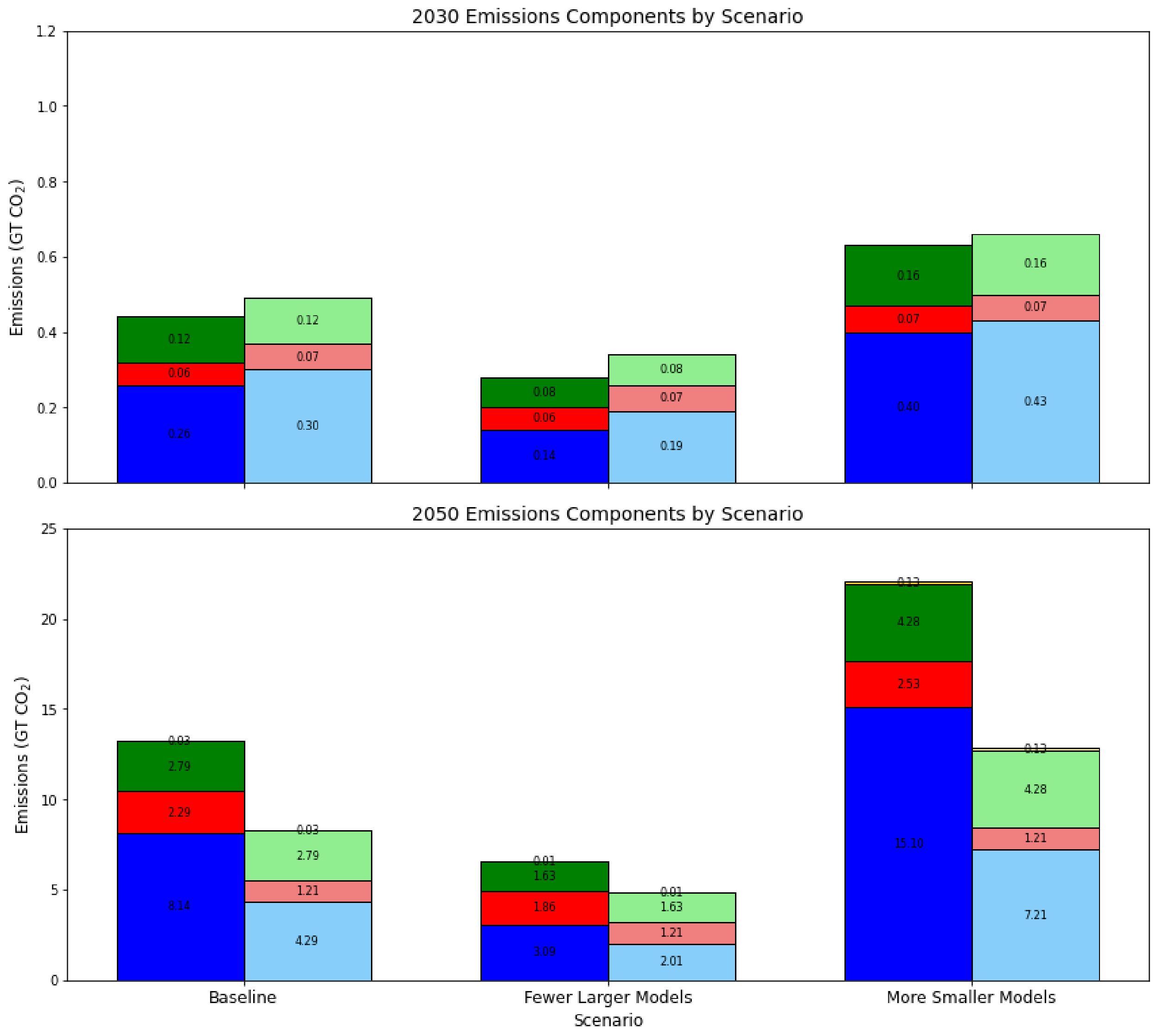

Figure 29: **Comparison of 2030 and 2050 Emissions by Scenario** The top chart presents stacked bars for 2030, and the bottom chart for 2050. Each scenario (Baseline, Fewer Larger Models, More Smaller Models) is represented along the x-axis, with Training, Usage, Manufacturing, and Transportation emissions stacked within each bar. Color coding highlights the relative contributions of each component. The increase in overall bar heights from 2030 to 2050 underscores the anticipated rise in total emissions unless more stringent mitigation measures are adopted.

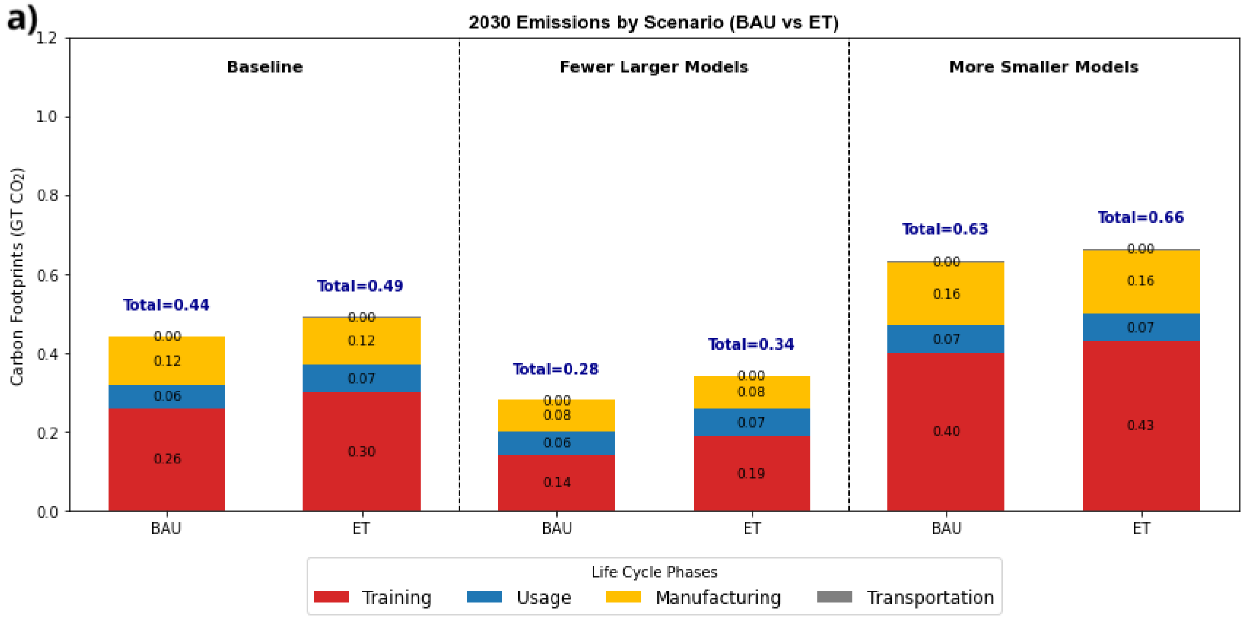

Figure 30: **Stacked Bar Charts for 2030 Across Three Model Scenarios**

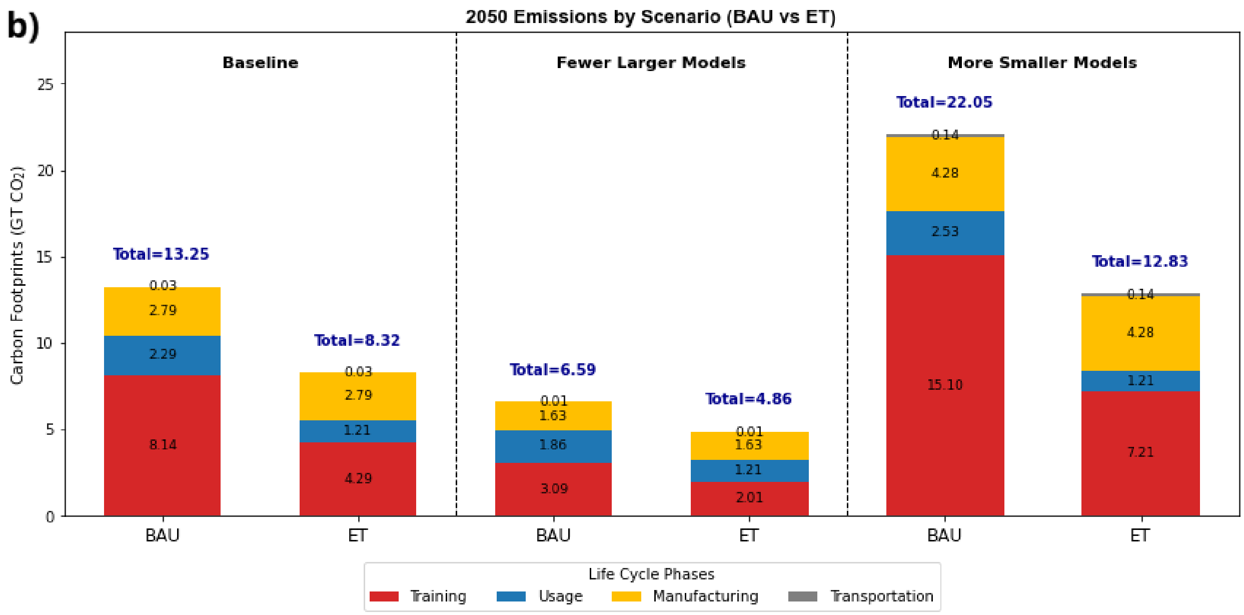

Figure 31: **Stacked Bar Charts for 2050 Across Three Model Scenarios**

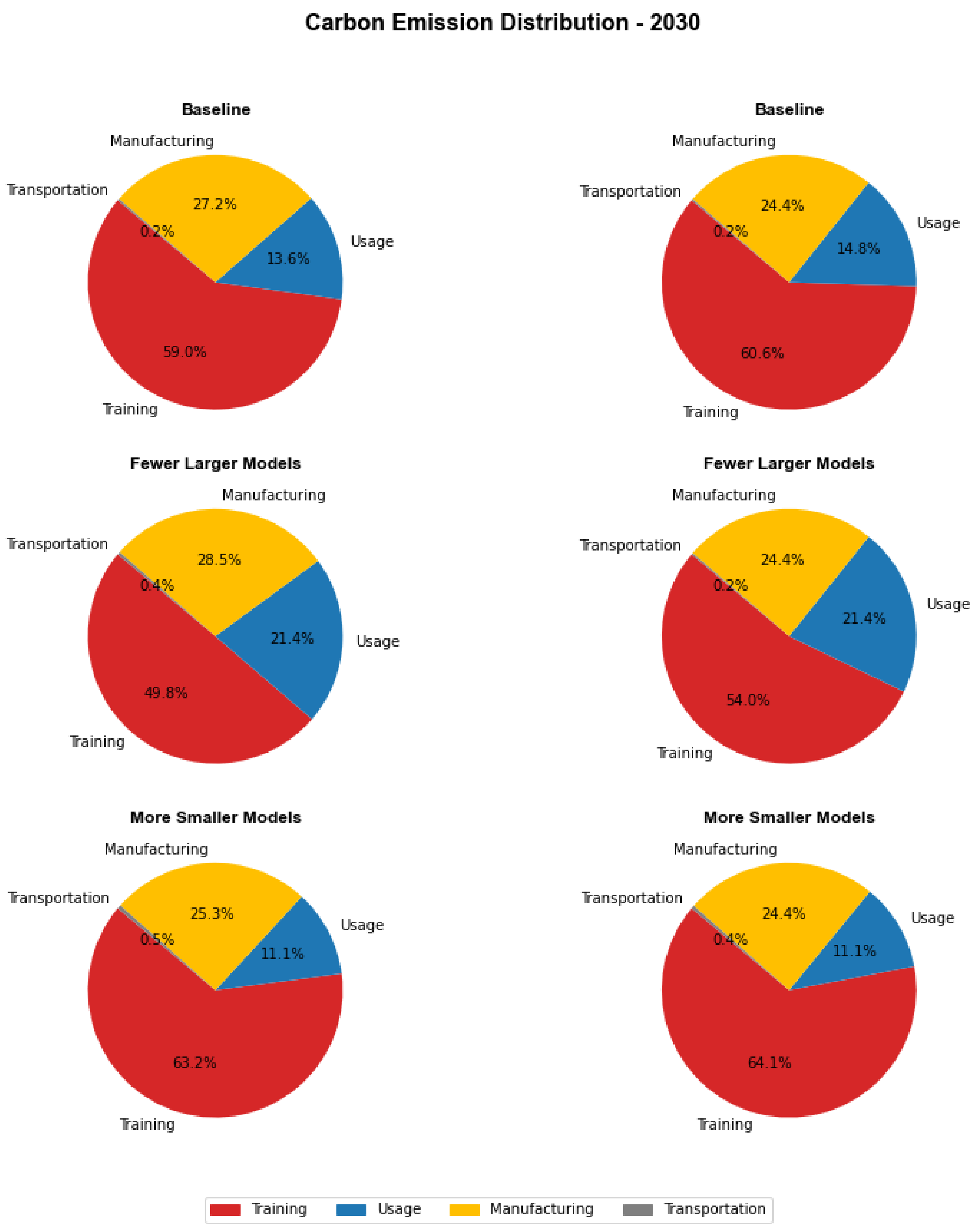

Figure 32: **Pie Charts for 2030 Across Three Model Scenarios**

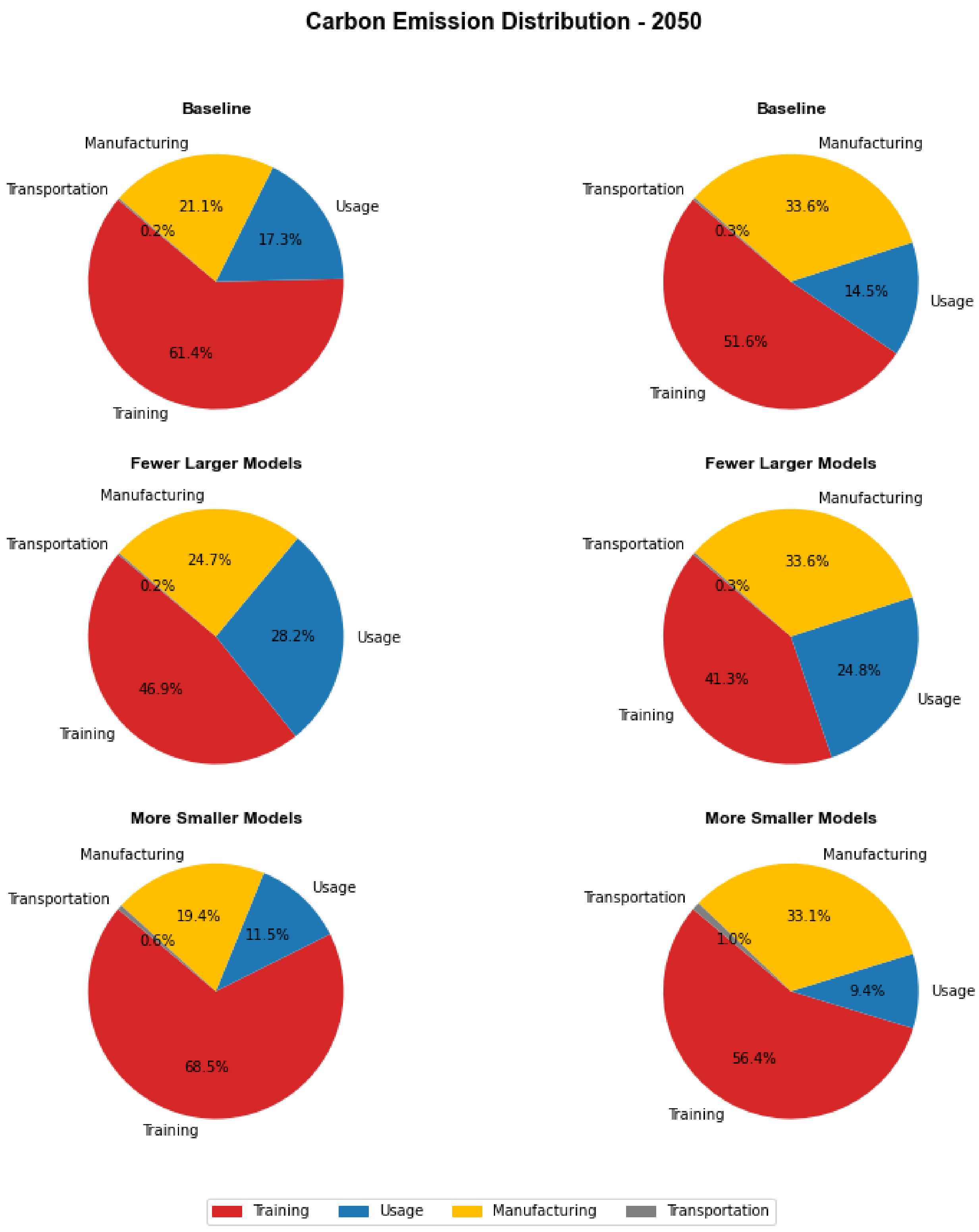

Figure 33: **Pie Charts for 2050 Across Three Model Scenarios**

A. Appendix

Country	Sector	Percentage
TW	Production of electricity by coal	17.14866528
TW	Production of electricity by gas	6.22883726
TW	Manufacture of gas; distribution of gaseous fuels through mains	4.358052026
TW	Manufacture of electrical machinery and apparatus n.e.c. (31)	4.123166482
TW	Casting of metals	3.25854409
TW	Plastics	2.862793409
CN	Production of electricity by coal	1.857037432
TW	Manufacture of basic iron and steel and of ferro-alloys and first products thereof	1.496369643
TW	Manufacture of coke oven products	1.340061334
TW	Chemicals nec	1.296749318
ID	Copper production	1.210706009
CN	Manufacture of basic iron and steel and of ferro-alloys and first products thereof	1.064758361
TW	Recycling of waste and scrap	0.995908569
KR	Production of electricity by coal	0.917652202
TW	Production of electricity by petroleum and other oil derivatives	0.900922541
TW	Mining of lead	0.865495809
IN	Mining of coal and lignite; extraction of peat (10)	0.850550857
WA	Production of electricity by coal	0.844421295
JP	Production of electricity by coal	0.79343894
TW	Manufacture of other transport equipment (35)	0.790799783
**Sum of first 20 affected sectors by Taiwan manufacture of electrical machinery and apparatus**		**53.20493064**
**Others**		**46.79506936**

Table 45: The percentage distribution of the contribution of production activities within Taiwan's manufacture of electrical machinery and apparatus, alongside the utilization patterns in other countries and their respective sectors, to CO_2 emissions, adjusted according to their Global Warming Potential (GWP) percentages.

References

[1] Strubell, E., Ganesh, A., & McCallum, A. (2020). Energy and policy considerations for modern deep learning research. *Proceedings of the AAAI Conference on Artificial Intelligence*, *34*(09), 13693–13696.

[2] Patterson, D., Gonzalez, J., Le, Q. V., Liang, C., Munguia, L. M., Rothchild, D., ... & Dean, J. (2021). Carbon emissions and large neural network training. *arXiv preprint arXiv:2104.10350*. https://arxiv.org/abs/2104.10350

[3] AnandTech. (2012). NVIDIA GeForce GTX 680 review. https://www.anandtech.com/show/5699/nvidia-geforce-gtx-680-review/19

[4] TechPowerUp. (2017). NVIDIA GeForce GTX 1080 specifications. https://www.techpowerup.com/gpu-specs/geforce-gtx-1080.c2839

[5] TechPowerUp. (2020). NVIDIA GeForce RTX 3080 specifications. https://www.techpowerup.com/gpu-specs/geforce-rtx-3080.c3621

[6] Fischetti, M., & Lodi, A. (2018). Mixed-integer programming in computational optimization. *Annals of Operations Research*, *271*(1), 1–16. https://doi.org/10.1007/s10479-017-2652-0

[7] Gurobi Optimization, LLC. (2023). MILP performance evolution. https://www.gurobi.com/features/gurobi-optimizer-delivers-unmatched-performance/

[8] NVIDIA. (2023). Sustainable computing: Power efficiency and cooling technologies. https://resources.nvidia.com/en-us-sustainable-computing/grace-cpu

[9] Sevilla, J., Heim, L., Ho, A., Besiroglu, T., Bowman, R., & Stein-Perlman, M. (2022). Compute trends across three eras of machine learning. *arXiv preprint arXiv:2202.05924*. https://arxiv.org/abs/2202.05924

[10] Bender, E. M., Gebru, T., McMillan-Major, A., & Shmitchell, S. (2021). On the dangers of stochastic parrots: Can language models be too big? *Proceedings of the 2021 ACM Conference on Fairness, Accountability, and Transparency (FAccT)*, 610–623. https://doi.org/10.1145/3442188.3445922

[11] Thompson, A. D. (2024). Models table. *LifeArchitect.ai*. https://lifearchitect.ai/models-table/

[12] United Nations Development Programme. (2023). Human development index and its components. https://hdr.undp.org/data-center/human-development-index

[13] World Bank. (2024). The digital divide: Bridging global disparities in ICT access. https://www.worldbank.org/en/topic/digitaldevelopment

[14] OECD. (2023). Artificial intelligence in the global economy. *OECD Digital Economy Papers*. https://www.oecd.org/digital/ai/

[15] International Energy Agency. (2023). China's energy transition and AI growth. https://www.iea.org/reports/china-energy-outlook

[16] International Monetary Fund. (2024). Emerging markets and AI adoption. *IMF Working Paper*. https://www.imf.org/en/Publications/WP

[17] United Nations. (2023). ICT infrastructure in developing nations: Challenges and opportunities. https://www.un.org/en/development/ICT

[18] World Health Organization. (2024). Climate change and technological inequalities. https://www.who.int/publications/climate-impact

[19] World Bank. (2024). World development indicators: Population growth by country. https://databank.worldbank.org/source/world-development-indicators

[20] International Energy Agency. (2024). Global energy demand report. https://www.iea.org/reports/global-energy-demand

[21] UK Government. (2018). *Supplementary note: Biomass power and GHG sensitivity*. https://assets.publishing.service.gov.uk/media/5a79e6b6ed915d042206bdb6/7329-supplementary-note-biomass-power-and-ghg-sensitiv.pdf

[22] Renewable Energy Journal. (2003). *Emission factors study*. https://www.sciencedirect.com/science/article/pii/S0960148103002519

[23] Solaris Renewables. (2021). *What is the carbon footprint of solar panel manufacturing?* https://solarisrenewables.com/blog/what-is-the-carbon-footprint-of-solar-panel-manufacturing

[24] World Nuclear Association. (2022). *Carbon dioxide emissions from electricity*. https://world-nuclear.org/information-library/energy-and-the-environment/carbon-dioxide-emissions-from-electricity

[25] Our World in Data. (2021). *Electricity mix*. https://ourworldindata.org/electricity-mix

[26] World Energy Council. (2021). *Hong Kong energy profile*. https://www.worldenergy.org/assets/downloads/Hong_Kong%2C_China.pdf

[27] Hong Kong Government Census and Statistics Department. (2022). *Hong Kong energy statistics annual report 2022*. https://www.censtatd.gov.hk/en/Energy_Statistics_Annual_Report

[28] Onat, N. C., Mandouri, J., Kucukvar, M., et al. (2023). Rebound effects undermine carbon footprint reduction potential of autonomous electric vehicles. *Nature Communications*, *14*, 6258. https://doi.org/10.1038/s41467-023-41992-2

[29] HPCwire. (2023, August 17). Nvidia H100: Are 550,000 GPUs enough for this year? Retrieved April 6, 2025, from https://www.hpcwire.com/2023/08/17/nvidia-h100-are-550000-gpus-enough-for-this-year/

[30] Freightos. (2022, May 12). Freightos launches carbon calculator to help importers and exporters monitor and cut supply chain emissions. Retrieved April 6, 2025, from https://www.freightos.com/press-release/freightos-launches-carbon-calculator-to-help-importers-and-exporters-monitor-and-
