# Supplementary material for: Scenario-based forecasting of the global energy demand and carbon footprint of artificial intelligence
Source: PLoS One. 2026 Mar 11;21(3):e0343056. doi: 10.1371/journal.pone.0343056 (PMC12978478; doi:10.1371/journal.pone.0343056)
Supplement: Attachment — Submitted filename: Response_to_Reviewers.pdf [file pone.0343056.s003.pdf]

Manuscript Number: PONE-D-25-38245

Title: Scenario-based forecastng of the global energy demand and carbon footprint of artificial intelligence

We thank the reviewers for their careful reading of the manuscript and for their constructive comments. We have made the appropriate changes to formatting, including using world maps in the public domain. Below, we respond to each of the reviewer's comments point by point.

Reviewer #1: I recommend publication in PLOS ONE after major revision based on the following comments.
1. The reason for the selection of digital infrastructure sustainability must be added.

The revision clarifies that digital infrastructure, specifically data centers, is the focal point because it is the primary source of energy consumption and carbon emissions associated with AI systems (please see page 1, the second paragraph of the introduction).

2. The introduction is too verbose; special effort is needed to trip the contents without jeopardizing the message.

The introduction has been restructured to reduce redundancy and improve focus, while preserving the necessary contextual grounding for a global, long-horizon systems analysis.

3. The authors should highlight the effect of scenario-based energy forecasting in carbon intensity of computation.

This effect is now explicitly emphasized. The revised manuscript demonstrates quantitatively that deployment structure and electricity mix assumptions can dominate efficiency gains in determining the carbon intensity of computation, a central result illustrated in the scenario comparisons (please see page 3, the final paragraph of the introduction).

4. The authors need to give more information about the research significance.

The revised introduction explicitly identifies the knowledge gap addressed by this study: the absence of an integrated framework that jointly models global AI deployment trajectories, evolving electricity systems, and supply-chain emissions. The novelty lies in

coupling scenario-based computational demand modeling with MRIO-based life-cycle carbon accounting, rather than in global scope alone.

5. The role of artificial intelligence systems in the carbon intensity of computation should be clarified.

The revised text clarifies that AI systems influence carbon intensity by determining where, when, and at what scale electricity is consumed, through choices in model architecture, deployment patterns, and reliance on centralized versus distributed infrastructure (please see page 2, the fourth paragraph of the introduction).

6. Some important papers about sustainable energy (such as doi.org/10.1039/D5EE02029H) can be cited.

Relevant sustainable energy systems literature, including the suggested article, has been incorporated into the revised manuscript. The new citations include:

- Sanati et al. (2025), which informs the discussion of energy system efficiency improvements and low-carbon innovation pathways relevant to long-run decarbonization scenarios.
- Yang et al. (2025), which supports the treatment of embodied renewable energy and global trade linkages within the multi-regional input–output framework.
- Lu and Chen (2025), which provides methodological grounding for the spatially disaggregated MRIO modeling of power sector emissions used in the regional analysis.
- Faturay et al. (2020), which contextualizes the application of MRIO models to energy system transitions and supports the treatment of upstream economic and energy impacts.

7. In the conclusions part, only some conclusions were presented, beyond that, more perspectives are suggested to be provided.

The Conclusion has been revised to more clearly synthesize the study’s core findings and their implications, rather than focusing primarily on methodological limitations. The updated text highlights that AI’s long-term energy use and emissions are governed by the interaction between deployment structure, electricity system decarbonization, and the geographic concentration of compute, with efficiency improvements alone proving insufficient. It now summarizes key contrasts across scenarios, emphasizing the comparatively larger emissions reductions achievable through structural interventions such as model consolidation and low-carbon electricity transitions. Remaining uncertainties and data limitations inherent to long-horizon scenario analysis are retained but more directly linked to the interpretation of results, and the exploratory (non-predictive) nature of the scenarios is clarified alongside the resulting policy relevance.

Reviewer #2: Title
Unspecified, “Scenario-based forecasting of the global energy demand and carbon footprint of AI” fails to indicate the methodological novelty (e.g., MRIO integration, six-scenario modeling) or specific time horizon.
Does not distinguish whether the analysis is empirical, simulation-based, or theoretical, reducing clarity for readers searching for methodological studies.
Please add study design.

The revised abstract explicitly characterizes the study as a “scenario-based, simulation-driven modeling framework” that links mathematical representations of computational demand with life-cycle carbon accounting. This clearly distinguishes the work from purely empirical or theoretical studies.

Abstract
Missing essential methodological transparency (e.g., model validation, uncertainty quantification).
No clear mention of data sources or the calibration framework for efficiency parameters.
Policy implications are asserted (“efficiency gains alone will not suffice”) but not supported by a quantitative comparison in the abstract.
Sentence structure is long and complex, affecting readability.
Keywords: Many and lacking prioritization. Some are descriptive rather than functional (“technology adoption” is unclear).
Missing terms that reflect the study’s modeling approach (e.g., “scenario analysis,” “sustainability modeling,” “AI energy systems”).

The revised abstract now includes explicit quantitative comparisons. In particular, it reports that deployment and electricity-mix scenarios can reduce total emissions by up to 40% relative to business-as-usual pathways, exceeding the reductions achievable through efficiency gains alone by more than 20 percentage points. These figures directly support the stated policy implications.

The keyword list has been revised to prioritize methodological and analytical content. Terms such as scenario analysis, electricity demand forecasting, AI energy systems, and multi-regional input-output analysis have been added, while overly generic descriptors have been removed.

Introduction
Lengthy and redundant. The background has many pages and repeats data center energy statistics.

The introduction has been restructured to reduce redundancy and improve focus, while preserving the necessary contextual grounding for a global, long-horizon systems analysis.

Limited critical synthesis of prior scenario-based energy studies or integrated assessment models (IAMs); references 5-8 are not analyzed comparatively.

The cited references were not intended to represent a set of comparable scenario-based or IAM studies. Rather than synthesizing existing IAM projections, our objective is to introduce a complementary, infrastructure-focused modeling framework that links AI deployment to data-center electricity demand and upstream supply chains. The cited works therefore serve to motivate the problem space and highlight gaps in existing assessments, which the proposed MRIO-based approach is designed to address.

The knowledge gap is not articulated; it is unclear whether the novelty lies in global scope, scenario design, or MRIO coupling.

The revised introduction explicitly identifies the knowledge gap addressed by this study: the absence of an integrated framework that jointly models global AI deployment trajectories, evolving electricity systems, and supply-chain emissions. The novelty lies in coupling scenario-based computational demand modeling with MRIO-based life-cycle carbon accounting, rather than in global scope alone.

Unsupported generalizations, such as “AI currently represents approximately 0.4% of global electricity consumption,” are presented without citing precise datasets or year references.

This estimate is now explicitly anchored to the year 2024 and supported by Shehabi, Smith, and Masanet’s paper published by the Lawrence Berkeley National Laboratory (see page 1, second paragraph of the introduction). The temporal and empirical basis of the figure is clearly stated.

No clear conceptual framework figure to orient the reader before reading the “Methods.”

A detailed conceptual and methodological framework is already provided as a comprehensive figure at the beginning of the Methods section, where it can be interpreted alongside the formal model definitions. We intentionally avoided introducing an additional high-level schematic earlier in the manuscript to prevent redundancy and ensure that the framework is presented with sufficient technical context.

Materials and Methods

Model Framework
The structure (Fig. 1) is descriptive but there are no validation steps or uncertainty propagation techniques (e.g., Monte Carlo or sensitivity analysis).

Figure 1 is intended to present the conceptual and modular structure of the modeling framework rather than to serve as a validation or uncertainty analysis figure. Validation in the conventional predictive sense is not feasible in a long-horizon, scenario-based assessment, as the model is not designed to forecast realizations but to bound plausible futures conditional on structural assumptions. Uncertainty is instead incorporated through explicit scenario design and parametric ranges calibrated from the literature, with resulting dispersion reported in downstream figures. We have clarified this distinction in the revised manuscript and explicitly state that the framework is exploratory and comparative rather than predictive. A discussion of uncertainty sources and their propagation through scenario spread has also been expanded in the Methods and Discussion sections.

No justification for selecting exponential and logistic functional forms for model scaling and efficiency gains.

The justification for the use of exponential and logistic functional forms has been made explicit in the Methods section, primarily in Sections “Energy efficiency” and “Computational demand.” These sections now explain that decelerating exponential forms are used to represent empirically observed patterns of rapid early improvement followed by diminishing returns in hardware, algorithmic, and infrastructure efficiency, while logistic diffusion is employed to capture saturation effects in AI adoption. The text clarifies that these functions are used as stylized bounds for long-run scenario analysis rather than as short-term predictive models.

Energy Efficiency Modeling
Equations (1) to (4) are complex but empirical parameters are not justified nor referenced.

All empirical parameters are now explicitly documented and referenced in the Methods section at first use, with detailed justification provided in the Supplementary Sections 2.1-2.3 (efficiency parameters) and 3.1–3.4 (computational demand parameters). These supplementary sections describe data sources, calibration procedures, parameter ranges, and sensitivity bounds, and are cross-referenced in the main text to ensure transparency and reproducibility.

Independence assumptions among hardware, algorithmic, and infrastructural efficiencies (Eq. 4) are strong and unsupported.

Independence among hardware, algorithmic, and infrastructural efficiency components is a standard first-order modeling assumption adopted for transparency and tractability.

The limitation of this assumption is explicitly acknowledged, and relaxing it would require data that are currently unavailable at a global scale.

No validation using real-world benchmarks (e.g., GPU generation trends or data center efficiency reports).

The model is calibrated against historical GPU efficiency trends and reported data center PUE improvements. Long-horizon validation against future outcomes is not feasible for scenario-based projections through 2050. The purpose of the framework is comparative scenario analysis rather than predictive validation.

Computational Demand
Depends on compound exponential extrapolation of model complexity and query volume.

The use of multiple contrasting scenarios is precisely intended to bound realistic growth trajectories rather than assert a single extrapolation. Scenario-based modeling is employed to explore structural sensitivities, not to claim deterministic forecasts.

Population and adoption rates (Eq. 10) use logistic diffusion but no region-specific calibration is shown.

Regional heterogeneity is incorporated through stratification by inequality-adjusted human development index (IHDI), which serves as a transparent and globally available proxy (see Supplementary Section 3.3.1). Country-specific digital infrastructure calibration is incorporated by accounting for the number of data centers, as outlined in the Supplementary Section 7.2.1.

Data sources unclear (Supplementary Sections cited but not summarized in text).
MRIO Analysis

Response:

We thank the reviewer for this comment and agree that clarity regarding data sources is important for transparency and reproducibility. In the revised manuscript, we have added a concise summary statement to the *Multi-Regional Input-Output Model* section explicitly outlining the key data inputs used in the MRIO framework (including electricity production values, emission factors, price tables, and country-specific adjustments), while retaining detailed numerical tables and sectoral mappings in the Supplementary Information. This ensures that readers can readily identify the provenance and structure of the data directly from the main text without consulting supplementary materials.

Action:

A one-sentence summary of MRIO data sources has been added to Section 7.1.2.

The MRIO methodology is standard, but no sensitivity testing or temporal updating of EXIOBASE is described.

Response: In this study, the MRIO framework is not used as a forecasting or scenario-generating model, but rather as a carbon accounting layer that translates scenario-driven energy demand trajectories into associated life-cycle emissions.

Uncertainty and sensitivity in this study are addressed primarily through the design of the scenario framework rather than through sensitivity analysis at the MRIO calculations. The scenarios systematically vary the key drivers of AI-related energy demand and emissions, including regional AI adoption rates and diffusion pathways, alternative model scaling configurations (Baseline, Fewer–Larger, and More–Smaller), projected trajectories of hardware and algorithmic efficiency improvements, and contrasting electricity system assumptions under Business-as-Usual and Energy Target pathways. By exploring these dimensions in combination, the scenario framework captures the dominant sources of uncertainty influencing long-term AI energy demand and associated carbon emissions in a transparent and policy-relevant manner.

These dimensions constitute the primary analytical focus of the paper. Conducting additional MRIO-level sensitivity analysis would therefore be methodologically redundant and risk conflating accounting uncertainty with scenario uncertainty, which the framework deliberately separates.

Regarding temporal updating, the analysis employs EXIOBASE 3.8.2, the most recent publicly available release, reflecting contemporary global production technologies and trade structures. In this study, MRIO coefficients are applied as time-invariant emission intensities to scenario-specific energy demand, rather than being endogenously projected over time. As such, temporal interpolation or extrapolation of MRIO tables would not meaningfully improve the robustness of results and would introduce additional, largely unverifiable assumptions about future global supply-chain restructuring.

Moreover, for key sectors driving results, most notably electricity generation, the emission intensities used in EXIOBASE are grounded in physical and thermodynamic characteristics (e.g., fuel carbon content and conversion efficiencies). These electricity generation sectors' direct emissions are mostly above 90%. While power-system decarbonization is explicitly captured through scenario-specific electricity mixes, the underlying MRIO sectoral emission coefficients (e.g., electricity from natural gas) are

relatively stable over time and do not represent a major source of uncertainty compared to demand growth and energy-system transformation assumptions.

Taken together, this modeling choice ensures internal consistency, transparency, and policy relevance, while avoiding over-interpretation of MRIO uncertainty in a context where scenario uncertainty is the dominant analytical concern.

Attribution of GPU manufacturing entirely to Taiwan is over-simplified; ignores TSMC Arizona, Samsung Texas, or emerging EU fabs.

Response:

The reviewer's concern is understandable from a forward-looking capacity announcement perspective; however, the attribution in this study is based on realized production dominance, not planned or speculative capacity.

Within the relevant time horizon and technology scope considered in this study, advanced-node semiconductor fabrication for high-performance GPUs remains overwhelmingly concentrated in Taiwan. In particular, industry analyses indicate that more than 90% of global production capacity at the 3 nm node is concentrated in Taiwan, notwithstanding ongoing investments in fabrication facilities in the United States and Europe.

Facilities such as TSMC Arizona and Samsung Texas are explicitly acknowledged in the manuscript as emerging and strategically important. However, their present output does not yet constitute a material share of global advanced GPU manufacturing. Allocating significant production shares to these facilities at this stage would therefore introduce forward-looking assumptions not supported by realized production data. Accordingly, attributing GPU manufacturing emissions to Taiwan reflects current global semiconductor supply-chain realities, rather than a permanent or normative assumption about future industrial geography.

Carbon Footprint Estimation
Equations (15) to (21) are mathematically correct but no validation against known industry LCA benchmarks (e.g., ISO 14040 standards).

Response:

We acknowledge that validation against ISO 14040/44 standards is essential for product-level life-cycle assessments with defined functional units. ISO 14040/44 does not require “validation” in the sense of experimental verification. It requires methodological conformity and internal consistency across four phases. In this sense, the study follows the four-phase LCA framework, with a clearly defined goal and scope, a consistent functional unit, and transparently documented system boundaries. However, It should be noted that this study’s focus is not to provide a product level LCA for a GPU (e.g. H100). This could be a stand-alone study with a different scope. Our study does not conduct a process level LCA or device-specific LCA of GPUs or AI systems. While a more detailed estimations using process level LCA could be helpful for our main estimation model, it doesn’t necessarily produce more accurate results (Ward et al, 2018). Furthermore, we tried to acquire a more detailed life cycle inventory for GPUs (e.g. H100) to account for emissions from GPUs using *a hybrid LCA approach*. Because the data was either was confidential or not reliable, we decided not to conduct a hybrid LCA. Due to these data restrictions, we estimated GPU related emissions using input-output LCA (excluding the end-of-life phase).

Regarding the ISO 14040/44 phases: the functional unit for the estimation is “one GPU over its operational lifetime (assumed 5 years). System boundary excludes the “end-of-life” phase due to lack of data. Results are not intended for direct comparison between specific GPU models or manufacturers. It rather serves for estimation for a global scale carbon accounting from LLM model’s around the world. Foreground GPU manufacturing cost were derived literature and life cycle inventory from input-output based approaches using semiconductor industry and compared to industry reports where appliable. So, the main Life cycle inventory is EXIOBASE’s semiconductor sector. Results fall within the range reported by prior semiconductor and accelerator LCAs, supporting inventory plausibility. (Supplementary Information Table 45)Climate change impacts were assessed using IPCC AR6 GWP100, consistent with current LCA practice for energy-intensive ICT systems. Results are indicative and subject to uncertainty related to proprietary semiconductor manufacturing data, which is not a public data. As the study does not make comparative assertions between specific products or manufacturers, an external critical review is not required under ISO 14044.

GPU manufacturing emissions are modeled at the sectoral and demand-driven level, consistent with the MRIO modeling approaches. Specifically, GPU production enters the model as an aggregate upstream requirement induced by scenario-specific AI energy demand, rather than as a standalone product system with a defined functional unit. Manufacturing emissions are estimated using sector-level emission intensities derived from EXIOBASE, multiplied by the modeled scale of GPU demand under each scenario. This approach is designed to ensure internally consistent accounting of embodied

emissions across alternative AI deployment pathways, not to compare the life-cycle footprint of a specific GPU model, manufacturer, or technology generation.

Ward, H., Wenz, L., Steckel, J. C., & Minx, J. C. (2018). Truncation error estimates in process life cycle assessment using input-output analysis. *Journal of Industrial Ecology*, *22*(5), 1080-1091.

No error propagation or confidence intervals reported for emission totals.

Response:

The absence of formal error propagation and confidence intervals reflects a deliberate methodological choice, aligned with the scenario-based and sector-level nature of the analysis.

In this study, uncertainty in AI-related emissions is primarily driven by structural and behavioral factors rather than by variability in emission coefficients. These sources of uncertainty include differences in AI deployment architectures, heterogeneous regional adoption trajectories, projected paths of hardware efficiency improvements, and alternative assumptions regarding the pace and extent of electricity system decarbonization. Together, these factors account for the dominant variation in long-term emissions outcomes and are explicitly represented through multiple contrasting scenarios, which constitute the primary analytical dimension of the paper. The MRIO framework is subsequently used as an accounting mechanism to translate scenario-specific energy demand into life-cycle emissions, rather than as a stochastic model subject to parameter estimation uncertainty.

Introducing statistical error propagation at the MRIO level would imply that uncertainty in emission coefficients dominates the results. In practice, however, variation across scenarios exceeds plausible variation in sector-level emission intensities by orders of magnitude, and probabilistic treatment of MRIO coefficients would not materially alter the comparative conclusions.

Moreover, confidence intervals are most informative for product-level or parameter-estimation problems. In contrast, this study addresses system-level, forward-looking scenarios, where uncertainty is fundamentally epistemic and policy-driven rather than statistical. Representing uncertainty through scenario envelopes therefore provides a more transparent and policy-relevant characterization than reporting narrow confidence bounds around point estimates.

We acknowledge that stochastic MRIO formulations and probabilistic uncertainty propagation represent valuable directions for future research. However, given the objectives of this study, to compare long-run emissions outcomes across alternative AI deployment and energy transition pathways, we consider scenario-based uncertainty representation to be the most appropriate methodological choice.

Results
Global Energy Demand Scenarios
Results are presented as deterministic forecasts though input assumptions are random.
Carbon Footprint Breakdown

All results are explicitly framed as conditional on scenario assumptions. Shaded bands in Figures 2 and 3 represent uncertainty ranges.

No benchmarking against existing sectoral projections (IEA, IPCC, or McKinsey AI energy reports).

IEA and IPCC projections do not disaggregate AI efficiency, computational demand, or model deployment scenarios at the level required for this analysis. Our framework is complementary to, rather than duplicative of, sector-level energy outlooks.

Country-level disparities are interesting but derived from proxy variables (IHDI) instead of actual digital infrastructure metrics.

Country-specific digital infrastructure calibration is incorporated by accounting for the number of data centers, as outlined in the Supplementary Section 7.2.1.

Tables referenced in Supplementary Information are essential to credibility but not summarized in the main text.

The main text references and synthesizes the key findings from the Supplementary Information. Detailed tables are placed in the Supplementary section to maintain readability while ensuring transparency. They are now fully referenced in the manuscript, particularly in the Energy efficiency, Computational demand, and Total power consumption sections, which use data for projections.

Tables and Figures
Tables are descriptive but do not include units, uncertainty, or data sources.

Units, data sources, and uncertainty representations are provided either in figure captions or in the Supplementary Information. Shaded bands in figures denote uncertainty ranges derived from scenario variability, as described in the Methods.

Figures 2-3 show projections but lack axis units and confidence interval methods.

Axis units and confidence interval definitions are already provided in both the figure panels and the captions. Specifically, all axes are labeled with physical units (e.g., FLOPs, models per year, TWh/year, percentage of global electricity), and the shaded bands are explicitly identified in the captions as 90% confidence intervals. These intervals arise from parametric uncertainty in calibrated model inputs and are propagated through repeated simulations, as described in the Methods section. We have ensured this is clearly stated in the text accompanying the figures.

Figures 2-6 lack legends detailing whether shaded bands represent model variance or scenario spread.

The distinction between uncertainty sources is already embedded in the figure design. Shaded bands in Figures 2 and 3 represent within-scenario parametric uncertainty, as indicated in the captions, while differences across curves and panels correspond to discrete scenario assumptions. Figures 4–6 do not include shaded bands by design, as they report deterministic outcomes conditional on each scenario. We have clarified this interpretive distinction in the manuscript to ensure readers do not conflate scenario variation with statistical variance.

Results in Figs 4-6 depend on unvalidated parameters.

The purpose of this study is not to produce point forecasts conditional on a specific parameter realization, but to examine how alternative structural configurations of AI development and electricity systems shape long-run energy demand and emissions outcomes. Parameter values are calibrated to documented historical ranges and engineering benchmarks, as described in the Supplementary Information, and are held consistently across scenarios.

The central results of the paper concern relative comparisons across scenarios, rather than absolute numerical levels. In particular, the ordering of outcomes across deployment structures and electricity mix assumptions, as well as the dominance of deployment and energy-system pathways over efficiency improvements in determining long-run carbon intensity, is robust to the specific parameter realizations within empirically plausible ranges. The conclusions therefore follow from the modeled relationships between computational demand, efficiency trajectories, and electricity systems, rather than from any single parameter choice.

Accordingly, the analysis should be interpreted as identifying structural drivers and trade-offs in AI-related energy demand and emissions under alternative development pathways, rather than as validating precise long-term predictions.

Discussion
Reads more like general ideas instead of being supported by data, analysis, or evidence.

We appreciate this comment and have substantially revised the Discussion to more explicitly ground interpretive statements in the quantitative results of the study. The revised Discussion now systematically references scenario-specific outcomes, including projected ranges of AI electricity demand, emissions magnitudes, and relative differences across deployment architectures and electricity mix assumptions. Assertions regarding the dominance of training-related emissions, the comparative performance of centralized versus decentralized model scaling, and the role of electricity system decarbonization are now explicitly supported by results presented in Figures 2–6 and corresponding numerical values reported in the Results and Supplementary Information. These revisions ensure that the Discussion synthesizes and interprets the model outputs rather than presenting general or speculative commentary.

Insufficient linkage to numerical findings.

We appreciate this comment and have revised the Discussion to more explicitly connect interpretive claims to the quantitative results presented in the Results section. The Discussion now consistently references specific scenario comparisons and figures, with an emphasis on explaining relative differences across scenarios rather than advancing absolute forecasts. This framing reflects the scenario-based nature of the analysis and clarifies how the qualitative interpretations follow directly from the modeled numerical outcomes.

Circular reasoning: claims that “efficiency gains alone will not suffice” rest on model assumptions, not strong validation.

We thank the reviewer for raising this concern and have clarified the logic underlying our conclusions about efficiency gains. The finding that efficiency improvements alone are insufficient does not stem from an a priori assumption, but from explicit cross-scenario comparisons in which efficiency trajectories are held constant while deployment structure and electricity mix vary. The resulting divergence in energy use and emissions reflects modeled interactions between efficiency, scaling behavior, and grid carbon intensity, which are now more clearly articulated in the Discussion and Conclusion.

Exclusion of economic and policy feasibility dimensions weakens the practical relevance.

We agree that questions of economic and political feasibility are important, but they fall outside the scope of the present modeling framework. We have clarified this boundary more explicitly in the Discussion. The intent of the study is not to evaluate feasibility or optimal policy design, but to provide quantitative bounds on potential energy and emissions outcomes under different deployment and energy system pathways. These results are intended to inform subsequent economic, political, and institutional analyses rather than substitute for them.

Several citations (30-35) are referenced but not elaborated; end-of-life and circular economy aspects are superficial.

In the revised manuscript, we have expanded the discussion surrounding references 30–35 to more clearly articulate their relevance to the present analysis. In particular, these citations are now explicitly linked to embodied emissions in hardware manufacturing, infrastructure lifetimes, and emerging mitigation strategies rather than being listed as general background. We also clarified the role of end-of-life and circular economy considerations as contextual and forward-looking extensions, rather than core components of the quantitative model. Given the limited availability of consistent global data on reuse, recycling rates, and secondary markets for AI hardware, these aspects are intentionally discussed qualitatively and framed as important directions for future research rather than as quantitatively modeled pathways.

Conclusion
Mostly a repetition of limitations.

The Conclusion has been revised to more clearly synthesize the study's core findings and their implications, rather than focusing primarily on methodological limitations. The updated text highlights that AI's long-term energy use and emissions are governed by the interaction between deployment structure, electricity system decarbonization, and the geographic concentration of compute, with efficiency improvements alone proving insufficient. It now summarizes key contrasts across scenarios, emphasizing the comparatively larger emissions reductions achievable through structural interventions such as model consolidation and low-carbon electricity transitions. Remaining uncertainties and data limitations inherent to long-horizon scenario analysis are retained but more directly linked to the interpretation of results, and the exploratory (non-predictive) nature of the scenarios is clarified alongside the resulting policy relevance.

No actionable policy guidance or quantifiable thresholds (e.g., emission reduction targets, scenario prioritization).

The manuscript provides comparative insights into which structural factors dominate emissions outcomes. Prescriptive policy design is beyond the scope of this paper.

Shifts from cautious projection to assertive prediction (“AI workloads could consume 30 % of global electricity”) without strong evidence.

We appreciate this comment and agree that maintaining a clear distinction between scenario-based projections and predictive claims is important. In the revised manuscript, we have carefully reviewed the language throughout the Results and Discussion to ensure consistency with the exploratory and conditional nature of the modeling framework. Statements that could be interpreted as deterministic predictions have been revised to explicitly emphasize their dependence on scenario assumptions, calibrated parameter ranges, and electricity system trajectories. In particular, phrases implying certainty have been replaced with conditional formulations that refer to upper-bound outcomes under specific high-growth scenarios rather than general forecasts. This ensures that quantitative estimates such as the 30% figure are clearly presented as scenario-contingent results rather than assertive predictions.

References
Formatting inconsistent.

Minor reference formatting issues were corrected to improve consistency and robustness. Specifically, a Unicode ellipsis in the EXIOBASE dataset citation was replaced with standard author truncation to ensure compatibility with bibliographic processing, a missing “In” was added to one conference proceeding reference for consistency

Depends on secondary sources (Forbes, Statista-type data) rather than peer-reviewed energy or computing journals.

We appreciate the opportunity to clarify this point. The Forbes articles cited in the manuscript are used exclusively in the introductory and motivating sections to illustrate the prominence of AI energy concerns in public and policy discourse. They are not used as data sources, calibration inputs, or analytical foundations for any quantitative results.

All numerical inputs, parameter calibrations, and empirical relationships used in the modeling framework are derived from peer-reviewed literature, established databases, or authoritative technical sources. These sources are documented in detail in the Supplementary Information, including peer-reviewed studies on hardware efficiency, algorithmic scaling laws, data center performance, adoption dynamics, and life-cycle carbon accounting, as well as standardized datasets such as EXIOBASE and World Bank records.

Insufficient citation of existing MRIO and integrated assessment models (e.g., GTAP-E, GCAM, or IAMC frameworks).

Relevant MRIO literature has been added, including:

- Yang et al. (2025), which supports the treatment of embodied renewable energy and global trade linkages within the multi-regional input–output framework.
- Lu and Chen (2025), which provides methodological grounding for the spatially disaggregated MRIO modeling of power sector emissions used in the regional analysis.
- Faturay et al. (2020), which contextualizes the application of MRIO models to energy system transitions and supports the treatment of upstream economic and energy impacts.
