# Supplementary material for: Scenario-based forecasting of the global energy demand and carbon footprint of artificial intelligence
Source: PLoS One. 2026 Mar 11;21(3):e0343056. doi: 10.1371/journal.pone.0343056 (PMC12978478; doi:10.1371/journal.pone.0343056)
Supplement: Attachment — Submitted filename: reviewer comments.docx [file pone.0343056.s002.docx]

**Scenario-based forecasting of the global energy demand and carbon footprint of**

**artificial intelligence**

**Title**

Unspecified, “Scenario-based forecasting of the global energy demand and carbon footprint of AI” fails to indicate the methodological novelty (e.g., MRIO integration, six-scenario modeling) or specific time horizon.

Does not distinguish whether the analysis is empirical, simulation-based, or theoretical, reducing clarity for readers searching for methodological studies.

Please add study design.

**Abstract**

Missing essential methodological transparency (e.g., model validation, uncertainty quantification).

No clear mention of data sources or the calibration framework for efficiency parameters.

Policy implications are asserted (“efficiency gains alone will not suffice”) but not supported by a quantitative comparison in the abstract.

Sentence structure is long and complex, affecting readability.

Keywords: Many and lacking prioritization. Some are descriptive rather than functional (“technology adoption” is unclear).

Missing terms that reflect the study’s modeling approach (e.g., “scenario analysis,” “sustainability modeling,” “AI energy systems”).

**Introduction**

Lengthy and redundant. The background has many pages and repeats data center energy statistics.

Limited critical synthesis of prior scenario-based energy studies or integrated assessment models (IAMs); references 5-8 are not analyzed comparatively.

The knowledge gap is not articulated; it is unclear whether the novelty lies in global scope, scenario design, or MRIO coupling.

Unsupported generalizations, such as “AI currently represents approximately 0.4% of global electricity consumption,” are presented without citing precise datasets or year references.

No clear conceptual framework figure to orient the reader before reading the “Methods.”

**Materials and Methods**

**Model Framework**

The structure (Fig. 1) is descriptive but there are no validation steps or uncertainty propagation techniques (e.g., Monte Carlo or sensitivity analysis).

No justification for selecting exponential and logistic functional forms for model scaling and efficiency gains.

**Energy Efficiency Modeling**

Equations (1) to (4) are complex but empirical parameters are not justified nor referenced.

Independence assumptions among hardware, algorithmic, and infrastructural efficiencies (Eq. 4) are strong and unsupported.

No validation using real-world benchmarks (e.g., GPU generation trends or data center efficiency reports).

**Computational Demand**

Depends on compound exponential extrapolation of model complexity and query volume.

Population and adoption rates (Eq. 10) use logistic diffusion but no region-specific calibration is shown.

Data sources unclear (Supplementary Sections cited but not summarized in text).

**MRIO Analysis**

The MRIO methodology is standard, but no sensitivity testing or temporal updating of EXIOBASE is described.

Attribution of GPU manufacturing entirely to Taiwan is over-simplified; ignores TSMC Arizona, Samsung Texas, or emerging EU fabs.

**Carbon Footprint Estimation**

Equations (15) to (21) are mathematically correct but no validation against known industry LCA benchmarks (e.g., ISO 14040 standards).

No error propagation or confidence intervals reported for emission totals.

**Results**

**Global Energy Demand Scenarios**

Results are presented as deterministic forecasts though input assumptions are random.

**Carbon Footprint Breakdown**

No benchmarking against existing sectoral projections (IEA, IPCC, or McKinsey AI energy reports).

Country-level disparities are interesting but derived from proxy variables (IHDI) instead of actual digital infrastructure metrics.

Tables referenced in Supplementary Information are essential to credibility but not summarized in the main text.

**Tables and Figures**

Tables are descriptive but do not include units, uncertainty, or data sources.

Figures 2-3 show projections but lack axis units and confidence interval methods.

Figures 2-6 lack legends detailing whether shaded bands represent model variance or scenario spread.

Results in Figs 4-6 depend on unvalidated parameters.

**Discussion**

Reads more like general ideas instead of being supported by data, analysis, or evidence.

Insufficient linkage to numerical findings.

Circular reasoning: claims that “efficiency gains alone will not suffice” rest on model assumptions, not strong validation.

Exclusion of economic and policy feasibility dimensions weakens the practical relevance.

Several citations (30-35) are referenced but not elaborated; end-of-life and circular economy aspects are superficial.

**Conclusion**

Mostly a repetition of limitations.

No actionable policy guidance or quantifiable thresholds (e.g., emission reduction targets, scenario prioritization).

Shifts from cautious projection to assertive prediction (“AI workloads could consume 30 % of global electricity”) without strong evidence.

**References**

Formatting inconsistent.

Depends on secondary sources (Forbes, Statista-type data) rather than peer-reviewed energy or computing journals.

Insufficient citation of existing MRIO and integrated assessment models (e.g., GTAP-E, GCAM, or IAMC frameworks).
